# Supplementary material for: Resolving the full spectrum of human genome variation using Linked-Reads
Source: Genome Res. 2019 Apr;29(4):635–45. doi: 10.1101/gr.234443.118 (PMC6442396; doi:10.1101/gr.234443.118)
Supplement: Supplemental Material [file supp_gr.234443.118_Supplemental_longranger-2.2.zip › longranger-2.2.tar.bz2!/lr2.2-public/lib/stan/cmdstan/stan/doc/api/html/namespacestan_1_1prob.html]

Stan: stan::prob Namespace Reference


|  |  |
| --- | --- |
|  | Stan  2.5.0  probability, sampling & optimization |


- Main Page
- Related Pages
- Namespaces
- Classes
- Files

- Namespace List
- Namespace Members

All Classes Namespaces Files Functions Variables Typedefs Enumerations Enumerator Friends Macros Pages

- stan
- prob

Classes |
Functions |
Variables

stan::prob Namespace Reference

Templated probability distributions.
More...

|  |  |
| --- | --- |
| Classes | |
| struct | include\_summand |
|  | Template metaprogram to calculate whether a summand needs to be included in a proportional (log) probability calculation. More... |
|  | |
| class | welford\_covar\_estimator |
|  | |
| class | welford\_var\_estimator |
|  | |

|  |  |
| --- | --- |
| Functions | |
| template<bool propto, typename T\_y , typename T\_loc , typename T\_scale > | |
| boost::enable\_if\_c  < contains\_fvar< T\_y, T\_loc,   T\_scale >::value, typename   return\_type< T\_y, T\_loc,   T\_scale >::type >::type | normal\_log (const T\_y &y, const T\_loc &mu, const T\_scale &sigma) |
|  | |
| template<typename T > | |
| void | autocorrelation (const std::vector< T > &y, std::vector< T > &ac, Eigen::FFT< T > &fft) |
|  | Write autocorrelation estimates for every lag for the specified input sequence into the specified result using the specified FFT engine. More... |
|  | |
| template<typename T > | |
| void | autocorrelation (const std::vector< T > &y, std::vector< T > &ac) |
|  | Write autocorrelation estimates for every lag for the specified input sequence into the specified result. More... |
|  | |
| template<typename T > | |
| void | autocovariance (const std::vector< T > &y, std::vector< T > &acov, Eigen::FFT< T > &fft) |
|  | Write autocovariance estimates for every lag for the specified input sequence into the specified result using the specified FFT engine. More... |
|  | |
| template<typename T > | |
| void | autocovariance (const std::vector< T > &y, std::vector< T > &acov) |
|  | Write autocovariance estimates for every lag for the specified input sequence into the specified result. More... |
|  | |
| template<bool propto, typename T\_prob , typename T\_prior\_sample\_size > | |
| boost::math::tools::promote\_args  < T\_prob, T\_prior\_sample\_size >  ::type | dirichlet\_log (const Eigen::Matrix< T\_prob, Eigen::Dynamic, 1 > &theta, const Eigen::Matrix< T\_prior\_sample\_size, Eigen::Dynamic, 1 > &alpha) |
|  | The log of the Dirichlet density for the given theta and a vector of prior sample sizes, alpha. More... |
|  | |
| template<typename T\_prob , typename T\_prior\_sample\_size > | |
| boost::math::tools::promote\_args  < T\_prob, T\_prior\_sample\_size >  ::type | dirichlet\_log (const Eigen::Matrix< T\_prob, Eigen::Dynamic, 1 > &theta, const Eigen::Matrix< T\_prior\_sample\_size, Eigen::Dynamic, 1 > &alpha) |
|  | |
| template<class RNG > | |
| Eigen::VectorXd | dirichlet\_rng (const Eigen::Matrix< double, Eigen::Dynamic, 1 > &alpha, RNG &rng) |
|  | |
| template<bool propto, typename T\_y , typename T\_F , typename T\_G , typename T\_V , typename T\_W , typename T\_m0 , typename T\_C0 > | |
| return\_type< T\_y, typename   return\_type< T\_F, T\_G, T\_V,   T\_W, T\_m0, T\_C0 >::type >  ::type | gaussian\_dlm\_obs\_log (const Eigen::Matrix< T\_y, Eigen::Dynamic, Eigen::Dynamic > &y, const Eigen::Matrix< T\_F, Eigen::Dynamic, Eigen::Dynamic > &F, const Eigen::Matrix< T\_G, Eigen::Dynamic, Eigen::Dynamic > &G, const Eigen::Matrix< T\_V, Eigen::Dynamic, Eigen::Dynamic > &V, const Eigen::Matrix< T\_W, Eigen::Dynamic, Eigen::Dynamic > &W, const Eigen::Matrix< T\_m0, Eigen::Dynamic, 1 > &m0, const Eigen::Matrix< T\_C0, Eigen::Dynamic, Eigen::Dynamic > &C0) |
|  | The log of a Gaussian dynamic linear model (GDLM). More... |
|  | |
| template<typename T\_y , typename T\_F , typename T\_G , typename T\_V , typename T\_W , typename T\_m0 , typename T\_C0 > | |
| return\_type< T\_y, typename   return\_type< T\_F, T\_G, T\_V,   T\_W, T\_m0, T\_C0 >::type >  ::type | gaussian\_dlm\_obs\_log (const Eigen::Matrix< T\_y, Eigen::Dynamic, Eigen::Dynamic > &y, const Eigen::Matrix< T\_F, Eigen::Dynamic, Eigen::Dynamic > &F, const Eigen::Matrix< T\_G, Eigen::Dynamic, Eigen::Dynamic > &G, const Eigen::Matrix< T\_V, Eigen::Dynamic, Eigen::Dynamic > &V, const Eigen::Matrix< T\_W, Eigen::Dynamic, Eigen::Dynamic > &W, const Eigen::Matrix< T\_m0, Eigen::Dynamic, 1 > &m0, const Eigen::Matrix< T\_C0, Eigen::Dynamic, Eigen::Dynamic > &C0) |
|  | |
| template<bool propto, typename T\_y , typename T\_F , typename T\_G , typename T\_V , typename T\_W , typename T\_m0 , typename T\_C0 > | |
| return\_type< T\_y, typename   return\_type< T\_F, T\_G, T\_V,   T\_W, T\_m0, T\_C0 >::type >  ::type | gaussian\_dlm\_obs\_log (const Eigen::Matrix< T\_y, Eigen::Dynamic, Eigen::Dynamic > &y, const Eigen::Matrix< T\_F, Eigen::Dynamic, Eigen::Dynamic > &F, const Eigen::Matrix< T\_G, Eigen::Dynamic, Eigen::Dynamic > &G, const Eigen::Matrix< T\_V, Eigen::Dynamic, 1 > &V, const Eigen::Matrix< T\_W, Eigen::Dynamic, Eigen::Dynamic > &W, const Eigen::Matrix< T\_m0, Eigen::Dynamic, 1 > &m0, const Eigen::Matrix< T\_C0, Eigen::Dynamic, Eigen::Dynamic > &C0) |
|  | The log of a Gaussian dynamic linear model (GDLM) with uncorrelated observation disturbances. More... |
|  | |
| template<typename T\_y , typename T\_F , typename T\_G , typename T\_V , typename T\_W , typename T\_m0 , typename T\_C0 > | |
| return\_type< T\_y, typename   return\_type< T\_F, T\_G, T\_V,   T\_W, T\_m0, T\_C0 >::type >  ::type | gaussian\_dlm\_obs\_log (const Eigen::Matrix< T\_y, Eigen::Dynamic, Eigen::Dynamic > &y, const Eigen::Matrix< T\_F, Eigen::Dynamic, Eigen::Dynamic > &F, const Eigen::Matrix< T\_G, Eigen::Dynamic, Eigen::Dynamic > &G, const Eigen::Matrix< T\_V, Eigen::Dynamic, 1 > &V, const Eigen::Matrix< T\_W, Eigen::Dynamic, Eigen::Dynamic > &W, const Eigen::Matrix< T\_m0, Eigen::Dynamic, 1 > &m0, const Eigen::Matrix< T\_C0, Eigen::Dynamic, Eigen::Dynamic > &C0) |
|  | |
| template<bool propto, typename T\_y , typename T\_dof , typename T\_scale > | |
| boost::math::tools::promote\_args  < T\_y, T\_dof, T\_scale >::type | inv\_wishart\_log (const Eigen::Matrix< T\_y, Eigen::Dynamic, Eigen::Dynamic > &W, const T\_dof &nu, const Eigen::Matrix< T\_scale, Eigen::Dynamic, Eigen::Dynamic > &S) |
|  | The log of the Inverse-Wishart density for the given W, degrees of freedom, and scale matrix. More... |
|  | |
| template<typename T\_y , typename T\_dof , typename T\_scale > | |
| boost::math::tools::promote\_args  < T\_y, T\_dof, T\_scale >::type | inv\_wishart\_log (const Eigen::Matrix< T\_y, Eigen::Dynamic, Eigen::Dynamic > &W, const T\_dof &nu, const Eigen::Matrix< T\_scale, Eigen::Dynamic, Eigen::Dynamic > &S) |
|  | |
| template<class RNG > | |
| Eigen::Matrix< double,   Eigen::Dynamic, Eigen::Dynamic > | inv\_wishart\_rng (const double nu, const Eigen::Matrix< double, Eigen::Dynamic, Eigen::Dynamic > &S, RNG &rng) |
|  | |
| template<typename T\_shape > | |
| T\_shape | do\_lkj\_constant (const T\_shape &eta, const unsigned int &K) |
|  | |
| template<bool propto, typename T\_covar , typename T\_shape > | |
| boost::math::tools::promote\_args  < T\_covar, T\_shape >::type | lkj\_corr\_cholesky\_log (const Eigen::Matrix< T\_covar, Eigen::Dynamic, Eigen::Dynamic > &L, const T\_shape &eta) |
|  | |
| template<typename T\_covar , typename T\_shape > | |
| boost::math::tools::promote\_args  < T\_covar, T\_shape >::type | lkj\_corr\_cholesky\_log (const Eigen::Matrix< T\_covar, Eigen::Dynamic, Eigen::Dynamic > &L, const T\_shape &eta) |
|  | |
| template<bool propto, typename T\_y , typename T\_shape > | |
| boost::math::tools::promote\_args  < T\_y, T\_shape >::type | lkj\_corr\_log (const Eigen::Matrix< T\_y, Eigen::Dynamic, Eigen::Dynamic > &y, const T\_shape &eta) |
|  | |
| template<typename T\_y , typename T\_shape > | |
| boost::math::tools::promote\_args  < T\_y, T\_shape >::type | lkj\_corr\_log (const Eigen::Matrix< T\_y, Eigen::Dynamic, Eigen::Dynamic > &y, const T\_shape &eta) |
|  | |
| template<class RNG > | |
| Eigen::MatrixXd | lkj\_corr\_cholesky\_rng (const size\_t K, const double eta, RNG &rng) |
|  | |
| template<class RNG > | |
| Eigen::MatrixXd | lkj\_corr\_rng (const size\_t K, const double eta, RNG &rng) |
|  | |
| template<bool propto, typename T\_y , typename T\_loc , typename T\_scale , typename T\_shape > | |
| boost::math::tools::promote\_args  < T\_y, T\_loc, T\_scale, T\_shape >  ::type | lkj\_cov\_log (const Eigen::Matrix< T\_y, Eigen::Dynamic, Eigen::Dynamic > &y, const Eigen::Matrix< T\_loc, Eigen::Dynamic, 1 > &mu, const Eigen::Matrix< T\_scale, Eigen::Dynamic, 1 > &sigma, const T\_shape &eta) |
|  | |
| template<typename T\_y , typename T\_loc , typename T\_scale , typename T\_shape > | |
| boost::math::tools::promote\_args  < T\_y, T\_loc, T\_scale, T\_shape >  ::type | lkj\_cov\_log (const Eigen::Matrix< T\_y, Eigen::Dynamic, Eigen::Dynamic > &y, const Eigen::Matrix< T\_loc, Eigen::Dynamic, 1 > &mu, const Eigen::Matrix< T\_scale, Eigen::Dynamic, 1 > &sigma, const T\_shape &eta) |
|  | |
| template<bool propto, typename T\_y , typename T\_loc , typename T\_scale , typename T\_shape > | |
| boost::math::tools::promote\_args  < T\_y, T\_loc, T\_scale, T\_shape >  ::type | lkj\_cov\_log (const Eigen::Matrix< T\_y, Eigen::Dynamic, Eigen::Dynamic > &y, const T\_loc &mu, const T\_scale &sigma, const T\_shape &eta) |
|  | |
| template<typename T\_y , typename T\_loc , typename T\_scale , typename T\_shape > | |
| boost::math::tools::promote\_args  < T\_y, T\_loc, T\_scale, T\_shape >  ::type | lkj\_cov\_log (const Eigen::Matrix< T\_y, Eigen::Dynamic, Eigen::Dynamic > &y, const T\_loc &mu, const T\_scale &sigma, const T\_shape &eta) |
|  | |
| template<bool propto, typename T\_y , typename T\_Mu , typename T\_Sigma , typename T\_D > | |
| boost::math::tools::promote\_args  < T\_y, T\_Mu, T\_Sigma, T\_D >  ::type | matrix\_normal\_prec\_log (const Eigen::Matrix< T\_y, Eigen::Dynamic, Eigen::Dynamic > &y, const Eigen::Matrix< T\_Mu, Eigen::Dynamic, Eigen::Dynamic > &Mu, const Eigen::Matrix< T\_Sigma, Eigen::Dynamic, Eigen::Dynamic > &Sigma, const Eigen::Matrix< T\_D, Eigen::Dynamic, Eigen::Dynamic > &D) |
|  | The log of the matrix normal density for the given y, mu, Sigma and D where Sigma and D are given as precision matrices, not covariance matrices. More... |
|  | |
| template<typename T\_y , typename T\_Mu , typename T\_Sigma , typename T\_D > | |
| boost::math::tools::promote\_args  < T\_y, T\_Mu, T\_Sigma, T\_D >  ::type | matrix\_normal\_prec\_log (const Eigen::Matrix< T\_y, Eigen::Dynamic, Eigen::Dynamic > &y, const Eigen::Matrix< T\_Mu, Eigen::Dynamic, Eigen::Dynamic > &Mu, const Eigen::Matrix< T\_Sigma, Eigen::Dynamic, Eigen::Dynamic > &Sigma, const Eigen::Matrix< T\_D, Eigen::Dynamic, Eigen::Dynamic > &D) |
|  | |
| template<bool propto, typename T\_y , typename T\_covar , typename T\_w > | |
| boost::math::tools::promote\_args  < T\_y, T\_covar, T\_w >::type | multi\_gp\_log (const Eigen::Matrix< T\_y, Eigen::Dynamic, Eigen::Dynamic > &y, const Eigen::Matrix< T\_covar, Eigen::Dynamic, Eigen::Dynamic > &Sigma, const Eigen::Matrix< T\_w, Eigen::Dynamic, 1 > &w) |
|  | The log of a multivariate Gaussian Process for the given y, Sigma, and w. More... |
|  | |
| template<typename T\_y , typename T\_covar , typename T\_w > | |
| boost::math::tools::promote\_args  < T\_y, T\_covar, T\_w >::type | multi\_gp\_log (const Eigen::Matrix< T\_y, Eigen::Dynamic, Eigen::Dynamic > &y, const Eigen::Matrix< T\_covar, Eigen::Dynamic, Eigen::Dynamic > &Sigma, const Eigen::Matrix< T\_w, Eigen::Dynamic, 1 > &w) |
|  | |
| template<bool propto, typename T\_y , typename T\_covar , typename T\_w > | |
| boost::math::tools::promote\_args  < T\_y, T\_covar, T\_w >::type | multi\_gp\_cholesky\_log (const Eigen::Matrix< T\_y, Eigen::Dynamic, Eigen::Dynamic > &y, const Eigen::Matrix< T\_covar, Eigen::Dynamic, Eigen::Dynamic > &L, const Eigen::Matrix< T\_w, Eigen::Dynamic, 1 > &w) |
|  | The log of a multivariate Gaussian Process for the given y, w, and a Cholesky factor L of the kernel matrix Sigma. More... |
|  | |
| template<typename T\_y , typename T\_covar , typename T\_w > | |
| boost::math::tools::promote\_args  < T\_y, T\_covar, T\_w >::type | multi\_gp\_cholesky\_log (const Eigen::Matrix< T\_y, Eigen::Dynamic, Eigen::Dynamic > &y, const Eigen::Matrix< T\_covar, Eigen::Dynamic, Eigen::Dynamic > &L, const Eigen::Matrix< T\_w, Eigen::Dynamic, 1 > &w) |
|  | |
| template<bool propto, typename T\_y , typename T\_loc , typename T\_covar > | |
| boost::math::tools::promote\_args  < typename scalar\_type< T\_y >  ::type, typename scalar\_type  < T\_loc >::type, T\_covar >  ::type | multi\_normal\_log (const T\_y &y, const T\_loc &mu, const Eigen::Matrix< T\_covar, Eigen::Dynamic, Eigen::Dynamic > &Sigma) |
|  | |
| template<typename T\_y , typename T\_loc , typename T\_covar > | |
| boost::math::tools::promote\_args  < typename scalar\_type< T\_y >  ::type, typename scalar\_type  < T\_loc >::type, T\_covar >  ::type | multi\_normal\_log (const T\_y &y, const T\_loc &mu, const Eigen::Matrix< T\_covar, Eigen::Dynamic, Eigen::Dynamic > &Sigma) |
|  | |
| template<class RNG > | |
| Eigen::VectorXd | multi\_normal\_rng (const Eigen::Matrix< double, Eigen::Dynamic, 1 > &mu, const Eigen::Matrix< double, Eigen::Dynamic, Eigen::Dynamic > &S, RNG &rng) |
|  | |
| template<bool propto, typename T\_y , typename T\_loc , typename T\_covar > | |
| boost::math::tools::promote\_args  < typename scalar\_type< T\_y >  ::type, typename scalar\_type  < T\_loc >::type, T\_covar >  ::type | multi\_normal\_cholesky\_log (const T\_y &y, const T\_loc &mu, const Eigen::Matrix< T\_covar, Eigen::Dynamic, Eigen::Dynamic > &L) |
|  | The log of the multivariate normal density for the given y, mu, and a Cholesky factor L of the variance matrix. More... |
|  | |
| template<typename T\_y , typename T\_loc , typename T\_covar > | |
| boost::math::tools::promote\_args  < typename scalar\_type< T\_y >  ::type, typename scalar\_type  < T\_loc >::type, T\_covar >  ::type | multi\_normal\_cholesky\_log (const T\_y &y, const T\_loc &mu, const Eigen::Matrix< T\_covar, Eigen::Dynamic, Eigen::Dynamic > &L) |
|  | |
| template<class RNG > | |
| Eigen::VectorXd | multi\_normal\_cholesky\_rng (const Eigen::Matrix< double, Eigen::Dynamic, 1 > &mu, const Eigen::Matrix< double, Eigen::Dynamic, Eigen::Dynamic > &S, RNG &rng) |
|  | |
| template<bool propto, typename T\_y , typename T\_loc , typename T\_covar > | |
| boost::math::tools::promote\_args  < typename scalar\_type< T\_y >  ::type, typename scalar\_type  < T\_loc >::type, T\_covar >  ::type | multi\_normal\_prec\_log (const T\_y &y, const T\_loc &mu, const Eigen::Matrix< T\_covar, Eigen::Dynamic, Eigen::Dynamic > &Sigma) |
|  | |
| template<typename T\_y , typename T\_loc , typename T\_covar > | |
| boost::math::tools::promote\_args  < typename scalar\_type< T\_y >  ::type, typename scalar\_type  < T\_loc >::type, T\_covar >  ::type | multi\_normal\_prec\_log (const T\_y &y, const T\_loc &mu, const Eigen::Matrix< T\_covar, Eigen::Dynamic, Eigen::Dynamic > &Sigma) |
|  | |
| template<bool propto, typename T\_y , typename T\_dof , typename T\_loc , typename T\_scale > | |
| boost::math::tools::promote\_args  < typename scalar\_type< T\_y >  ::type, T\_dof, typename   scalar\_type< T\_loc >::type,   T\_scale >::type | multi\_student\_t\_log (const T\_y &y, const T\_dof &nu, const T\_loc &mu, const Eigen::Matrix< T\_scale, Eigen::Dynamic, Eigen::Dynamic > &Sigma) |
|  | Return the log of the multivariate Student t distribution at the specified arguments. More... |
|  | |
| template<typename T\_y , typename T\_dof , typename T\_loc , typename T\_scale > | |
| boost::math::tools::promote\_args  < typename scalar\_type< T\_y >  ::type, T\_dof, typename   scalar\_type< T\_loc >::type,   T\_scale >::type | multi\_student\_t\_log (const T\_y &y, const T\_dof &nu, const T\_loc &mu, const Eigen::Matrix< T\_scale, Eigen::Dynamic, Eigen::Dynamic > &Sigma) |
|  | |
| template<class RNG > | |
| Eigen::VectorXd | multi\_student\_t\_rng (const double nu, const Eigen::Matrix< double, Eigen::Dynamic, 1 > &mu, const Eigen::Matrix< double, Eigen::Dynamic, Eigen::Dynamic > &s, RNG &rng) |
|  | |
| template<bool propto, typename T\_y , typename T\_dof , typename T\_scale > | |
| boost::math::tools::promote\_args  < T\_y, T\_dof, T\_scale >::type | wishart\_log (const Eigen::Matrix< T\_y, Eigen::Dynamic, Eigen::Dynamic > &W, const T\_dof &nu, const Eigen::Matrix< T\_scale, Eigen::Dynamic, Eigen::Dynamic > &S) |
|  | The log of the Wishart density for the given W, degrees of freedom, and scale matrix. More... |
|  | |
| template<typename T\_y , typename T\_dof , typename T\_scale > | |
| boost::math::tools::promote\_args  < T\_y, T\_dof, T\_scale >::type | wishart\_log (const Eigen::Matrix< T\_y, Eigen::Dynamic, Eigen::Dynamic > &W, const T\_dof &nu, const Eigen::Matrix< T\_scale, Eigen::Dynamic, Eigen::Dynamic > &S) |
|  | |
| template<class RNG > | |
| Eigen::Matrix< double,   Eigen::Dynamic, Eigen::Dynamic > | wishart\_rng (const double nu, const Eigen::Matrix< double, Eigen::Dynamic, Eigen::Dynamic > &S, RNG &rng) |
|  | |
| template<bool propto, typename T\_prob > | |
| boost::math::tools::promote\_args  < T\_prob >::type | categorical\_log (int n, const Eigen::Matrix< T\_prob, Eigen::Dynamic, 1 > &theta) |
|  | |
| template<typename T\_prob > | |
| boost::math::tools::promote\_args  < T\_prob >::type | categorical\_log (const typename math::index\_type< Eigen::Matrix< T\_prob, Eigen::Dynamic, 1 > >::type n, const Eigen::Matrix< T\_prob, Eigen::Dynamic, 1 > &theta) |
|  | |
| template<bool propto, typename T\_prob > | |
| boost::math::tools::promote\_args  < T\_prob >::type | categorical\_log (const std::vector< int > &ns, const Eigen::Matrix< T\_prob, Eigen::Dynamic, 1 > &theta) |
|  | |
| template<typename T\_prob > | |
| boost::math::tools::promote\_args  < T\_prob >::type | categorical\_log (const std::vector< int > &ns, const Eigen::Matrix< T\_prob, Eigen::Dynamic, 1 > &theta) |
|  | |
| template<class RNG > | |
| int | categorical\_rng (const Eigen::Matrix< double, Eigen::Dynamic, 1 > &theta, RNG &rng) |
|  | |
| template<bool propto, typename T\_prob > | |
| boost::math::tools::promote\_args  < T\_prob >::type | categorical\_logit\_log (int n, const Eigen::Matrix< T\_prob, Eigen::Dynamic, 1 > &beta) |
|  | |
| template<typename T\_prob > | |
| boost::math::tools::promote\_args  < T\_prob >::type | categorical\_logit\_log (int n, const Eigen::Matrix< T\_prob, Eigen::Dynamic, 1 > &beta) |
|  | |
| template<bool propto, typename T\_prob > | |
| boost::math::tools::promote\_args  < T\_prob >::type | categorical\_logit\_log (const std::vector< int > &ns, const Eigen::Matrix< T\_prob, Eigen::Dynamic, 1 > &beta) |
|  | |
| template<typename T\_prob > | |
| boost::math::tools::promote\_args  < T\_prob >::type | categorical\_logit\_log (const std::vector< int > &ns, const Eigen::Matrix< T\_prob, Eigen::Dynamic, 1 > &beta) |
|  | |
| template<bool propto, typename T\_prob > | |
| boost::math::tools::promote\_args  < T\_prob >::type | multinomial\_log (const std::vector< int > &ns, const Eigen::Matrix< T\_prob, Eigen::Dynamic, 1 > &theta) |
|  | |
| template<typename T\_prob > | |
| boost::math::tools::promote\_args  < T\_prob >::type | multinomial\_log (const std::vector< int > &ns, const Eigen::Matrix< T\_prob, Eigen::Dynamic, 1 > &theta) |
|  | |
| template<class RNG > | |
| std::vector< int > | multinomial\_rng (const Eigen::Matrix< double, Eigen::Dynamic, 1 > &theta, const int N, RNG &rng) |
|  | |
| template<bool propto, typename T\_y , typename T\_scale\_succ , typename T\_scale\_fail > | |
| return\_type< T\_y, T\_scale\_succ,   T\_scale\_fail >::type | beta\_log (const T\_y &y, const T\_scale\_succ &alpha, const T\_scale\_fail &beta) |
|  | The log of the beta density for the specified scalar(s) given the specified sample size(s). More... |
|  | |
| template<typename T\_y , typename T\_scale\_succ , typename T\_scale\_fail > | |
| return\_type< T\_y, T\_scale\_succ,   T\_scale\_fail >::type | beta\_log (const T\_y &y, const T\_scale\_succ &alpha, const T\_scale\_fail &beta) |
|  | |
| template<typename T\_y , typename T\_scale\_succ , typename T\_scale\_fail > | |
| return\_type< T\_y, T\_scale\_succ,   T\_scale\_fail >::type | beta\_cdf (const T\_y &y, const T\_scale\_succ &alpha, const T\_scale\_fail &beta) |
|  | Calculates the beta cumulative distribution function for the given variate and scale variables. More... |
|  | |
| template<typename T\_y , typename T\_scale\_succ , typename T\_scale\_fail > | |
| return\_type< T\_y, T\_scale\_succ,   T\_scale\_fail >::type | beta\_cdf\_log (const T\_y &y, const T\_scale\_succ &alpha, const T\_scale\_fail &beta) |
|  | |
| template<typename T\_y , typename T\_scale\_succ , typename T\_scale\_fail > | |
| return\_type< T\_y, T\_scale\_succ,   T\_scale\_fail >::type | beta\_ccdf\_log (const T\_y &y, const T\_scale\_succ &alpha, const T\_scale\_fail &beta) |
|  | |
| template<class RNG > | |
| double | beta\_rng (const double alpha, const double beta, RNG &rng) |
|  | |
| template<bool propto, typename T\_y , typename T\_loc , typename T\_scale > | |
| return\_type< T\_y, T\_loc,   T\_scale >::type | cauchy\_log (const T\_y &y, const T\_loc &mu, const T\_scale &sigma) |
|  | The log of the Cauchy density for the specified scalar(s) given the specified location parameter(s) and scale parameter(s). More... |
|  | |
| template<typename T\_y , typename T\_loc , typename T\_scale > | |
| return\_type< T\_y, T\_loc,   T\_scale >::type | cauchy\_log (const T\_y &y, const T\_loc &mu, const T\_scale &sigma) |
|  | |
| template<typename T\_y , typename T\_loc , typename T\_scale > | |
| return\_type< T\_y, T\_loc,   T\_scale >::type | cauchy\_cdf (const T\_y &y, const T\_loc &mu, const T\_scale &sigma) |
|  | Calculates the cauchy cumulative distribution function for the given variate, location, and scale. More... |
|  | |
| template<typename T\_y , typename T\_loc , typename T\_scale > | |
| return\_type< T\_y, T\_loc,   T\_scale >::type | cauchy\_cdf\_log (const T\_y &y, const T\_loc &mu, const T\_scale &sigma) |
|  | |
| template<typename T\_y , typename T\_loc , typename T\_scale > | |
| return\_type< T\_y, T\_loc,   T\_scale >::type | cauchy\_ccdf\_log (const T\_y &y, const T\_loc &mu, const T\_scale &sigma) |
|  | |
| template<class RNG > | |
| double | cauchy\_rng (const double mu, const double sigma, RNG &rng) |
|  | |
| template<bool propto, typename T\_y , typename T\_dof > | |
| return\_type< T\_y, T\_dof >::type | chi\_square\_log (const T\_y &y, const T\_dof &nu) |
|  | The log of a chi-squared density for y with the specified degrees of freedom parameter. More... |
|  | |
| template<typename T\_y , typename T\_dof > | |
| return\_type< T\_y, T\_dof >::type | chi\_square\_log (const T\_y &y, const T\_dof &nu) |
|  | |
| template<typename T\_y , typename T\_dof > | |
| return\_type< T\_y, T\_dof >::type | chi\_square\_cdf (const T\_y &y, const T\_dof &nu) |
|  | Calculates the chi square cumulative distribution function for the given variate and degrees of freedom. More... |
|  | |
| template<typename T\_y , typename T\_dof > | |
| return\_type< T\_y, T\_dof >::type | chi\_square\_cdf\_log (const T\_y &y, const T\_dof &nu) |
|  | |
| template<typename T\_y , typename T\_dof > | |
| return\_type< T\_y, T\_dof >::type | chi\_square\_ccdf\_log (const T\_y &y, const T\_dof &nu) |
|  | |
| template<class RNG > | |
| double | chi\_square\_rng (const double nu, RNG &rng) |
|  | |
| template<bool propto, typename T\_y , typename T\_loc , typename T\_scale > | |
| return\_type< T\_y, T\_loc,   T\_scale >::type | double\_exponential\_log (const T\_y &y, const T\_loc &mu, const T\_scale &sigma) |
|  | |
| template<typename T\_y , typename T\_loc , typename T\_scale > | |
| return\_type< T\_y, T\_loc,   T\_scale >::type | double\_exponential\_log (const T\_y &y, const T\_loc &mu, const T\_scale &sigma) |
|  | |
| template<typename T\_y , typename T\_loc , typename T\_scale > | |
| return\_type< T\_y, T\_loc,   T\_scale >::type | double\_exponential\_cdf (const T\_y &y, const T\_loc &mu, const T\_scale &sigma) |
|  | Calculates the double exponential cumulative density function. More... |
|  | |
| template<typename T\_y , typename T\_loc , typename T\_scale > | |
| return\_type< T\_y, T\_loc,   T\_scale >::type | double\_exponential\_cdf\_log (const T\_y &y, const T\_loc &mu, const T\_scale &sigma) |
|  | |
| template<typename T\_y , typename T\_loc , typename T\_scale > | |
| return\_type< T\_y, T\_loc,   T\_scale >::type | double\_exponential\_ccdf\_log (const T\_y &y, const T\_loc &mu, const T\_scale &sigma) |
|  | |
| template<class RNG > | |
| double | double\_exponential\_rng (const double mu, const double sigma, RNG &rng) |
|  | |
| template<bool propto, typename T\_y , typename T\_loc , typename T\_scale , typename T\_inv\_scale > | |
| return\_type< T\_y, T\_loc,   T\_scale, T\_inv\_scale >::type | exp\_mod\_normal\_log (const T\_y &y, const T\_loc &mu, const T\_scale &sigma, const T\_inv\_scale &lambda) |
|  | |
| template<typename T\_y , typename T\_loc , typename T\_scale , typename T\_inv\_scale > | |
| return\_type< T\_y, T\_loc,   T\_scale, T\_inv\_scale >::type | exp\_mod\_normal\_log (const T\_y &y, const T\_loc &mu, const T\_scale &sigma, const T\_inv\_scale &lambda) |
|  | |
| template<typename T\_y , typename T\_loc , typename T\_scale , typename T\_inv\_scale > | |
| return\_type< T\_y, T\_loc,   T\_scale, T\_inv\_scale >::type | exp\_mod\_normal\_cdf (const T\_y &y, const T\_loc &mu, const T\_scale &sigma, const T\_inv\_scale &lambda) |
|  | |
| template<typename T\_y , typename T\_loc , typename T\_scale , typename T\_inv\_scale > | |
| return\_type< T\_y, T\_loc,   T\_scale, T\_inv\_scale >::type | exp\_mod\_normal\_cdf\_log (const T\_y &y, const T\_loc &mu, const T\_scale &sigma, const T\_inv\_scale &lambda) |
|  | |
| template<typename T\_y , typename T\_loc , typename T\_scale , typename T\_inv\_scale > | |
| return\_type< T\_y, T\_loc,   T\_scale, T\_inv\_scale >::type | exp\_mod\_normal\_ccdf\_log (const T\_y &y, const T\_loc &mu, const T\_scale &sigma, const T\_inv\_scale &lambda) |
|  | |
| template<class RNG > | |
| double | exp\_mod\_normal\_rng (const double mu, const double sigma, const double lambda, RNG &rng) |
|  | |
| template<bool propto, typename T\_y , typename T\_inv\_scale > | |
| return\_type< T\_y, T\_inv\_scale >  ::type | exponential\_log (const T\_y &y, const T\_inv\_scale &beta) |
|  | The log of an exponential density for y with the specified inverse scale parameter. More... |
|  | |
| template<typename T\_y , typename T\_inv\_scale > | |
| return\_type< T\_y, T\_inv\_scale >  ::type | exponential\_log (const T\_y &y, const T\_inv\_scale &beta) |
|  | |
| template<typename T\_y , typename T\_inv\_scale > | |
| return\_type< T\_y, T\_inv\_scale >  ::type | exponential\_cdf (const T\_y &y, const T\_inv\_scale &beta) |
|  | Calculates the exponential cumulative distribution function for the given y and beta. More... |
|  | |
| template<typename T\_y , typename T\_inv\_scale > | |
| return\_type< T\_y, T\_inv\_scale >  ::type | exponential\_cdf\_log (const T\_y &y, const T\_inv\_scale &beta) |
|  | |
| template<typename T\_y , typename T\_inv\_scale > | |
| return\_type< T\_y, T\_inv\_scale >  ::type | exponential\_ccdf\_log (const T\_y &y, const T\_inv\_scale &beta) |
|  | |
| template<class RNG > | |
| double | exponential\_rng (const double beta, RNG &rng) |
|  | |
| template<bool propto, typename T\_y , typename T\_shape , typename T\_scale > | |
| return\_type< T\_y, T\_shape,   T\_scale >::type | frechet\_log (const T\_y &y, const T\_shape &alpha, const T\_scale &sigma) |
|  | |
| template<typename T\_y , typename T\_shape , typename T\_scale > | |
| return\_type< T\_y, T\_shape,   T\_scale >::type | frechet\_log (const T\_y &y, const T\_shape &alpha, const T\_scale &sigma) |
|  | |
| template<typename T\_y , typename T\_shape , typename T\_scale > | |
| return\_type< T\_y, T\_shape,   T\_scale >::type | frechet\_cdf (const T\_y &y, const T\_shape &alpha, const T\_scale &sigma) |
|  | |
| template<typename T\_y , typename T\_shape , typename T\_scale > | |
| return\_type< T\_y, T\_shape,   T\_scale >::type | frechet\_cdf\_log (const T\_y &y, const T\_shape &alpha, const T\_scale &sigma) |
|  | |
| template<typename T\_y , typename T\_shape , typename T\_scale > | |
| return\_type< T\_y, T\_shape,   T\_scale >::type | frechet\_ccdf\_log (const T\_y &y, const T\_shape &alpha, const T\_scale &sigma) |
|  | |
| template<class RNG > | |
| double | frechet\_rng (const double alpha, const double sigma, RNG &rng) |
|  | |
| template<bool propto, typename T\_y , typename T\_shape , typename T\_inv\_scale > | |
| return\_type< T\_y, T\_shape,   T\_inv\_scale >::type | gamma\_log (const T\_y &y, const T\_shape &alpha, const T\_inv\_scale &beta) |
|  | The log of a gamma density for y with the specified shape and inverse scale parameters. More... |
|  | |
| template<typename T\_y , typename T\_shape , typename T\_inv\_scale > | |
| return\_type< T\_y, T\_shape,   T\_inv\_scale >::type | gamma\_log (const T\_y &y, const T\_shape &alpha, const T\_inv\_scale &beta) |
|  | |
| template<typename T\_y , typename T\_shape , typename T\_inv\_scale > | |
| return\_type< T\_y, T\_shape,   T\_inv\_scale >::type | gamma\_cdf (const T\_y &y, const T\_shape &alpha, const T\_inv\_scale &beta) |
|  | The cumulative density function for a gamma distribution for y with the specified shape and inverse scale parameters. More... |
|  | |
| template<typename T\_y , typename T\_shape , typename T\_inv\_scale > | |
| return\_type< T\_y, T\_shape,   T\_inv\_scale >::type | gamma\_cdf\_log (const T\_y &y, const T\_shape &alpha, const T\_inv\_scale &beta) |
|  | |
| template<typename T\_y , typename T\_shape , typename T\_inv\_scale > | |
| return\_type< T\_y, T\_shape,   T\_inv\_scale >::type | gamma\_ccdf\_log (const T\_y &y, const T\_shape &alpha, const T\_inv\_scale &beta) |
|  | |
| template<class RNG > | |
| double | gamma\_rng (const double alpha, const double beta, RNG &rng) |
|  | |
| template<bool propto, typename T\_y , typename T\_loc , typename T\_scale > | |
| return\_type< T\_y, T\_loc,   T\_scale >::type | gumbel\_log (const T\_y &y, const T\_loc &mu, const T\_scale &beta) |
|  | |
| template<typename T\_y , typename T\_loc , typename T\_scale > | |
| return\_type< T\_y, T\_loc,   T\_scale >::type | gumbel\_log (const T\_y &y, const T\_loc &mu, const T\_scale &beta) |
|  | |
| template<typename T\_y , typename T\_loc , typename T\_scale > | |
| return\_type< T\_y, T\_loc,   T\_scale >::type | gumbel\_cdf (const T\_y &y, const T\_loc &mu, const T\_scale &beta) |
|  | |
| template<typename T\_y , typename T\_loc , typename T\_scale > | |
| return\_type< T\_y, T\_loc,   T\_scale >::type | gumbel\_cdf\_log (const T\_y &y, const T\_loc &mu, const T\_scale &beta) |
|  | |
| template<typename T\_y , typename T\_loc , typename T\_scale > | |
| return\_type< T\_y, T\_loc,   T\_scale >::type | gumbel\_ccdf\_log (const T\_y &y, const T\_loc &mu, const T\_scale &beta) |
|  | |
| template<class RNG > | |
| double | gumbel\_rng (const double mu, const double beta, RNG &rng) |
|  | |
| template<bool propto, typename T\_y , typename T\_dof > | |
| return\_type< T\_y, T\_dof >::type | inv\_chi\_square\_log (const T\_y &y, const T\_dof &nu) |
|  | The log of an inverse chi-squared density for y with the specified degrees of freedom parameter. More... |
|  | |
| template<typename T\_y , typename T\_dof > | |
| return\_type< T\_y, T\_dof >::type | inv\_chi\_square\_log (const T\_y &y, const T\_dof &nu) |
|  | |
| template<typename T\_y , typename T\_dof > | |
| return\_type< T\_y, T\_dof >::type | inv\_chi\_square\_cdf (const T\_y &y, const T\_dof &nu) |
|  | |
| template<typename T\_y , typename T\_dof > | |
| return\_type< T\_y, T\_dof >::type | inv\_chi\_square\_cdf\_log (const T\_y &y, const T\_dof &nu) |
|  | |
| template<typename T\_y , typename T\_dof > | |
| return\_type< T\_y, T\_dof >::type | inv\_chi\_square\_ccdf\_log (const T\_y &y, const T\_dof &nu) |
|  | |
| template<class RNG > | |
| double | inv\_chi\_square\_rng (const double nu, RNG &rng) |
|  | |
| template<bool propto, typename T\_y , typename T\_shape , typename T\_scale > | |
| return\_type< T\_y, T\_shape,   T\_scale >::type | inv\_gamma\_log (const T\_y &y, const T\_shape &alpha, const T\_scale &beta) |
|  | The log of an inverse gamma density for y with the specified shape and scale parameters. More... |
|  | |
| template<typename T\_y , typename T\_shape , typename T\_scale > | |
| return\_type< T\_y, T\_shape,   T\_scale >::type | inv\_gamma\_log (const T\_y &y, const T\_shape &alpha, const T\_scale &beta) |
|  | |
| template<typename T\_y , typename T\_shape , typename T\_scale > | |
| return\_type< T\_y, T\_shape,   T\_scale >::type | inv\_gamma\_cdf (const T\_y &y, const T\_shape &alpha, const T\_scale &beta) |
|  | The CDF of an inverse gamma density for y with the specified shape and scale parameters. More... |
|  | |
| template<typename T\_y , typename T\_shape , typename T\_scale > | |
| return\_type< T\_y, T\_shape,   T\_scale >::type | inv\_gamma\_cdf\_log (const T\_y &y, const T\_shape &alpha, const T\_scale &beta) |
|  | |
| template<typename T\_y , typename T\_shape , typename T\_scale > | |
| return\_type< T\_y, T\_shape,   T\_scale >::type | inv\_gamma\_ccdf\_log (const T\_y &y, const T\_shape &alpha, const T\_scale &beta) |
|  | |
| template<class RNG > | |
| double | inv\_gamma\_rng (const double alpha, const double beta, RNG &rng) |
|  | |
| template<bool propto, typename T\_y , typename T\_loc , typename T\_scale > | |
| return\_type< T\_y, T\_loc,   T\_scale >::type | logistic\_log (const T\_y &y, const T\_loc &mu, const T\_scale &sigma) |
|  | |
| template<typename T\_y , typename T\_loc , typename T\_scale > | |
| return\_type< T\_y, T\_loc,   T\_scale >::type | logistic\_log (const T\_y &y, const T\_loc &mu, const T\_scale &sigma) |
|  | |
| template<typename T\_y , typename T\_loc , typename T\_scale > | |
| return\_type< T\_y, T\_loc,   T\_scale >::type | logistic\_cdf (const T\_y &y, const T\_loc &mu, const T\_scale &sigma) |
|  | |
| template<typename T\_y , typename T\_loc , typename T\_scale > | |
| return\_type< T\_y, T\_loc,   T\_scale >::type | logistic\_cdf\_log (const T\_y &y, const T\_loc &mu, const T\_scale &sigma) |
|  | |
| template<typename T\_y , typename T\_loc , typename T\_scale > | |
| return\_type< T\_y, T\_loc,   T\_scale >::type | logistic\_ccdf\_log (const T\_y &y, const T\_loc &mu, const T\_scale &sigma) |
|  | |
| template<class RNG > | |
| double | logistic\_rng (const double mu, const double sigma, RNG &rng) |
|  | |
| template<bool propto, typename T\_y , typename T\_loc , typename T\_scale > | |
| return\_type< T\_y, T\_loc,   T\_scale >::type | lognormal\_log (const T\_y &y, const T\_loc &mu, const T\_scale &sigma) |
|  | |
| template<typename T\_y , typename T\_loc , typename T\_scale > | |
| return\_type< T\_y, T\_loc,   T\_scale >::type | lognormal\_log (const T\_y &y, const T\_loc &mu, const T\_scale &sigma) |
|  | |
| template<typename T\_y , typename T\_loc , typename T\_scale > | |
| return\_type< T\_y, T\_loc,   T\_scale >::type | lognormal\_cdf (const T\_y &y, const T\_loc &mu, const T\_scale &sigma) |
|  | |
| template<typename T\_y , typename T\_loc , typename T\_scale > | |
| return\_type< T\_y, T\_loc,   T\_scale >::type | lognormal\_cdf\_log (const T\_y &y, const T\_loc &mu, const T\_scale &sigma) |
|  | |
| template<typename T\_y , typename T\_loc , typename T\_scale > | |
| return\_type< T\_y, T\_loc,   T\_scale >::type | lognormal\_ccdf\_log (const T\_y &y, const T\_loc &mu, const T\_scale &sigma) |
|  | |
| template<class RNG > | |
| double | lognormal\_rng (const double mu, const double sigma, RNG &rng) |
|  | |
| template<bool propto, typename T\_y , typename T\_loc , typename T\_scale > | |
| boost::enable\_if\_c  < is\_var\_or\_arithmetic< T\_y,   T\_loc, T\_scale >::value,   typename return\_type< T\_y,   T\_loc, T\_scale >::type >::type | normal\_log (const T\_y &y, const T\_loc &mu, const T\_scale &sigma) |
|  | The log of the normal density for the specified scalar(s) given the specified mean(s) and deviation(s). More... |
|  | |
| template<typename T\_y , typename T\_loc , typename T\_scale > | |
| return\_type< T\_y, T\_loc,   T\_scale >::type | normal\_log (const T\_y &y, const T\_loc &mu, const T\_scale &sigma) |
|  | |
| template<typename T\_y , typename T\_loc , typename T\_scale > | |
| return\_type< T\_y, T\_loc,   T\_scale >::type | normal\_cdf (const T\_y &y, const T\_loc &mu, const T\_scale &sigma) |
|  | Calculates the normal cumulative distribution function for the given variate, location, and scale. More... |
|  | |
| template<typename T\_y , typename T\_loc , typename T\_scale > | |
| return\_type< T\_y, T\_loc,   T\_scale >::type | normal\_cdf\_log (const T\_y &y, const T\_loc &mu, const T\_scale &sigma) |
|  | |
| template<typename T\_y , typename T\_loc , typename T\_scale > | |
| return\_type< T\_y, T\_loc,   T\_scale >::type | normal\_ccdf\_log (const T\_y &y, const T\_loc &mu, const T\_scale &sigma) |
|  | |
| template<class RNG > | |
| double | normal\_rng (const double mu, const double sigma, RNG &rng) |
|  | |
| template<bool propto, typename T\_y , typename T\_scale , typename T\_shape > | |
| return\_type< T\_y, T\_scale,   T\_shape >::type | pareto\_log (const T\_y &y, const T\_scale &y\_min, const T\_shape &alpha) |
|  | |
| template<typename T\_y , typename T\_scale , typename T\_shape > | |
| return\_type< T\_y, T\_scale,   T\_shape >::type | pareto\_log (const T\_y &y, const T\_scale &y\_min, const T\_shape &alpha) |
|  | |
| template<typename T\_y , typename T\_scale , typename T\_shape > | |
| return\_type< T\_y, T\_scale,   T\_shape >::type | pareto\_cdf (const T\_y &y, const T\_scale &y\_min, const T\_shape &alpha) |
|  | |
| template<typename T\_y , typename T\_scale , typename T\_shape > | |
| return\_type< T\_y, T\_scale,   T\_shape >::type | pareto\_cdf\_log (const T\_y &y, const T\_scale &y\_min, const T\_shape &alpha) |
|  | |
| template<typename T\_y , typename T\_scale , typename T\_shape > | |
| return\_type< T\_y, T\_scale,   T\_shape >::type | pareto\_ccdf\_log (const T\_y &y, const T\_scale &y\_min, const T\_shape &alpha) |
|  | |
| template<class RNG > | |
| double | pareto\_rng (const double y\_min, const double alpha, RNG &rng) |
|  | |
| template<bool propto, typename T\_y , typename T\_loc , typename T\_scale , typename T\_shape > | |
| return\_type< T\_y, T\_loc,   T\_scale, T\_shape >::type | pareto\_type\_2\_log (const T\_y &y, const T\_loc &mu, const T\_scale &lambda, const T\_shape &alpha) |
|  | |
| template<typename T\_y , typename T\_loc , typename T\_scale , typename T\_shape > | |
| return\_type< T\_y, T\_loc,   T\_scale, T\_shape >::type | pareto\_type\_2\_log (const T\_y &y, const T\_loc &mu, const T\_scale &lambda, const T\_shape &alpha) |
|  | |
| template<typename T\_y , typename T\_loc , typename T\_scale , typename T\_shape > | |
| return\_type< T\_y, T\_loc,   T\_scale, T\_shape >::type | pareto\_type\_2\_cdf (const T\_y &y, const T\_loc &mu, const T\_scale &lambda, const T\_shape &alpha) |
|  | |
| template<typename T\_y , typename T\_loc , typename T\_scale , typename T\_shape > | |
| return\_type< T\_y, T\_loc,   T\_scale, T\_shape >::type | pareto\_type\_2\_cdf\_log (const T\_y &y, const T\_loc &mu, const T\_scale &lambda, const T\_shape &alpha) |
|  | |
| template<typename T\_y , typename T\_loc , typename T\_scale , typename T\_shape > | |
| return\_type< T\_y, T\_loc,   T\_scale, T\_shape >::type | pareto\_type\_2\_ccdf\_log (const T\_y &y, const T\_loc &mu, const T\_scale &lambda, const T\_shape &alpha) |
|  | |
| template<class RNG > | |
| double | pareto\_type\_2\_rng (const double mu, const double lambda, const double alpha, RNG &rng) |
|  | |
| template<bool propto, typename T\_y , typename T\_scale > | |
| return\_type< T\_y, T\_scale >::type | rayleigh\_log (const T\_y &y, const T\_scale &sigma) |
|  | |
| template<typename T\_y , typename T\_scale > | |
| return\_type< T\_y, T\_scale >::type | rayleigh\_log (const T\_y &y, const T\_scale &sigma) |
|  | |
| template<typename T\_y , typename T\_scale > | |
| return\_type< T\_y, T\_scale >::type | rayleigh\_cdf (const T\_y &y, const T\_scale &sigma) |
|  | |
| template<typename T\_y , typename T\_scale > | |
| return\_type< T\_y, T\_scale >::type | rayleigh\_cdf\_log (const T\_y &y, const T\_scale &sigma) |
|  | |
| template<typename T\_y , typename T\_scale > | |
| return\_type< T\_y, T\_scale >::type | rayleigh\_ccdf\_log (const T\_y &y, const T\_scale &sigma) |
|  | |
| template<class RNG > | |
| double | rayleigh\_rng (const double sigma, RNG &rng) |
|  | |
| template<bool propto, typename T\_y , typename T\_dof , typename T\_scale > | |
| return\_type< T\_y, T\_dof,   T\_scale >::type | scaled\_inv\_chi\_square\_log (const T\_y &y, const T\_dof &nu, const T\_scale &s) |
|  | The log of a scaled inverse chi-squared density for y with the specified degrees of freedom parameter and scale parameter. More... |
|  | |
| template<typename T\_y , typename T\_dof , typename T\_scale > | |
| return\_type< T\_y, T\_dof,   T\_scale >::type | scaled\_inv\_chi\_square\_log (const T\_y &y, const T\_dof &nu, const T\_scale &s) |
|  | |
| template<typename T\_y , typename T\_dof , typename T\_scale > | |
| return\_type< T\_y, T\_dof,   T\_scale >::type | scaled\_inv\_chi\_square\_cdf (const T\_y &y, const T\_dof &nu, const T\_scale &s) |
|  | The CDF of a scaled inverse chi-squared density for y with the specified degrees of freedom parameter and scale parameter. More... |
|  | |
| template<typename T\_y , typename T\_dof , typename T\_scale > | |
| return\_type< T\_y, T\_dof,   T\_scale >::type | scaled\_inv\_chi\_square\_cdf\_log (const T\_y &y, const T\_dof &nu, const T\_scale &s) |
|  | |
| template<typename T\_y , typename T\_dof , typename T\_scale > | |
| return\_type< T\_y, T\_dof,   T\_scale >::type | scaled\_inv\_chi\_square\_ccdf\_log (const T\_y &y, const T\_dof &nu, const T\_scale &s) |
|  | |
| template<class RNG > | |
| double | scaled\_inv\_chi\_square\_rng (const double nu, const double s, RNG &rng) |
|  | |
| template<bool propto, typename T\_y , typename T\_loc , typename T\_scale , typename T\_shape > | |
| return\_type< T\_y, T\_loc,   T\_scale, T\_shape >::type | skew\_normal\_log (const T\_y &y, const T\_loc &mu, const T\_scale &sigma, const T\_shape &alpha) |
|  | |
| template<typename T\_y , typename T\_loc , typename T\_scale , typename T\_shape > | |
| return\_type< T\_y, T\_loc,   T\_scale, T\_shape >::type | skew\_normal\_log (const T\_y &y, const T\_loc &mu, const T\_scale &sigma, const T\_shape &alpha) |
|  | |
| template<typename T\_y , typename T\_loc , typename T\_scale , typename T\_shape > | |
| return\_type< T\_y, T\_loc,   T\_scale, T\_shape >::type | skew\_normal\_cdf (const T\_y &y, const T\_loc &mu, const T\_scale &sigma, const T\_shape &alpha) |
|  | |
| template<typename T\_y , typename T\_loc , typename T\_scale , typename T\_shape > | |
| return\_type< T\_y, T\_loc,   T\_scale, T\_shape >::type | skew\_normal\_cdf\_log (const T\_y &y, const T\_loc &mu, const T\_scale &sigma, const T\_shape &alpha) |
|  | |
| template<typename T\_y , typename T\_loc , typename T\_scale , typename T\_shape > | |
| return\_type< T\_y, T\_loc,   T\_scale, T\_shape >::type | skew\_normal\_ccdf\_log (const T\_y &y, const T\_loc &mu, const T\_scale &sigma, const T\_shape &alpha) |
|  | |
| template<class RNG > | |
| double | skew\_normal\_rng (const double mu, const double sigma, const double alpha, RNG &rng) |
|  | |
| template<bool propto, typename T\_y , typename T\_dof , typename T\_loc , typename T\_scale > | |
| return\_type< T\_y, T\_dof, T\_loc,   T\_scale >::type | student\_t\_log (const T\_y &y, const T\_dof &nu, const T\_loc &mu, const T\_scale &sigma) |
|  | The log of the Student-t density for the given y, nu, mean, and scale parameter. More... |
|  | |
| template<typename T\_y , typename T\_dof , typename T\_loc , typename T\_scale > | |
| return\_type< T\_y, T\_dof, T\_loc,   T\_scale >::type | student\_t\_log (const T\_y &y, const T\_dof &nu, const T\_loc &mu, const T\_scale &sigma) |
|  | |
| template<typename T\_y , typename T\_dof , typename T\_loc , typename T\_scale > | |
| return\_type< T\_y, T\_dof, T\_loc,   T\_scale >::type | student\_t\_cdf (const T\_y &y, const T\_dof &nu, const T\_loc &mu, const T\_scale &sigma) |
|  | |
| template<typename T\_y , typename T\_dof , typename T\_loc , typename T\_scale > | |
| return\_type< T\_y, T\_dof, T\_loc,   T\_scale >::type | student\_t\_cdf\_log (const T\_y &y, const T\_dof &nu, const T\_loc &mu, const T\_scale &sigma) |
|  | |
| template<typename T\_y , typename T\_dof , typename T\_loc , typename T\_scale > | |
| return\_type< T\_y, T\_dof, T\_loc,   T\_scale >::type | student\_t\_ccdf\_log (const T\_y &y, const T\_dof &nu, const T\_loc &mu, const T\_scale &sigma) |
|  | |
| template<class RNG > | |
| double | student\_t\_rng (const double nu, const double mu, const double sigma, RNG &rng) |
|  | |
| template<bool propto, typename T\_y , typename T\_low , typename T\_high > | |
| return\_type< T\_y, T\_low,   T\_high >::type | uniform\_log (const T\_y &y, const T\_low &alpha, const T\_high &beta) |
|  | The log of a uniform density for the given y, lower, and upper bound. More... |
|  | |
| template<typename T\_y , typename T\_low , typename T\_high > | |
| return\_type< T\_y, T\_low,   T\_high >::type | uniform\_log (const T\_y &y, const T\_low &alpha, const T\_high &beta) |
|  | |
| template<typename T\_y , typename T\_low , typename T\_high > | |
| return\_type< T\_y, T\_low,   T\_high >::type | uniform\_cdf (const T\_y &y, const T\_low &alpha, const T\_high &beta) |
|  | |
| template<typename T\_y , typename T\_low , typename T\_high > | |
| return\_type< T\_y, T\_low,   T\_high >::type | uniform\_cdf\_log (const T\_y &y, const T\_low &alpha, const T\_high &beta) |
|  | |
| template<typename T\_y , typename T\_low , typename T\_high > | |
| return\_type< T\_y, T\_low,   T\_high >::type | uniform\_ccdf\_log (const T\_y &y, const T\_low &alpha, const T\_high &beta) |
|  | |
| template<class RNG > | |
| double | uniform\_rng (const double alpha, const double beta, RNG &rng) |
|  | |
| template<bool propto, typename T\_y , typename T\_loc , typename T\_scale > | |
| return\_type< T\_y, T\_loc,   T\_scale >::type | von\_mises\_log (T\_y const &y, T\_loc const &mu, T\_scale const &kappa) |
|  | |
| template<typename T\_y , typename T\_loc , typename T\_scale > | |
| return\_type< T\_y, T\_loc,   T\_scale >::type | von\_mises\_log (T\_y const &y, T\_loc const &mu, T\_scale const &kappa) |
|  | |
| template<class RNG > | |
| double | von\_mises\_rng (const double mu, const double kappa, RNG &rng) |
|  | |
| template<bool propto, typename T\_y , typename T\_shape , typename T\_scale > | |
| return\_type< T\_y, T\_shape,   T\_scale >::type | weibull\_log (const T\_y &y, const T\_shape &alpha, const T\_scale &sigma) |
|  | |
| template<typename T\_y , typename T\_shape , typename T\_scale > | |
| return\_type< T\_y, T\_shape,   T\_scale >::type | weibull\_log (const T\_y &y, const T\_shape &alpha, const T\_scale &sigma) |
|  | |
| template<typename T\_y , typename T\_shape , typename T\_scale > | |
| return\_type< T\_y, T\_shape,   T\_scale >::type | weibull\_cdf (const T\_y &y, const T\_shape &alpha, const T\_scale &sigma) |
|  | |
| template<typename T\_y , typename T\_shape , typename T\_scale > | |
| return\_type< T\_y, T\_shape,   T\_scale >::type | weibull\_cdf\_log (const T\_y &y, const T\_shape &alpha, const T\_scale &sigma) |
|  | |
| template<typename T\_y , typename T\_shape , typename T\_scale > | |
| return\_type< T\_y, T\_shape,   T\_scale >::type | weibull\_ccdf\_log (const T\_y &y, const T\_shape &alpha, const T\_scale &sigma) |
|  | |
| template<class RNG > | |
| double | weibull\_rng (const double alpha, const double sigma, RNG &rng) |
|  | |
| template<bool propto, typename T\_n , typename T\_prob > | |
| return\_type< T\_prob >::type | bernoulli\_log (const T\_n &n, const T\_prob &theta) |
|  | |
| template<typename T\_y , typename T\_prob > | |
| return\_type< T\_prob >::type | bernoulli\_log (const T\_y &n, const T\_prob &theta) |
|  | |
| template<bool propto, typename T\_n , typename T\_prob > | |
| return\_type< T\_prob >::type | bernoulli\_logit\_log (const T\_n &n, const T\_prob &theta) |
|  | |
| template<typename T\_n , typename T\_prob > | |
| return\_type< T\_prob >::type | bernoulli\_logit\_log (const T\_n &n, const T\_prob &theta) |
|  | |
| template<typename T\_n , typename T\_prob > | |
| return\_type< T\_prob >::type | bernoulli\_cdf (const T\_n &n, const T\_prob &theta) |
|  | |
| template<typename T\_n , typename T\_prob > | |
| return\_type< T\_prob >::type | bernoulli\_cdf\_log (const T\_n &n, const T\_prob &theta) |
|  | |
| template<typename T\_n , typename T\_prob > | |
| return\_type< T\_prob >::type | bernoulli\_ccdf\_log (const T\_n &n, const T\_prob &theta) |
|  | |
| template<class RNG > | |
| int | bernoulli\_rng (const double theta, RNG &rng) |
|  | |
| template<bool propto, typename T\_n , typename T\_N , typename T\_size1 , typename T\_size2 > | |
| return\_type< T\_size1, T\_size2 >  ::type | beta\_binomial\_log (const T\_n &n, const T\_N &N, const T\_size1 &alpha, const T\_size2 &beta) |
|  | |
| template<typename T\_n , typename T\_N , typename T\_size1 , typename T\_size2 > | |
| return\_type< T\_size1, T\_size2 >  ::type | beta\_binomial\_log (const T\_n &n, const T\_N &N, const T\_size1 &alpha, const T\_size2 &beta) |
|  | |
| template<typename T\_n , typename T\_N , typename T\_size1 , typename T\_size2 > | |
| return\_type< T\_size1, T\_size2 >  ::type | beta\_binomial\_cdf (const T\_n &n, const T\_N &N, const T\_size1 &alpha, const T\_size2 &beta) |
|  | |
| template<typename T\_n , typename T\_N , typename T\_size1 , typename T\_size2 > | |
| return\_type< T\_size1, T\_size2 >  ::type | beta\_binomial\_cdf\_log (const T\_n &n, const T\_N &N, const T\_size1 &alpha, const T\_size2 &beta) |
|  | |
| template<typename T\_n , typename T\_N , typename T\_size1 , typename T\_size2 > | |
| return\_type< T\_size1, T\_size2 >  ::type | beta\_binomial\_ccdf\_log (const T\_n &n, const T\_N &N, const T\_size1 &alpha, const T\_size2 &beta) |
|  | |
| template<class RNG > | |
| int | beta\_binomial\_rng (const int N, const double alpha, const double beta, RNG &rng) |
|  | |
| template<bool propto, typename T\_n , typename T\_N , typename T\_prob > | |
| return\_type< T\_prob >::type | binomial\_log (const T\_n &n, const T\_N &N, const T\_prob &theta) |
|  | |
| template<typename T\_n , typename T\_N , typename T\_prob > | |
| return\_type< T\_prob >::type | binomial\_log (const T\_n &n, const T\_N &N, const T\_prob &theta) |
|  | |
| template<bool propto, typename T\_n , typename T\_N , typename T\_prob > | |
| return\_type< T\_prob >::type | binomial\_logit\_log (const T\_n &n, const T\_N &N, const T\_prob &alpha) |
|  | |
| template<typename T\_n , typename T\_N , typename T\_prob > | |
| return\_type< T\_prob >::type | binomial\_logit\_log (const T\_n &n, const T\_N &N, const T\_prob &alpha) |
|  | |
| template<typename T\_n , typename T\_N , typename T\_prob > | |
| return\_type< T\_prob >::type | binomial\_cdf (const T\_n &n, const T\_N &N, const T\_prob &theta) |
|  | |
| template<typename T\_n , typename T\_N , typename T\_prob > | |
| return\_type< T\_prob >::type | binomial\_cdf\_log (const T\_n &n, const T\_N &N, const T\_prob &theta) |
|  | |
| template<typename T\_n , typename T\_N , typename T\_prob > | |
| return\_type< T\_prob >::type | binomial\_ccdf\_log (const T\_n &n, const T\_N &N, const T\_prob &theta) |
|  | |
| template<class RNG > | |
| int | binomial\_rng (const int N, const double theta, RNG &rng) |
|  | |
| template<bool propto, typename T\_n , typename T\_N , typename T\_a , typename T\_b > | |
| double | hypergeometric\_log (const T\_n &n, const T\_N &N, const T\_a &a, const T\_b &b) |
|  | |
| template<typename T\_n , typename T\_N , typename T\_a , typename T\_b > | |
| double | hypergeometric\_log (const T\_n &n, const T\_N &N, const T\_a &a, const T\_b &b) |
|  | |
| template<class RNG > | |
| int | hypergeometric\_rng (const int N, const int a, const int b, RNG &rng) |
|  | |
| template<bool propto, typename T\_n , typename T\_shape , typename T\_inv\_scale > | |
| return\_type< T\_shape,   T\_inv\_scale >::type | neg\_binomial\_log (const T\_n &n, const T\_shape &alpha, const T\_inv\_scale &beta) |
|  | |
| template<typename T\_n , typename T\_shape , typename T\_inv\_scale > | |
| return\_type< T\_shape,   T\_inv\_scale >::type | neg\_binomial\_log (const T\_n &n, const T\_shape &alpha, const T\_inv\_scale &beta) |
|  | |
| template<typename T\_n , typename T\_shape , typename T\_inv\_scale > | |
| return\_type< T\_shape,   T\_inv\_scale >::type | neg\_binomial\_cdf (const T\_n &n, const T\_shape &alpha, const T\_inv\_scale &beta) |
|  | |
| template<typename T\_n , typename T\_shape , typename T\_inv\_scale > | |
| return\_type< T\_shape,   T\_inv\_scale >::type | neg\_binomial\_cdf\_log (const T\_n &n, const T\_shape &alpha, const T\_inv\_scale &beta) |
|  | |
| template<typename T\_n , typename T\_shape , typename T\_inv\_scale > | |
| return\_type< T\_shape,   T\_inv\_scale >::type | neg\_binomial\_ccdf\_log (const T\_n &n, const T\_shape &alpha, const T\_inv\_scale &beta) |
|  | |
| template<class RNG > | |
| int | neg\_binomial\_rng (const double alpha, const double beta, RNG &rng) |
|  | |
| template<bool propto, typename T\_n , typename T\_location , typename T\_inv\_scale > | |
| return\_type< T\_location,   T\_inv\_scale >::type | neg\_binomial\_2\_log (const T\_n &n, const T\_location &mu, const T\_inv\_scale &phi) |
|  | |
| template<typename T\_n , typename T\_location , typename T\_inv\_scale > | |
| return\_type< T\_location,   T\_inv\_scale >::type | neg\_binomial\_2\_log (const T\_n &n, const T\_location &mu, const T\_inv\_scale &phi) |
|  | |
| template<bool propto, typename T\_n , typename T\_log\_location , typename T\_inv\_scale > | |
| return\_type< T\_log\_location,   T\_inv\_scale >::type | neg\_binomial\_2\_log\_log (const T\_n &n, const T\_log\_location &eta, const T\_inv\_scale &phi) |
|  | |
| template<typename T\_n , typename T\_log\_location , typename T\_inv\_scale > | |
| return\_type< T\_log\_location,   T\_inv\_scale >::type | neg\_binomial\_2\_log\_log (const T\_n &n, const T\_log\_location &eta, const T\_inv\_scale &phi) |
|  | |
| template<class RNG > | |
| int | neg\_binomial\_2\_rng (const double mu, const double phi, RNG &rng) |
|  | |
| template<class RNG > | |
| int | neg\_binomial\_2\_log\_rng (const double eta, const double phi, RNG &rng) |
|  | |
| template<typename T > | |
| T | log\_inv\_logit\_diff (const T &alpha, const T &beta) |
|  | |
| template<bool propto, typename T\_lambda , typename T\_cut > | |
| boost::math::tools::promote\_args  < T\_lambda, T\_cut >::type | ordered\_logistic\_log (int y, const T\_lambda &lambda, const Eigen::Matrix< T\_cut, Eigen::Dynamic, 1 > &c) |
|  | Returns the (natural) log probability of the specified integer outcome given the continuous location and specified cutpoints in an ordered logistic model. More... |
|  | |
| template<typename T\_lambda , typename T\_cut > | |
| boost::math::tools::promote\_args  < T\_lambda, T\_cut >::type | ordered\_logistic\_log (int y, const T\_lambda &lambda, const Eigen::Matrix< T\_cut, Eigen::Dynamic, 1 > &c) |
|  | |
| template<class RNG > | |
| int | ordered\_logistic\_rng (const double eta, const Eigen::Matrix< double, Eigen::Dynamic, 1 > &c, RNG &rng) |
|  | |
| template<bool propto, typename T\_n , typename T\_rate > | |
| return\_type< T\_rate >::type | poisson\_log (const T\_n &n, const T\_rate &lambda) |
|  | |
| template<typename T\_n , typename T\_rate > | |
| return\_type< T\_rate >::type | poisson\_log (const T\_n &n, const T\_rate &lambda) |
|  | |
| template<bool propto, typename T\_n , typename T\_log\_rate > | |
| return\_type< T\_log\_rate >::type | poisson\_log\_log (const T\_n &n, const T\_log\_rate &alpha) |
|  | |
| template<typename T\_n , typename T\_log\_rate > | |
| return\_type< T\_log\_rate >::type | poisson\_log\_log (const T\_n &n, const T\_log\_rate &alpha) |
|  | |
| template<typename T\_n , typename T\_rate > | |
| return\_type< T\_rate >::type | poisson\_cdf (const T\_n &n, const T\_rate &lambda) |
|  | |
| template<typename T\_n , typename T\_rate > | |
| return\_type< T\_rate >::type | poisson\_cdf\_log (const T\_n &n, const T\_rate &lambda) |
|  | |
| template<typename T\_n , typename T\_rate > | |
| return\_type< T\_rate >::type | poisson\_ccdf\_log (const T\_n &n, const T\_rate &lambda) |
|  | |
| template<class RNG > | |
| int | poisson\_rng (const double lambda, RNG &rng) |
|  | |
| template<typename T > | |
| void | factor\_U (const Eigen::Matrix< T, Eigen::Dynamic, Eigen::Dynamic > &U, Eigen::Array< T, Eigen::Dynamic, 1 > &CPCs) |
|  | This function is intended to make starting values, given a unit upper-triangular matrix U such that U'DU is a correlation matrix. More... |
|  | |
| template<typename T > | |
| bool | factor\_cov\_matrix (const Eigen::Matrix< T, Eigen::Dynamic, Eigen::Dynamic > &Sigma, Eigen::Array< T, Eigen::Dynamic, 1 > &CPCs, Eigen::Array< T, Eigen::Dynamic, 1 > &sds) |
|  | This function is intended to make starting values, given a covariance matrix Sigma. More... |
|  | |
| template<typename T > | |
| Eigen::Matrix< T,   Eigen::Dynamic, Eigen::Dynamic > | read\_corr\_L (const Eigen::Array< T, Eigen::Dynamic, 1 > &CPCs, const size\_t K) |
|  | Return the Cholesky factor of the correlation matrix of the specified dimensionality corresponding to the specified canonical partial correlations. More... |
|  | |
| template<typename T > | |
| Eigen::Matrix< T,   Eigen::Dynamic, Eigen::Dynamic > | read\_corr\_matrix (const Eigen::Array< T, Eigen::Dynamic, 1 > &CPCs, const size\_t K) |
|  | Return the correlation matrix of the specified dimensionality corresponding to the specified canonical partial correlations. More... |
|  | |
| template<typename T > | |
| Eigen::Matrix< T,   Eigen::Dynamic, Eigen::Dynamic > | read\_corr\_L (const Eigen::Array< T, Eigen::Dynamic, 1 > &CPCs, const size\_t K, T &log\_prob) |
|  | Return the Cholesky factor of the correlation matrix of the specified dimensionality corresponding to the specified canonical partial correlations, incrementing the specified scalar reference with the log absolute determinant of the Jacobian of the transformation. More... |
|  | |
| template<typename T > | |
| Eigen::Matrix< T,   Eigen::Dynamic, Eigen::Dynamic > | read\_corr\_matrix (const Eigen::Array< T, Eigen::Dynamic, 1 > &CPCs, const size\_t K, T &log\_prob) |
|  | Return the correlation matrix of the specified dimensionality corresponding to the specified canonical partial correlations, incrementing the specified scalar reference with the log absolute determinant of the Jacobian of the transformation. More... |
|  | |
| template<typename T > | |
| Eigen::Matrix< T,   Eigen::Dynamic, Eigen::Dynamic > | read\_cov\_L (const Eigen::Array< T, Eigen::Dynamic, 1 > &CPCs, const Eigen::Array< T, Eigen::Dynamic, 1 > &sds, T &log\_prob) |
|  | This is the function that should be called prior to evaluating the density of any elliptical distribution. More... |
|  | |
| template<typename T > | |
| Eigen::Matrix< T,   Eigen::Dynamic, Eigen::Dynamic > | read\_cov\_matrix (const Eigen::Array< T, Eigen::Dynamic, 1 > &CPCs, const Eigen::Array< T, Eigen::Dynamic, 1 > &sds, T &log\_prob) |
|  | A generally worse alternative to call prior to evaluating the density of an elliptical distribution. More... |
|  | |
| template<typename T > | |
| Eigen::Matrix< T,   Eigen::Dynamic, Eigen::Dynamic > | read\_cov\_matrix (const Eigen::Array< T, Eigen::Dynamic, 1 > &CPCs, const Eigen::Array< T, Eigen::Dynamic, 1 > &sds) |
|  | Builds a covariance matrix from CPCs and standard deviations. More... |
|  | |
| template<typename T > | |
| const Eigen::Array< T,   Eigen::Dynamic, 1 > | make\_nu (const T eta, const size\_t K) |
|  | This function calculates the degrees of freedom for the t distribution that corresponds to the shape parameter in the Lewandowski et. More... |
|  | |
| template<typename T > | |
| T | identity\_constrain (T x) |
|  | Returns the result of applying the identity constraint transform to the input. More... |
|  | |
| template<typename T > | |
| T | identity\_constrain (const T x, T &) |
|  | Returns the result of applying the identity constraint transform to the input and increments the log probability reference with the log absolute Jacobian determinant. More... |
|  | |
| template<typename T > | |
| T | identity\_free (const T y) |
|  | Returns the result of applying the inverse of the identity constraint transform to the input. More... |
|  | |
| template<typename T > | |
| T | positive\_constrain (const T x) |
|  | Return the positive value for the specified unconstrained input. More... |
|  | |
| template<typename T > | |
| T | positive\_constrain (const T x, T &lp) |
|  | Return the positive value for the specified unconstrained input, incrementing the scalar reference with the log absolute Jacobian determinant. More... |
|  | |
| template<typename T > | |
| T | positive\_free (const T y) |
|  | Return the unconstrained value corresponding to the specified positive-constrained value. More... |
|  | |
| template<typename T , typename TL > | |
| T | lb\_constrain (const T x, const TL lb) |
|  | Return the lower-bounded value for the specified unconstrained input and specified lower bound. More... |
|  | |
| template<typename T , typename TL > | |
| boost::math::tools::promote\_args  < T, TL >::type | lb\_constrain (const T x, const TL lb, T &lp) |
|  | Return the lower-bounded value for the speicifed unconstrained input and specified lower bound, incrementing the specified reference with the log absolute Jacobian determinant of the transform. More... |
|  | |
| template<typename T , typename TL > | |
| boost::math::tools::promote\_args  < T, TL >::type | lb\_free (const T y, const TL lb) |
|  | Return the unconstrained value that produces the specified lower-bound constrained value. More... |
|  | |
| template<typename T , typename TU > | |
| boost::math::tools::promote\_args  < T, TU >::type | ub\_constrain (const T x, const TU ub) |
|  | Return the upper-bounded value for the specified unconstrained scalar and upper bound. More... |
|  | |
| template<typename T , typename TU > | |
| boost::math::tools::promote\_args  < T, TU >::type | ub\_constrain (const T x, const TU ub, T &lp) |
|  | Return the upper-bounded value for the specified unconstrained scalar and upper bound and increment the specified log probability reference with the log absolute Jacobian determinant of the transform. More... |
|  | |
| template<typename T , typename TU > | |
| boost::math::tools::promote\_args  < T, TU >::type | ub\_free (const T y, const TU ub) |
|  | Return the free scalar that corresponds to the specified upper-bounded value with respect to the specified upper bound. More... |
|  | |
| template<typename T , typename TL , typename TU > | |
| boost::math::tools::promote\_args  < T, TL, TU >::type | lub\_constrain (const T x, TL lb, TU ub) |
|  | Return the lower- and upper-bounded scalar derived by transforming the specified free scalar given the specified lower and upper bounds. More... |
|  | |
| template<typename T , typename TL , typename TU > | |
| boost::math::tools::promote\_args  < T, TL, TU >::type | lub\_constrain (const T x, const TL lb, const TU ub, T &lp) |
|  | Return the lower- and upper-bounded scalar derived by transforming the specified free scalar given the specified lower and upper bounds and increment the specified log probability with the log absolute Jacobian determinant. More... |
|  | |
| template<typename T , typename TL , typename TU > | |
| boost::math::tools::promote\_args  < T, TL, TU >::type | lub\_free (const T y, TL lb, TU ub) |
|  | Return the unconstrained scalar that transforms to the specified lower- and upper-bounded scalar given the specified bounds. More... |
|  | |
| template<typename T > | |
| T | prob\_constrain (const T x) |
|  | Return a probability value constrained to fall between 0 and 1 (inclusive) for the specified free scalar. More... |
|  | |
| template<typename T > | |
| T | prob\_constrain (const T x, T &lp) |
|  | Return a probability value constrained to fall between 0 and 1 (inclusive) for the specified free scalar and increment the specified log probability reference with the log absolute Jacobian determinant of the transform. More... |
|  | |
| template<typename T > | |
| T | prob\_free (const T y) |
|  | Return the free scalar that when transformed to a probability produces the specified scalar. More... |
|  | |
| template<typename T > | |
| T | corr\_constrain (const T x) |
|  | Return the result of transforming the specified scalar to have a valid correlation value between -1 and 1 (inclusive). More... |
|  | |
| template<typename T > | |
| T | corr\_constrain (const T x, T &lp) |
|  | Return the result of transforming the specified scalar to have a valid correlation value between -1 and 1 (inclusive). More... |
|  | |
| template<typename T > | |
| T | corr\_free (const T y) |
|  | Return the unconstrained scalar that when transformed to a valid correlation produces the specified value. More... |
|  | |
| template<typename T > | |
| Eigen::Matrix< T,   Eigen::Dynamic, 1 > | unit\_vector\_constrain (const Eigen::Matrix< T, Eigen::Dynamic, 1 > &y) |
|  | Return the unit length vector corresponding to the free vector y. More... |
|  | |
| template<typename T > | |
| Eigen::Matrix< T,   Eigen::Dynamic, 1 > | unit\_vector\_constrain (const Eigen::Matrix< T, Eigen::Dynamic, 1 > &y, T &lp) |
|  | Return the unit length vector corresponding to the free vector y. More... |
|  | |
| template<typename T > | |
| Eigen::Matrix< T,   Eigen::Dynamic, 1 > | unit\_vector\_free (const Eigen::Matrix< T, Eigen::Dynamic, 1 > &x) |
|  | |
| template<typename T > | |
| Eigen::Matrix< T,   Eigen::Dynamic, 1 > | simplex\_constrain (const Eigen::Matrix< T, Eigen::Dynamic, 1 > &y) |
|  | Return the simplex corresponding to the specified free vector. More... |
|  | |
| template<typename T > | |
| Eigen::Matrix< T,   Eigen::Dynamic, 1 > | simplex\_constrain (const Eigen::Matrix< T, Eigen::Dynamic, 1 > &y, T &lp) |
|  | Return the simplex corresponding to the specified free vector and increment the specified log probability reference with the log absolute Jacobian determinant of the transform. More... |
|  | |
| template<typename T > | |
| Eigen::Matrix< T,   Eigen::Dynamic, 1 > | simplex\_free (const Eigen::Matrix< T, Eigen::Dynamic, 1 > &x) |
|  | Return an unconstrained vector that when transformed produces the specified simplex. More... |
|  | |
| template<typename T > | |
| Eigen::Matrix< T,   Eigen::Dynamic, 1 > | ordered\_constrain (const Eigen::Matrix< T, Eigen::Dynamic, 1 > &x) |
|  | Return an increasing ordered vector derived from the specified free vector. More... |
|  | |
| template<typename T > | |
| Eigen::Matrix< T,   Eigen::Dynamic, 1 > | ordered\_constrain (const Eigen::Matrix< T, Eigen::Dynamic, 1 > &x, T &lp) |
|  | Return a positive valued, increasing ordered vector derived from the specified free vector and increment the specified log probability reference with the log absolute Jacobian determinant of the transform. More... |
|  | |
| template<typename T > | |
| Eigen::Matrix< T,   Eigen::Dynamic, 1 > | ordered\_free (const Eigen::Matrix< T, Eigen::Dynamic, 1 > &y) |
|  | Return the vector of unconstrained scalars that transform to the specified positive ordered vector. More... |
|  | |
| template<typename T > | |
| Eigen::Matrix< T,   Eigen::Dynamic, 1 > | positive\_ordered\_constrain (const Eigen::Matrix< T, Eigen::Dynamic, 1 > &x) |
|  | Return an increasing positive ordered vector derived from the specified free vector. More... |
|  | |
| template<typename T > | |
| Eigen::Matrix< T,   Eigen::Dynamic, 1 > | positive\_ordered\_constrain (const Eigen::Matrix< T, Eigen::Dynamic, 1 > &x, T &lp) |
|  | Return a positive valued, increasing positive ordered vector derived from the specified free vector and increment the specified log probability reference with the log absolute Jacobian determinant of the transform. More... |
|  | |
| template<typename T > | |
| Eigen::Matrix< T,   Eigen::Dynamic, 1 > | positive\_ordered\_free (const Eigen::Matrix< T, Eigen::Dynamic, 1 > &y) |
|  | Return the vector of unconstrained scalars that transform to the specified positive ordered vector. More... |
|  | |
| template<typename T > | |
| Eigen::Matrix< T,   Eigen::Dynamic, Eigen::Dynamic > | cholesky\_factor\_constrain (const Eigen::Matrix< T, Eigen::Dynamic, 1 > &x, int M, int N) |
|  | Return the Cholesky factor of the specified size read from the specified vector. More... |
|  | |
| template<typename T > | |
| Eigen::Matrix< T,   Eigen::Dynamic, Eigen::Dynamic > | cholesky\_factor\_constrain (const Eigen::Matrix< T, Eigen::Dynamic, 1 > &x, int M, int N, T &lp) |
|  | Return the Cholesky factor of the specified size read from the specified vector and increment the specified log probability reference with the log Jacobian adjustment of the transform. More... |
|  | |
| template<typename T > | |
| Eigen::Matrix< T,   Eigen::Dynamic, 1 > | cholesky\_factor\_free (const Eigen::Matrix< T, Eigen::Dynamic, Eigen::Dynamic > &y) |
|  | Return the unconstrained vector of parameters correspdonding to the specified Cholesky factor. More... |
|  | |
| template<typename T > | |
| Eigen::Matrix< T,   Eigen::Dynamic, Eigen::Dynamic > | cholesky\_corr\_constrain (const Eigen::Matrix< T, Eigen::Dynamic, 1 > &y, int K) |
|  | |
| template<typename T > | |
| Eigen::Matrix< T,   Eigen::Dynamic, Eigen::Dynamic > | cholesky\_corr\_constrain (const Eigen::Matrix< T, Eigen::Dynamic, 1 > &y, int K, T &lp) |
|  | |
| template<typename T > | |
| Eigen::Matrix< T,   Eigen::Dynamic, 1 > | cholesky\_corr\_free (const Eigen::Matrix< T, Eigen::Dynamic, Eigen::Dynamic > &x) |
|  | |
| template<typename T > | |
| Eigen::Matrix< T,   Eigen::Dynamic, Eigen::Dynamic > | corr\_matrix\_constrain (const Eigen::Matrix< T, Eigen::Dynamic, 1 > &x, typename math::index\_type< Eigen::Matrix< T, Eigen::Dynamic, 1 > >::type k) |
|  | Return the correlation matrix of the specified dimensionality derived from the specified vector of unconstrained values. More... |
|  | |
| template<typename T > | |
| Eigen::Matrix< T,   Eigen::Dynamic, Eigen::Dynamic > | corr\_matrix\_constrain (const Eigen::Matrix< T, Eigen::Dynamic, 1 > &x, typename math::index\_type< Eigen::Matrix< T, Eigen::Dynamic, 1 > >::type k, T &lp) |
|  | Return the correlation matrix of the specified dimensionality derived from the specified vector of unconstrained values. More... |
|  | |
| template<typename T > | |
| Eigen::Matrix< T,   Eigen::Dynamic, 1 > | corr\_matrix\_free (const Eigen::Matrix< T, Eigen::Dynamic, Eigen::Dynamic > &y) |
|  | Return the vector of unconstrained partial correlations that define the specified correlation matrix when transformed. More... |
|  | |
| template<typename T > | |
| Eigen::Matrix< T,   Eigen::Dynamic, Eigen::Dynamic > | cov\_matrix\_constrain (const Eigen::Matrix< T, Eigen::Dynamic, 1 > &x, typename math::index\_type< Eigen::Matrix< T, Eigen::Dynamic, 1 > >::type K) |
|  | Return the symmetric, positive-definite matrix of dimensions K by K resulting from transforming the specified finite vector of size K plus (K choose 2). More... |
|  | |
| template<typename T > | |
| Eigen::Matrix< T,   Eigen::Dynamic, Eigen::Dynamic > | cov\_matrix\_constrain (const Eigen::Matrix< T, Eigen::Dynamic, 1 > &x, typename math::index\_type< Eigen::Matrix< T, Eigen::Dynamic, Eigen::Dynamic > >::type K, T &lp) |
|  | Return the symmetric, positive-definite matrix of dimensions K by K resulting from transforming the specified finite vector of size K plus (K choose 2). More... |
|  | |
| template<typename T > | |
| Eigen::Matrix< T,   Eigen::Dynamic, 1 > | cov\_matrix\_free (const Eigen::Matrix< T, Eigen::Dynamic, Eigen::Dynamic > &y) |
|  | The covariance matrix derived from the symmetric view of the lower-triangular view of the K by K specified matrix is freed to return a vector of size K + (K choose 2). More... |
|  | |
| template<typename T > | |
| Eigen::Matrix< T,   Eigen::Dynamic, Eigen::Dynamic > | cov\_matrix\_constrain\_lkj (const Eigen::Matrix< T, Eigen::Dynamic, 1 > &x, size\_t k) |
|  | Return the covariance matrix of the specified dimensionality derived from constraining the specified vector of unconstrained values. More... |
|  | |
| template<typename T > | |
| Eigen::Matrix< T,   Eigen::Dynamic, Eigen::Dynamic > | cov\_matrix\_constrain\_lkj (const Eigen::Matrix< T, Eigen::Dynamic, 1 > &x, size\_t k, T &lp) |
|  | Return the covariance matrix of the specified dimensionality derived from constraining the specified vector of unconstrained values and increment the specified log probability reference with the log absolute Jacobian determinant. More... |
|  | |
| template<typename T > | |
| Eigen::Matrix< T,   Eigen::Dynamic, 1 > | cov\_matrix\_free\_lkj (const Eigen::Matrix< T, Eigen::Dynamic, Eigen::Dynamic > &y) |
|  | Return the vector of unconstrained partial correlations and deviations that transform to the specified covariance matrix. More... |
|  | |

|  |  |
| --- | --- |
| Variables | |
| const double | CONSTRAINT\_TOLERANCE = 1E-8 |
|  | |

## Detailed Description

Templated probability distributions.

All paramaterizations are based on Bayesian Data Analysis. Error handling for the distributions is described in Error Handling Policies.

## Function Documentation

template<typename T >

|  |  |  |  |
| --- | --- | --- | --- |
| void stan::prob::autocorrelation | ( | const std::vector< T > & | *y*, |
|  |  | std::vector< T > & | *ac*, |
|  |  | Eigen::FFT< T > & | *fft* |
|  | ) |  |  |

Write autocorrelation estimates for every lag for the specified input sequence into the specified result using the specified FFT engine.

The return vector be resized to the same length as the input sequence with lags given by array index.

The implementation involves a fast Fourier transform, followed by a normalization, followed by an inverse transform.

An FFT engine can be created for reuse for type double with:

```
    Eigen::FFT<double> fft;
```

Template Parameters
:   |  |  |
    | --- | --- |
    | T | Scalar type. |

Parameters
:   |  |  |
    | --- | --- |
    | y | Input sequence. |
    | ac | Autocorrelations. |
    | fft | FFT engine instance. |

Definition at line 56 of file autocorrelation.hpp.

template<typename T >

|  |  |  |  |
| --- | --- | --- | --- |
| void stan::prob::autocorrelation | ( | const std::vector< T > & | *y*, |
|  |  | std::vector< T > & | *ac* |
|  | ) |  |  |

Write autocorrelation estimates for every lag for the specified input sequence into the specified result.

The return vector be resized to the same length as the input sequence with lags given by array index.

The implementation involves a fast Fourier transform, followed by a normalization, followed by an inverse transform.

This method is just a light wrapper around the three-argument autocorrelation function

Template Parameters
:   |  |  |
    | --- | --- |
    | T | Scalar type. |

Parameters
:   |  |  |
    | --- | --- |
    | y | Input sequence. |
    | ac | Autocorrelations. |

Definition at line 126 of file autocorrelation.hpp.

template<typename T >

|  |  |  |  |
| --- | --- | --- | --- |
| void stan::prob::autocovariance | ( | const std::vector< T > & | *y*, |
|  |  | std::vector< T > & | *acov*, |
|  |  | Eigen::FFT< T > & | *fft* |
|  | ) |  |  |

Write autocovariance estimates for every lag for the specified input sequence into the specified result using the specified FFT engine.

The return vector be resized to the same length as the input sequence with lags given by array index.

The implementation involves a fast Fourier transform, followed by a normalization, followed by an inverse transform.

An FFT engine can be created for reuse for type double with:

```
    Eigen::FFT<double> fft;
```

Template Parameters
:   |  |  |
    | --- | --- |
    | T | Scalar type. |

Parameters
:   |  |  |
    | --- | --- |
    | y | Input sequence. |
    | acov | Autocovariance. |
    | fft | FFT engine instance. |

Definition at line 33 of file autocovariance.hpp.

template<typename T >

|  |  |  |  |
| --- | --- | --- | --- |
| void stan::prob::autocovariance | ( | const std::vector< T > & | *y*, |
|  |  | std::vector< T > & | *acov* |
|  | ) |  |  |

Write autocovariance estimates for every lag for the specified input sequence into the specified result.

The return vector be resized to the same length as the input sequence with lags given by array index.

The implementation involves a fast Fourier transform, followed by a normalization, followed by an inverse transform.

This method is just a light wrapper around the three-argument autocovariance function

Template Parameters
:   |  |  |
    | --- | --- |
    | T | Scalar type. |

Parameters
:   |  |  |
    | --- | --- |
    | y | Input sequence. |
    | acov | Autocovariances. |

Definition at line 62 of file autocovariance.hpp.

template<typename T\_n , typename T\_prob >

|  |  |  |  |
| --- | --- | --- | --- |
| return\_type<T\_prob>::type stan::prob::bernoulli\_ccdf\_log | ( | const T\_n & | *n*, |
|  |  | const T\_prob & | *theta* |
|  | ) |  |  |

Definition at line 333 of file bernoulli.hpp.

template<typename T\_n , typename T\_prob >

|  |  |  |  |
| --- | --- | --- | --- |
| return\_type<T\_prob>::type stan::prob::bernoulli\_cdf | ( | const T\_n & | *n*, |
|  |  | const T\_prob & | *theta* |
|  | ) |  |  |

Definition at line 212 of file bernoulli.hpp.

template<typename T\_n , typename T\_prob >

|  |  |  |  |
| --- | --- | --- | --- |
| return\_type<T\_prob>::type stan::prob::bernoulli\_cdf\_log | ( | const T\_n & | *n*, |
|  |  | const T\_prob & | *theta* |
|  | ) |  |  |

Definition at line 274 of file bernoulli.hpp.

template<bool propto, typename T\_n , typename T\_prob >

|  |  |  |  |
| --- | --- | --- | --- |
| return\_type<T\_prob>::type stan::prob::bernoulli\_log | ( | const T\_n & | *n*, |
|  |  | const T\_prob & | *theta* |
|  | ) |  |  |

Definition at line 25 of file bernoulli.hpp.

template<typename T\_y , typename T\_prob >

|  |  |  |  |
| --- | --- | --- | --- |
| return\_type<T\_prob>::type stan::prob::bernoulli\_log | ( | const T\_y & | *n*, |
|  |  | const T\_prob & | *theta* |
|  | ) |  |  |

inline

Definition at line 118 of file bernoulli.hpp.

template<bool propto, typename T\_n , typename T\_prob >

|  |  |  |  |
| --- | --- | --- | --- |
| return\_type<T\_prob>::type stan::prob::bernoulli\_logit\_log | ( | const T\_n & | *n*, |
|  |  | const T\_prob & | *theta* |
|  | ) |  |  |

Definition at line 128 of file bernoulli.hpp.

template<typename T\_n , typename T\_prob >

|  |  |  |  |
| --- | --- | --- | --- |
| return\_type<T\_prob>::type stan::prob::bernoulli\_logit\_log | ( | const T\_n & | *n*, |
|  |  | const T\_prob & | *theta* |
|  | ) |  |  |

inline

Definition at line 204 of file bernoulli.hpp.

template<class RNG >

|  |  |  |  |
| --- | --- | --- | --- |
| int stan::prob::bernoulli\_rng | ( | const double | *theta*, |
|  |  | RNG & | *rng* |
|  | ) |  |  |

inline

Definition at line 394 of file bernoulli.hpp.

template<typename T\_n , typename T\_N , typename T\_size1 , typename T\_size2 >

|  |  |  |  |
| --- | --- | --- | --- |
| return\_type<T\_size1,T\_size2>::type stan::prob::beta\_binomial\_ccdf\_log | ( | const T\_n & | *n*, |
|  |  | const T\_N & | *N*, |
|  |  | const T\_size1 & | *alpha*, |
|  |  | const T\_size2 & | *beta* |
|  | ) |  |  |

Definition at line 428 of file beta\_binomial.hpp.

template<typename T\_n , typename T\_N , typename T\_size1 , typename T\_size2 >

|  |  |  |  |
| --- | --- | --- | --- |
| return\_type<T\_size1,T\_size2>::type stan::prob::beta\_binomial\_cdf | ( | const T\_n & | *n*, |
|  |  | const T\_N & | *N*, |
|  |  | const T\_size1 & | *alpha*, |
|  |  | const T\_size2 & | *beta* |
|  | ) |  |  |

Definition at line 191 of file beta\_binomial.hpp.

template<typename T\_n , typename T\_N , typename T\_size1 , typename T\_size2 >

|  |  |  |  |
| --- | --- | --- | --- |
| return\_type<T\_size1,T\_size2>::type stan::prob::beta\_binomial\_cdf\_log | ( | const T\_n & | *n*, |
|  |  | const T\_N & | *N*, |
|  |  | const T\_size1 & | *alpha*, |
|  |  | const T\_size2 & | *beta* |
|  | ) |  |  |

Definition at line 314 of file beta\_binomial.hpp.

template<bool propto, typename T\_n , typename T\_N , typename T\_size1 , typename T\_size2 >

|  |  |  |  |
| --- | --- | --- | --- |
| return\_type<T\_size1,T\_size2>::type stan::prob::beta\_binomial\_log | ( | const T\_n & | *n*, |
|  |  | const T\_N & | *N*, |
|  |  | const T\_size1 & | *alpha*, |
|  |  | const T\_size2 & | *beta* |
|  | ) |  |  |

Definition at line 28 of file beta\_binomial.hpp.

template<typename T\_n , typename T\_N , typename T\_size1 , typename T\_size2 >

|  |  |  |  |
| --- | --- | --- | --- |
| return\_type<T\_size1,T\_size2>::type stan::prob::beta\_binomial\_log | ( | const T\_n & | *n*, |
|  |  | const T\_N & | *N*, |
|  |  | const T\_size1 & | *alpha*, |
|  |  | const T\_size2 & | *beta* |
|  | ) |  |  |

Definition at line 182 of file beta\_binomial.hpp.

template<class RNG >

|  |  |  |  |
| --- | --- | --- | --- |
| int stan::prob::beta\_binomial\_rng | ( | const int | *N*, |
|  |  | const double | *alpha*, |
|  |  | const double | *beta*, |
|  |  | RNG & | *rng* |
|  | ) |  |  |

inline

Definition at line 541 of file beta\_binomial.hpp.

template<typename T\_y , typename T\_scale\_succ , typename T\_scale\_fail >

|  |  |  |  |
| --- | --- | --- | --- |
| return\_type<T\_y,T\_scale\_succ,T\_scale\_fail>::type stan::prob::beta\_ccdf\_log | ( | const T\_y & | *y*, |
|  |  | const T\_scale\_succ & | *alpha*, |
|  |  | const T\_scale\_fail & | *beta* |
|  | ) |  |  |

Definition at line 476 of file beta.hpp.

template<typename T\_y , typename T\_scale\_succ , typename T\_scale\_fail >

|  |  |  |  |
| --- | --- | --- | --- |
| return\_type<T\_y,T\_scale\_succ,T\_scale\_fail>::type stan::prob::beta\_cdf | ( | const T\_y & | *y*, |
|  |  | const T\_scale\_succ & | *alpha*, |
|  |  | const T\_scale\_fail & | *beta* |
|  | ) |  |  |

Calculates the beta cumulative distribution function for the given variate and scale variables.

Parameters
:   |  |  |
    | --- | --- |
    | y | A scalar variate. |
    | alpha | Prior sample size. |
    | beta | Prior sample size. |

Returns
:   The beta cdf evaluated at the specified arguments.

Template Parameters
:   |  |  |
    | --- | --- |
    | T\_y | Type of y. |
    | T\_scale\_succ | Type of alpha. |
    | T\_scale\_fail | Type of beta. |

Definition at line 213 of file beta.hpp.

template<typename T\_y , typename T\_scale\_succ , typename T\_scale\_fail >

|  |  |  |  |
| --- | --- | --- | --- |
| return\_type<T\_y,T\_scale\_succ,T\_scale\_fail>::type stan::prob::beta\_cdf\_log | ( | const T\_y & | *y*, |
|  |  | const T\_scale\_succ & | *alpha*, |
|  |  | const T\_scale\_fail & | *beta* |
|  | ) |  |  |

Definition at line 358 of file beta.hpp.

template<bool propto, typename T\_y , typename T\_scale\_succ , typename T\_scale\_fail >

|  |  |  |  |
| --- | --- | --- | --- |
| return\_type<T\_y,T\_scale\_succ,T\_scale\_fail>::type stan::prob::beta\_log | ( | const T\_y & | *y*, |
|  |  | const T\_scale\_succ & | *alpha*, |
|  |  | const T\_scale\_fail & | *beta* |
|  | ) |  |  |

The log of the beta density for the specified scalar(s) given the specified sample size(s).

y, alpha, or beta can each either be scalar or std::vector. Any vector inputs must be the same length.

The result log probability is defined to be the sum of the log probabilities for each observation/alpha/beta triple.

Prior sample sizes, alpha and beta, must be greater than 0.

Parameters
:   |  |  |
    | --- | --- |
    | y | (Sequence of) scalar(s). |
    | alpha | (Sequence of) prior sample size(s). |
    | beta | (Sequence of) prior sample size(s). |

Returns
:   The log of the product of densities.

Template Parameters
:   |  |  |
    | --- | --- |
    | T\_y | Type of scalar outcome. |
    | T\_scale\_succ | Type of prior scale for successes. |
    | T\_scale\_fail | Type of prior scale for failures. |

Error Policy:
:   See Error Handling Policies for details. Conditions:

    - All parameters must not be NaN.
    - alpha must be positive and finite.
    - beta must be positive and finite.

Definition at line 46 of file beta.hpp.

template<typename T\_y , typename T\_scale\_succ , typename T\_scale\_fail >

|  |  |  |  |
| --- | --- | --- | --- |
| return\_type<T\_y,T\_scale\_succ,T\_scale\_fail>::type stan::prob::beta\_log | ( | const T\_y & | *y*, |
|  |  | const T\_scale\_succ & | *alpha*, |
|  |  | const T\_scale\_fail & | *beta* |
|  | ) |  |  |

inline

Definition at line 194 of file beta.hpp.

template<class RNG >

|  |  |  |  |
| --- | --- | --- | --- |
| double stan::prob::beta\_rng | ( | const double | *alpha*, |
|  |  | const double | *beta*, |
|  |  | RNG & | *rng* |
|  | ) |  |  |

inline

Definition at line 593 of file beta.hpp.

template<typename T\_n , typename T\_N , typename T\_prob >

|  |  |  |  |
| --- | --- | --- | --- |
| return\_type<T\_prob>::type stan::prob::binomial\_ccdf\_log | ( | const T\_n & | *n*, |
|  |  | const T\_N & | *N*, |
|  |  | const T\_prob & | *theta* |
|  | ) |  |  |

Definition at line 399 of file binomial.hpp.

template<typename T\_n , typename T\_N , typename T\_prob >

|  |  |  |  |
| --- | --- | --- | --- |
| return\_type<T\_prob>::type stan::prob::binomial\_cdf | ( | const T\_n & | *n*, |
|  |  | const T\_N & | *N*, |
|  |  | const T\_prob & | *theta* |
|  | ) |  |  |

Definition at line 249 of file binomial.hpp.

template<typename T\_n , typename T\_N , typename T\_prob >

|  |  |  |  |
| --- | --- | --- | --- |
| return\_type<T\_prob>::type stan::prob::binomial\_cdf\_log | ( | const T\_n & | *n*, |
|  |  | const T\_N & | *N*, |
|  |  | const T\_prob & | *theta* |
|  | ) |  |  |

Definition at line 329 of file binomial.hpp.

template<bool propto, typename T\_n , typename T\_N , typename T\_prob >

|  |  |  |  |
| --- | --- | --- | --- |
| return\_type<T\_prob>::type stan::prob::binomial\_log | ( | const T\_n & | *n*, |
|  |  | const T\_N & | *N*, |
|  |  | const T\_prob & | *theta* |
|  | ) |  |  |

Definition at line 31 of file binomial.hpp.

template<typename T\_n , typename T\_N , typename T\_prob >

|  |  |  |  |
| --- | --- | --- | --- |
| return\_type<T\_prob>::type stan::prob::binomial\_log | ( | const T\_n & | *n*, |
|  |  | const T\_N & | *N*, |
|  |  | const T\_prob & | *theta* |
|  | ) |  |  |

inline

Definition at line 129 of file binomial.hpp.

template<bool propto, typename T\_n , typename T\_N , typename T\_prob >

|  |  |  |  |
| --- | --- | --- | --- |
| return\_type<T\_prob>::type stan::prob::binomial\_logit\_log | ( | const T\_n & | *n*, |
|  |  | const T\_N & | *N*, |
|  |  | const T\_prob & | *alpha* |
|  | ) |  |  |

Definition at line 142 of file binomial.hpp.

template<typename T\_n , typename T\_N , typename T\_prob >

|  |  |  |  |
| --- | --- | --- | --- |
| return\_type<T\_prob>::type stan::prob::binomial\_logit\_log | ( | const T\_n & | *n*, |
|  |  | const T\_N & | *N*, |
|  |  | const T\_prob & | *alpha* |
|  | ) |  |  |

inline

Definition at line 239 of file binomial.hpp.

template<class RNG >

|  |  |  |  |
| --- | --- | --- | --- |
| int stan::prob::binomial\_rng | ( | const int | *N*, |
|  |  | const double | *theta*, |
|  |  | RNG & | *rng* |
|  | ) |  |  |

inline

Definition at line 470 of file binomial.hpp.

template<bool propto, typename T\_prob >

|  |  |  |  |
| --- | --- | --- | --- |
| boost::math::tools::promote\_args<T\_prob>::type stan::prob::categorical\_log | ( | int | *n*, |
|  |  | const Eigen::Matrix< T\_prob, Eigen::Dynamic, 1 > & | *theta* |
|  | ) |  |  |

Definition at line 23 of file categorical.hpp.

template<typename T\_prob >

|  |  |  |  |
| --- | --- | --- | --- |
| boost::math::tools::promote\_args<T\_prob>::type stan::prob::categorical\_log | ( | const typename math::index\_type< Eigen::Matrix< T\_prob, Eigen::Dynamic, 1 > >::type | *n*, |
|  |  | const Eigen::Matrix< T\_prob, Eigen::Dynamic, 1 > & | *theta* |
|  | ) |  |  |

inline

Definition at line 59 of file categorical.hpp.

template<bool propto, typename T\_prob >

|  |  |  |  |
| --- | --- | --- | --- |
| boost::math::tools::promote\_args<T\_prob>::type stan::prob::categorical\_log | ( | const std::vector< int > & | *ns*, |
|  |  | const Eigen::Matrix< T\_prob, Eigen::Dynamic, 1 > & | *theta* |
|  | ) |  |  |

Definition at line 72 of file categorical.hpp.

template<typename T\_prob >

|  |  |  |  |
| --- | --- | --- | --- |
| boost::math::tools::promote\_args<T\_prob>::type stan::prob::categorical\_log | ( | const std::vector< int > & | *ns*, |
|  |  | const Eigen::Matrix< T\_prob, Eigen::Dynamic, 1 > & | *theta* |
|  | ) |  |  |

inline

Definition at line 124 of file categorical.hpp.

template<bool propto, typename T\_prob >

|  |  |  |  |
| --- | --- | --- | --- |
| boost::math::tools::promote\_args<T\_prob>::type stan::prob::categorical\_logit\_log | ( | int | *n*, |
|  |  | const Eigen::Matrix< T\_prob, Eigen::Dynamic, 1 > & | *beta* |
|  | ) |  |  |

Definition at line 21 of file categorical\_logit.hpp.

template<typename T\_prob >

|  |  |  |  |
| --- | --- | --- | --- |
| boost::math::tools::promote\_args<T\_prob>::type stan::prob::categorical\_logit\_log | ( | int | *n*, |
|  |  | const Eigen::Matrix< T\_prob, Eigen::Dynamic, 1 > & | *beta* |
|  | ) |  |  |

inline

Definition at line 46 of file categorical\_logit.hpp.

template<bool propto, typename T\_prob >

|  |  |  |  |
| --- | --- | --- | --- |
| boost::math::tools::promote\_args<T\_prob>::type stan::prob::categorical\_logit\_log | ( | const std::vector< int > & | *ns*, |
|  |  | const Eigen::Matrix< T\_prob, Eigen::Dynamic, 1 > & | *beta* |
|  | ) |  |  |

Definition at line 54 of file categorical\_logit.hpp.

template<typename T\_prob >

|  |  |  |  |
| --- | --- | --- | --- |
| boost::math::tools::promote\_args<T\_prob>::type stan::prob::categorical\_logit\_log | ( | const std::vector< int > & | *ns*, |
|  |  | const Eigen::Matrix< T\_prob, Eigen::Dynamic, 1 > & | *beta* |
|  | ) |  |  |

inline

Definition at line 91 of file categorical\_logit.hpp.

template<class RNG >

|  |  |  |  |
| --- | --- | --- | --- |
| int stan::prob::categorical\_rng | ( | const Eigen::Matrix< double, Eigen::Dynamic, 1 > & | *theta*, |
|  |  | RNG & | *rng* |
|  | ) |  |  |

inline

Definition at line 131 of file categorical.hpp.

template<typename T\_y , typename T\_loc , typename T\_scale >

|  |  |  |  |
| --- | --- | --- | --- |
| return\_type<T\_y,T\_loc,T\_scale>::type stan::prob::cauchy\_ccdf\_log | ( | const T\_y & | *y*, |
|  |  | const T\_loc & | *mu*, |
|  |  | const T\_scale & | *sigma* |
|  | ) |  |  |

Definition at line 322 of file cauchy.hpp.

template<typename T\_y , typename T\_loc , typename T\_scale >

|  |  |  |  |
| --- | --- | --- | --- |
| return\_type<T\_y,T\_loc,T\_scale>::type stan::prob::cauchy\_cdf | ( | const T\_y & | *y*, |
|  |  | const T\_loc & | *mu*, |
|  |  | const T\_scale & | *sigma* |
|  | ) |  |  |

Calculates the cauchy cumulative distribution function for the given variate, location, and scale.

Errors are configured by policy. All variables must be finite and the scale must be strictly greater than zero.

Parameters
:   |  |  |
    | --- | --- |
    | y | A scalar variate. |
    | mu | The location parameter. |
    | sigma | The scale parameter. |

Returns

Definition at line 158 of file cauchy.hpp.

template<typename T\_y , typename T\_loc , typename T\_scale >

|  |  |  |  |
| --- | --- | --- | --- |
| return\_type<T\_y,T\_loc,T\_scale>::type stan::prob::cauchy\_cdf\_log | ( | const T\_y & | *y*, |
|  |  | const T\_loc & | *mu*, |
|  |  | const T\_scale & | *sigma* |
|  | ) |  |  |

Definition at line 255 of file cauchy.hpp.

template<bool propto, typename T\_y , typename T\_loc , typename T\_scale >

|  |  |  |  |
| --- | --- | --- | --- |
| return\_type<T\_y,T\_loc,T\_scale>::type stan::prob::cauchy\_log | ( | const T\_y & | *y*, |
|  |  | const T\_loc & | *mu*, |
|  |  | const T\_scale & | *sigma* |
|  | ) |  |  |

The log of the Cauchy density for the specified scalar(s) given the specified location parameter(s) and scale parameter(s).

y, mu, or sigma can each either be scalar or std::vector. Any vector inputs must be the same length.

The result log probability is defined to be the sum of the log probabilities for each observation/mu/sigma triple.

Parameters
:   |  |  |
    | --- | --- |
    | y | (Sequence of) scalar(s). |
    | mu | (Sequence of) location(s). |
    | sigma | (Sequence of) scale(s). |

Returns
:   The log of the product of densities.

Template Parameters
:   |  |  |
    | --- | --- |
    | T\_y | Type of scalar outcome. |
    | T\_loc | Type of location. |
    | T\_scale | Type of scale. |

Error Policy:
:   See Error Handling Policies for details. Conditions:

    - All parameters must not be NaN.
    - sigma must be positive.

Definition at line 42 of file cauchy.hpp.

template<typename T\_y , typename T\_loc , typename T\_scale >

|  |  |  |  |
| --- | --- | --- | --- |
| return\_type<T\_y,T\_loc,T\_scale>::type stan::prob::cauchy\_log | ( | const T\_y & | *y*, |
|  |  | const T\_loc & | *mu*, |
|  |  | const T\_scale & | *sigma* |
|  | ) |  |  |

inline

Definition at line 136 of file cauchy.hpp.

template<class RNG >

|  |  |  |  |
| --- | --- | --- | --- |
| double stan::prob::cauchy\_rng | ( | const double | *mu*, |
|  |  | const double | *sigma*, |
|  |  | RNG & | *rng* |
|  | ) |  |  |

inline

Definition at line 388 of file cauchy.hpp.

template<typename T\_y , typename T\_dof >

|  |  |  |  |
| --- | --- | --- | --- |
| return\_type<T\_y,T\_dof>::type stan::prob::chi\_square\_ccdf\_log | ( | const T\_y & | *y*, |
|  |  | const T\_dof & | *nu* |
|  | ) |  |  |

Definition at line 348 of file chi\_square.hpp.

template<typename T\_y , typename T\_dof >

|  |  |  |  |
| --- | --- | --- | --- |
| return\_type<T\_y,T\_dof>::type stan::prob::chi\_square\_cdf | ( | const T\_y & | *y*, |
|  |  | const T\_dof & | *nu* |
|  | ) |  |  |

Calculates the chi square cumulative distribution function for the given variate and degrees of freedom.

y A scalar variate. nu Degrees of freedom.

Returns
:   The cdf of the chi square distribution

Definition at line 154 of file chi\_square.hpp.

template<typename T\_y , typename T\_dof >

|  |  |  |  |
| --- | --- | --- | --- |
| return\_type<T\_y,T\_dof>::type stan::prob::chi\_square\_cdf\_log | ( | const T\_y & | *y*, |
|  |  | const T\_dof & | *nu* |
|  | ) |  |  |

Definition at line 254 of file chi\_square.hpp.

template<bool propto, typename T\_y , typename T\_dof >

|  |  |  |  |
| --- | --- | --- | --- |
| return\_type<T\_y,T\_dof>::type stan::prob::chi\_square\_log | ( | const T\_y & | *y*, |
|  |  | const T\_dof & | *nu* |
|  | ) |  |  |

The log of a chi-squared density for y with the specified degrees of freedom parameter.

The degrees of freedom prarameter must be greater than 0. y must be greater than or equal to 0.

Parameters
:   |  |  |
    | --- | --- |
    | y | A scalar variable. |
    | nu | Degrees of freedom. |

Exceptions
:   |  |  |
    | --- | --- |
    | std::domain\_error | if nu is not greater than or equal to 0 |
    | std::domain\_error | if y is not greater than or equal to 0. |

Template Parameters
:   |  |  |
    | --- | --- |
    | T\_y | Type of scalar. |
    | T\_dof | Type of degrees of freedom. |

Definition at line 43 of file chi\_square.hpp.

template<typename T\_y , typename T\_dof >

|  |  |  |  |
| --- | --- | --- | --- |
| return\_type<T\_y,T\_dof>::type stan::prob::chi\_square\_log | ( | const T\_y & | *y*, |
|  |  | const T\_dof & | *nu* |
|  | ) |  |  |

inline

Definition at line 139 of file chi\_square.hpp.

template<class RNG >

|  |  |  |  |
| --- | --- | --- | --- |
| double stan::prob::chi\_square\_rng | ( | const double | *nu*, |
|  |  | RNG & | *rng* |
|  | ) |  |  |

inline

Definition at line 442 of file chi\_square.hpp.

template<typename T >

|  |  |  |  |
| --- | --- | --- | --- |
| Eigen::Matrix<T,Eigen::Dynamic,Eigen::Dynamic> stan::prob::cholesky\_corr\_constrain | ( | const Eigen::Matrix< T, Eigen::Dynamic, 1 > & | *y*, |
|  |  | int | *K* |
|  | ) |  |  |

Definition at line 1512 of file transform.hpp.

template<typename T >

|  |  |  |  |
| --- | --- | --- | --- |
| Eigen::Matrix<T,Eigen::Dynamic,Eigen::Dynamic> stan::prob::cholesky\_corr\_constrain | ( | const Eigen::Matrix< T, Eigen::Dynamic, 1 > & | *y*, |
|  |  | int | *K*, |
|  |  | T & | *lp* |
|  | ) |  |  |

Definition at line 1550 of file transform.hpp.

template<typename T >

|  |  |  |  |  |  |
| --- | --- | --- | --- | --- | --- |
| Eigen::Matrix<T,Eigen::Dynamic,1> stan::prob::cholesky\_corr\_free | ( | const Eigen::Matrix< T, Eigen::Dynamic, Eigen::Dynamic > & | *x* | ) |  |

Definition at line 1592 of file transform.hpp.

template<typename T >

|  |  |  |  |
| --- | --- | --- | --- |
| Eigen::Matrix<T,Eigen::Dynamic,Eigen::Dynamic> stan::prob::cholesky\_factor\_constrain | ( | const Eigen::Matrix< T, Eigen::Dynamic, 1 > & | *x*, |
|  |  | int | *M*, |
|  |  | int | *N* |
|  | ) |  |  |

Return the Cholesky factor of the specified size read from the specified vector.

A total of (N choose 2) + N + (M - N) \* N elements are required to read an M by N Cholesky factor.

Template Parameters
:   |  |  |
    | --- | --- |
    | T | Type of scalars in matrix |

Parameters
:   |  |  |
    | --- | --- |
    | x | Vector of unconstrained values |
    | M | Number of rows |
    | N | Number of columns |

Returns
:   Cholesky factor

Definition at line 1412 of file transform.hpp.

template<typename T >

|  |  |  |  |
| --- | --- | --- | --- |
| Eigen::Matrix<T,Eigen::Dynamic,Eigen::Dynamic> stan::prob::cholesky\_factor\_constrain | ( | const Eigen::Matrix< T, Eigen::Dynamic, 1 > & | *x*, |
|  |  | int | *M*, |
|  |  | int | *N*, |
|  |  | T & | *lp* |
|  | ) |  |  |

Return the Cholesky factor of the specified size read from the specified vector and increment the specified log probability reference with the log Jacobian adjustment of the transform.

A total of (N choose 2) + N + N \* (M - N) free parameters are required to read an M by N Cholesky factor.

Template Parameters
:   |  |  |
    | --- | --- |
    | T | Type of scalars in matrix |

Parameters
:   |  |  |
    | --- | --- |
    | x | Vector of unconstrained values |
    | M | Number of rows |
    | N | Number of columns |
    | lp | Log probability that is incremented with the log Jacobian |

Returns
:   Cholesky factor

Definition at line 1455 of file transform.hpp.

template<typename T >

|  |  |  |  |  |  |
| --- | --- | --- | --- | --- | --- |
| Eigen::Matrix<T,Eigen::Dynamic,1> stan::prob::cholesky\_factor\_free | ( | const Eigen::Matrix< T, Eigen::Dynamic, Eigen::Dynamic > & | *y* | ) |  |

Return the unconstrained vector of parameters correspdonding to the specified Cholesky factor.

A Cholesky factor must be lower triangular and have positive diagonal elements.

Parameters
:   |  |  |
    | --- | --- |
    | y | Cholesky factor. |

Returns
:   Unconstrained parameters for Cholesky factor.

Exceptions
:   |  |  |
    | --- | --- |
    | std::domain\_error | If the matrix is not a Cholesky factor. |

Definition at line 1485 of file transform.hpp.

template<typename T >

|  |  |  |  |  |  |
| --- | --- | --- | --- | --- | --- |
| T stan::prob::corr\_constrain | ( | const T | *x* | ) |  |

inline

Return the result of transforming the specified scalar to have a valid correlation value between -1 and 1 (inclusive).

The transform used is the hyperbolic tangent function,

.

Parameters
:   |  |  |
    | --- | --- |
    | x | Scalar input. |

Returns
:   Result of transforming the input to fall between -1 and 1.

Template Parameters
:   |  |  |
    | --- | --- |
    | T | Type of scalar. |

Definition at line 952 of file transform.hpp.

template<typename T >

|  |  |  |  |
| --- | --- | --- | --- |
| T stan::prob::corr\_constrain | ( | const T | *x*, |
|  |  | T & | *lp* |
|  | ) |  |  |

inline

Return the result of transforming the specified scalar to have a valid correlation value between -1 and 1 (inclusive).

The transform used is as specified for `corr_constrain(T)`. The log absolute Jacobian determinant is

.

Template Parameters
:   |  |  |
    | --- | --- |
    | T | Type of scalar. |

Definition at line 970 of file transform.hpp.

template<typename T >

|  |  |  |  |  |  |
| --- | --- | --- | --- | --- | --- |
| T stan::prob::corr\_free | ( | const T | *y* | ) |  |

inline

Return the unconstrained scalar that when transformed to a valid correlation produces the specified value.

This function inverts the transform defined for `corr_constrain(T)`, which is the inverse hyperbolic tangent,

.

Parameters
:   |  |  |
    | --- | --- |
    | y | Correlation scalar input. |

Returns
:   Free scalar that transforms to the specified input.

Template Parameters
:   |  |  |
    | --- | --- |
    | T | Type of scalar. |

Definition at line 995 of file transform.hpp.

template<typename T >

|  |  |  |  |
| --- | --- | --- | --- |
| Eigen::Matrix<T,Eigen::Dynamic,Eigen::Dynamic> stan::prob::corr\_matrix\_constrain | ( | const Eigen::Matrix< T, Eigen::Dynamic, 1 > & | *x*, |
|  |  | typename math::index\_type< Eigen::Matrix< T, Eigen::Dynamic, 1 > >::type | *k* |
|  | ) |  |  |

Return the correlation matrix of the specified dimensionality derived from the specified vector of unconstrained values.

The input vector must be of length . The values in the input vector represent unconstrained (partial) correlations among the dimensions.

The transform based on partial correlations is as specified in

- Lewandowski, Daniel, Dorota Kurowicka, and Harry Joe. 2009. Generating random correlation matrices based on vines and extended onion method. *Journal of Multivariate Analysis* **100**:1989–-2001.

The free vector entries are first constrained to be valid correlation values using `corr_constrain(T)`.

Parameters
:   |  |  |
    | --- | --- |
    | x | Vector of unconstrained partial correlations. |
    | k | Dimensionality of returned correlation matrix. |

Template Parameters
:   |  |  |
    | --- | --- |
    | T | Type of scalar. |

Exceptions
:   |  |  |
    | --- | --- |
    | std::invalid\_argument | if x is not a valid correlation matrix. |

Definition at line 1644 of file transform.hpp.

template<typename T >

|  |  |  |  |
| --- | --- | --- | --- |
| Eigen::Matrix<T,Eigen::Dynamic,Eigen::Dynamic> stan::prob::corr\_matrix\_constrain | ( | const Eigen::Matrix< T, Eigen::Dynamic, 1 > & | *x*, |
|  |  | typename math::index\_type< Eigen::Matrix< T, Eigen::Dynamic, 1 > >::type | *k*, |
|  |  | T & | *lp* |
|  | ) |  |  |

Return the correlation matrix of the specified dimensionality derived from the specified vector of unconstrained values.

The input vector must be of length . The values in the input vector represent unconstrained (partial) correlations among the dimensions.

The transform is as specified for `corr_matrix_constrain(Matrix,size_t)`; the paper it cites also defines the Jacobians for correlation inputs, which are composed with the correlation constrained Jacobians defined in `corr_constrain(T,double)` for this function.

Parameters
:   |  |  |
    | --- | --- |
    | x | Vector of unconstrained partial correlations. |
    | k | Dimensionality of returned correlation matrix. |
    | lp | Log probability reference to increment. |

Template Parameters
:   |  |  |
    | --- | --- |
    | T | Type of scalar. |

Definition at line 1682 of file transform.hpp.

template<typename T >

|  |  |  |  |  |  |
| --- | --- | --- | --- | --- | --- |
| Eigen::Matrix<T,Eigen::Dynamic,1> stan::prob::corr\_matrix\_free | ( | const Eigen::Matrix< T, Eigen::Dynamic, Eigen::Dynamic > & | *y* | ) |  |

Return the vector of unconstrained partial correlations that define the specified correlation matrix when transformed.

The constraining transform is defined as for `corr_matrix_constrain(Matrix,size_t)`. The inverse transform in this function is simpler in that it only needs to compute the  partial correlations and then free those.

Parameters
:   |  |  |
    | --- | --- |
    | y | The correlation matrix to free. |

Returns
:   Vector of unconstrained values that produce the specified correlation matrix when transformed.

Template Parameters
:   |  |  |
    | --- | --- |
    | T | Type of scalar. |

Exceptions
:   |  |  |
    | --- | --- |
    | std::domain\_error | if the correlation matrix has no elements or is not a square matrix. |
    | std::runtime\_error | if the correlation matrix cannot be factorized by factor\_cov\_matrix() or if the sds returned by factor\_cov\_matrix() on log scale are unconstrained. |

Definition at line 1722 of file transform.hpp.

template<typename T >

|  |  |  |  |
| --- | --- | --- | --- |
| Eigen::Matrix<T,Eigen::Dynamic,Eigen::Dynamic> stan::prob::cov\_matrix\_constrain | ( | const Eigen::Matrix< T, Eigen::Dynamic, 1 > & | *x*, |
|  |  | typename math::index\_type< Eigen::Matrix< T, Eigen::Dynamic, 1 > >::type | *K* |
|  | ) |  |  |

Return the symmetric, positive-definite matrix of dimensions K by K resulting from transforming the specified finite vector of size K plus (K choose 2).

See `cov_matrix_free()` for the inverse transform.

Parameters
:   |  |  |
    | --- | --- |
    | x | The vector to convert to a covariance matrix. |
    | K | The number of rows and columns of the resulting covariance matrix. |

Exceptions
:   |  |  |
    | --- | --- |
    | std::domain\_error | if (x.size() != K + (K choose 2)). |

Definition at line 1769 of file transform.hpp.

template<typename T >

|  |  |  |  |
| --- | --- | --- | --- |
| Eigen::Matrix<T,Eigen::Dynamic,Eigen::Dynamic> stan::prob::cov\_matrix\_constrain | ( | const Eigen::Matrix< T, Eigen::Dynamic, 1 > & | *x*, |
|  |  | typename math::index\_type< Eigen::Matrix< T, Eigen::Dynamic, Eigen::Dynamic > >::type | *K*, |
|  |  | T & | *lp* |
|  | ) |  |  |

Return the symmetric, positive-definite matrix of dimensions K by K resulting from transforming the specified finite vector of size K plus (K choose 2).

See `cov_matrix_free()` for the inverse transform.

Parameters
:   |  |  |
    | --- | --- |
    | x | The vector to convert to a covariance matrix. |
    | K | The dimensions of the resulting covariance matrix. |
    | lp | Reference |

Exceptions
:   |  |  |
    | --- | --- |
    | std::domain\_error | if (x.size() != K + (K choose 2)). |

Definition at line 1808 of file transform.hpp.

template<typename T >

|  |  |  |  |
| --- | --- | --- | --- |
| Eigen::Matrix<T,Eigen::Dynamic,Eigen::Dynamic> stan::prob::cov\_matrix\_constrain\_lkj | ( | const Eigen::Matrix< T, Eigen::Dynamic, 1 > & | *x*, |
|  |  | size\_t | *k* |
|  | ) |  |  |

Return the covariance matrix of the specified dimensionality derived from constraining the specified vector of unconstrained values.

The input vector must be of length . The first  values in the input represent unconstrained (partial) correlations and the last  are unconstrained standard deviations of the dimensions.

The transform scales the correlation matrix transform defined in `corr_matrix_constrain(Matrix,size_t)` with the constrained deviations.

Parameters
:   |  |  |
    | --- | --- |
    | x | Input vector of unconstrained partial correlations and standard deviations. |
    | k | Dimensionality of returned covariance matrix. |

Returns
:   Covariance matrix derived from the unconstrained partial correlations and deviations.

Template Parameters
:   |  |  |
    | --- | --- |
    | T | Type of scalar. |

Definition at line 1910 of file transform.hpp.

template<typename T >

|  |  |  |  |
| --- | --- | --- | --- |
| Eigen::Matrix<T,Eigen::Dynamic,Eigen::Dynamic> stan::prob::cov\_matrix\_constrain\_lkj | ( | const Eigen::Matrix< T, Eigen::Dynamic, 1 > & | *x*, |
|  |  | size\_t | *k*, |
|  |  | T & | *lp* |
|  | ) |  |  |

Return the covariance matrix of the specified dimensionality derived from constraining the specified vector of unconstrained values and increment the specified log probability reference with the log absolute Jacobian determinant.

The transform is defined as for `cov_matrix_constrain(Matrix,size_t)`.

The log absolute Jacobian determinant is derived by composing the log absolute Jacobian determinant for the underlying correlation matrix as defined in `cov_matrix_constrain(Matrix,size_t,T&)` with the Jacobian of the transfrom of the correlation matrix into a covariance matrix by scaling by standard deviations.

Parameters
:   |  |  |
    | --- | --- |
    | x | Input vector of unconstrained partial correlations and standard deviations. |
    | k | Dimensionality of returned covariance matrix. |
    | lp | Log probability reference to increment. |

Returns
:   Covariance matrix derived from the unconstrained partial correlations and deviations.

Template Parameters
:   |  |  |
    | --- | --- |
    | T | Type of scalar. |

Definition at line 1949 of file transform.hpp.

template<typename T >

|  |  |  |  |  |  |
| --- | --- | --- | --- | --- | --- |
| Eigen::Matrix<T,Eigen::Dynamic,1> stan::prob::cov\_matrix\_free | ( | const Eigen::Matrix< T, Eigen::Dynamic, Eigen::Dynamic > & | *y* | ) |  |

The covariance matrix derived from the symmetric view of the lower-triangular view of the K by K specified matrix is freed to return a vector of size K + (K choose 2).

This is the inverse of the `cov_matrix_constrain()` function so that for any finite vector `x` of size K

- (K choose 2),

`x == cov_matrix_free(cov_matrix_constrain(x,K))`.

In order for this round-trip to work (and really for this function to work), the symmetric view of its lower-triangular view must be positive definite.

Parameters
:   |  |  |
    | --- | --- |
    | y | Matrix of dimensions K by K such that he symmetric view of the lower-triangular view is positive definite. |

Returns
:   Vector of size K plus (K choose 2) in (-inf,inf) that produces

Exceptions
:   |  |  |
    | --- | --- |
    | std::domain\_error | if `y` is not square, has zero dimensionality, or has a non-positive diagonal element. |

Definition at line 1863 of file transform.hpp.

template<typename T >

|  |  |  |  |  |  |
| --- | --- | --- | --- | --- | --- |
| Eigen::Matrix<T,Eigen::Dynamic,1> stan::prob::cov\_matrix\_free\_lkj | ( | const Eigen::Matrix< T, Eigen::Dynamic, Eigen::Dynamic > & | *y* | ) |  |

Return the vector of unconstrained partial correlations and deviations that transform to the specified covariance matrix.

The constraining transform is defined as for `cov_matrix_constrain(Matrix,size_t)`. The inverse first factors out the deviations, then applies the freeing transfrom of `corr_matrix_free(Matrix&)`.

Parameters
:   |  |  |
    | --- | --- |
    | y | Covariance matrix to free. |

Returns
:   Vector of unconstrained values that transforms to the specified covariance matrix.

Template Parameters
:   |  |  |
    | --- | --- |
    | T | Type of scalar. |

Exceptions
:   |  |  |
    | --- | --- |
    | std::domain\_error | if the correlation matrix has no elements or is not a square matrix. |
    | std::runtime\_error | if the correlation matrix cannot be factorized by factor\_cov\_matrix() |

Definition at line 1983 of file transform.hpp.

template<bool propto, typename T\_prob , typename T\_prior\_sample\_size >

|  |  |  |  |
| --- | --- | --- | --- |
| boost::math::tools::promote\_args<T\_prob,T\_prior\_sample\_size>::type stan::prob::dirichlet\_log | ( | const Eigen::Matrix< T\_prob, Eigen::Dynamic, 1 > & | *theta*, |
|  |  | const Eigen::Matrix< T\_prior\_sample\_size, Eigen::Dynamic, 1 > & | *alpha* |
|  | ) |  |  |

The log of the Dirichlet density for the given theta and a vector of prior sample sizes, alpha.

Each element of alpha must be greater than 0. Each element of theta must be greater than or 0. Theta sums to 1.

Parameters
:   |  |  |
    | --- | --- |
    | theta | A scalar vector. |
    | alpha | Prior sample sizes. |

Returns
:   The log of the Dirichlet density.

Exceptions
:   |  |  |
    | --- | --- |
    | std::domain\_error | if any element of alpha is less than or equal to 0. |
    | std::domain\_error | if any element of theta is less than 0. |
    | std::domain\_error | if the sum of theta is not 1. |

Template Parameters
:   |  |  |
    | --- | --- |
    | T\_prob | Type of scalar. |
    | T\_prior\_sample\_size | Type of prior sample sizes. |

Definition at line 46 of file dirichlet.hpp.

template<typename T\_prob , typename T\_prior\_sample\_size >

|  |  |  |  |
| --- | --- | --- | --- |
| boost::math::tools::promote\_args<T\_prob,T\_prior\_sample\_size>::type stan::prob::dirichlet\_log | ( | const Eigen::Matrix< T\_prob, Eigen::Dynamic, 1 > & | *theta*, |
|  |  | const Eigen::Matrix< T\_prior\_sample\_size, Eigen::Dynamic, 1 > & | *alpha* |
|  | ) |  |  |

inline

Definition at line 77 of file dirichlet.hpp.

template<class RNG >

|  |  |  |  |
| --- | --- | --- | --- |
| Eigen::VectorXd stan::prob::dirichlet\_rng | ( | const Eigen::Matrix< double, Eigen::Dynamic, 1 > & | *alpha*, |
|  |  | RNG & | *rng* |
|  | ) |  |  |

inline

Definition at line 84 of file dirichlet.hpp.

template<typename T\_shape >

|  |  |  |  |
| --- | --- | --- | --- |
| T\_shape stan::prob::do\_lkj\_constant | ( | const T\_shape & | *eta*, |
|  |  | const unsigned int & | *K* |
|  | ) |  |  |

Definition at line 15 of file lkj\_corr.hpp.

template<typename T\_y , typename T\_loc , typename T\_scale >

|  |  |  |  |
| --- | --- | --- | --- |
| return\_type<T\_y,T\_loc,T\_scale>::type stan::prob::double\_exponential\_ccdf\_log | ( | const T\_y & | *y*, |
|  |  | const T\_loc & | *mu*, |
|  |  | const T\_scale & | *sigma* |
|  | ) |  |  |

Definition at line 291 of file double\_exponential.hpp.

template<typename T\_y , typename T\_loc , typename T\_scale >

|  |  |  |  |
| --- | --- | --- | --- |
| return\_type<T\_y,T\_loc,T\_scale>::type stan::prob::double\_exponential\_cdf | ( | const T\_y & | *y*, |
|  |  | const T\_loc & | *mu*, |
|  |  | const T\_scale & | *sigma* |
|  | ) |  |  |

Calculates the double exponential cumulative density function.

Parameters
:   |  |  |
    | --- | --- |
    | y | A scalar variate. |
    | mu | The location parameter. |
    | sigma | The scale parameter. |

Returns
:   The cumulative density function.

Definition at line 138 of file double\_exponential.hpp.

template<typename T\_y , typename T\_loc , typename T\_scale >

|  |  |  |  |
| --- | --- | --- | --- |
| return\_type<T\_y,T\_loc,T\_scale>::type stan::prob::double\_exponential\_cdf\_log | ( | const T\_y & | *y*, |
|  |  | const T\_loc & | *mu*, |
|  |  | const T\_scale & | *sigma* |
|  | ) |  |  |

Definition at line 215 of file double\_exponential.hpp.

template<bool propto, typename T\_y , typename T\_loc , typename T\_scale >

|  |  |  |  |
| --- | --- | --- | --- |
| return\_type<T\_y,T\_loc,T\_scale>::type stan::prob::double\_exponential\_log | ( | const T\_y & | *y*, |
|  |  | const T\_loc & | *mu*, |
|  |  | const T\_scale & | *sigma* |
|  | ) |  |  |

Definition at line 24 of file double\_exponential.hpp.

template<typename T\_y , typename T\_loc , typename T\_scale >

|  |  |  |  |
| --- | --- | --- | --- |
| return\_type<T\_y,T\_loc,T\_scale>::type stan::prob::double\_exponential\_log | ( | const T\_y & | *y*, |
|  |  | const T\_loc & | *mu*, |
|  |  | const T\_scale & | *sigma* |
|  | ) |  |  |

Definition at line 117 of file double\_exponential.hpp.

template<class RNG >

|  |  |  |  |
| --- | --- | --- | --- |
| double stan::prob::double\_exponential\_rng | ( | const double | *mu*, |
|  |  | const double | *sigma*, |
|  |  | RNG & | *rng* |
|  | ) |  |  |

inline

Definition at line 367 of file double\_exponential.hpp.

template<typename T\_y , typename T\_loc , typename T\_scale , typename T\_inv\_scale >

|  |  |  |  |
| --- | --- | --- | --- |
| return\_type<T\_y,T\_loc,T\_scale,T\_inv\_scale>::type stan::prob::exp\_mod\_normal\_ccdf\_log | ( | const T\_y & | *y*, |
|  |  | const T\_loc & | *mu*, |
|  |  | const T\_scale & | *sigma*, |
|  |  | const T\_inv\_scale & | *lambda* |
|  | ) |  |  |

Definition at line 356 of file exp\_mod\_normal.hpp.

template<typename T\_y , typename T\_loc , typename T\_scale , typename T\_inv\_scale >

|  |  |  |  |
| --- | --- | --- | --- |
| return\_type<T\_y,T\_loc,T\_scale,T\_inv\_scale>::type stan::prob::exp\_mod\_normal\_cdf | ( | const T\_y & | *y*, |
|  |  | const T\_loc & | *mu*, |
|  |  | const T\_scale & | *sigma*, |
|  |  | const T\_inv\_scale & | *lambda* |
|  | ) |  |  |

Definition at line 140 of file exp\_mod\_normal.hpp.

template<typename T\_y , typename T\_loc , typename T\_scale , typename T\_inv\_scale >

|  |  |  |  |
| --- | --- | --- | --- |
| return\_type<T\_y,T\_loc,T\_scale,T\_inv\_scale>::type stan::prob::exp\_mod\_normal\_cdf\_log | ( | const T\_y & | *y*, |
|  |  | const T\_loc & | *mu*, |
|  |  | const T\_scale & | *sigma*, |
|  |  | const T\_inv\_scale & | *lambda* |
|  | ) |  |  |

Definition at line 252 of file exp\_mod\_normal.hpp.

template<bool propto, typename T\_y , typename T\_loc , typename T\_scale , typename T\_inv\_scale >

|  |  |  |  |
| --- | --- | --- | --- |
| return\_type<T\_y,T\_loc,T\_scale, T\_inv\_scale>::type stan::prob::exp\_mod\_normal\_log | ( | const T\_y & | *y*, |
|  |  | const T\_loc & | *mu*, |
|  |  | const T\_scale & | *sigma*, |
|  |  | const T\_inv\_scale & | *lambda* |
|  | ) |  |  |

Definition at line 26 of file exp\_mod\_normal.hpp.

template<typename T\_y , typename T\_loc , typename T\_scale , typename T\_inv\_scale >

|  |  |  |  |
| --- | --- | --- | --- |
| return\_type<T\_y,T\_loc,T\_scale, T\_inv\_scale>::type stan::prob::exp\_mod\_normal\_log | ( | const T\_y & | *y*, |
|  |  | const T\_loc & | *mu*, |
|  |  | const T\_scale & | *sigma*, |
|  |  | const T\_inv\_scale & | *lambda* |
|  | ) |  |  |

inline

Definition at line 132 of file exp\_mod\_normal.hpp.

template<class RNG >

|  |  |  |  |
| --- | --- | --- | --- |
| double stan::prob::exp\_mod\_normal\_rng | ( | const double | *mu*, |
|  |  | const double | *sigma*, |
|  |  | const double | *lambda*, |
|  |  | RNG & | *rng* |
|  | ) |  |  |

inline

Definition at line 458 of file exp\_mod\_normal.hpp.

template<typename T\_y , typename T\_inv\_scale >

|  |  |  |  |
| --- | --- | --- | --- |
| return\_type<T\_y,T\_inv\_scale>::type stan::prob::exponential\_ccdf\_log | ( | const T\_y & | *y*, |
|  |  | const T\_inv\_scale & | *beta* |
|  | ) |  |  |

Definition at line 221 of file exponential.hpp.

template<typename T\_y , typename T\_inv\_scale >

|  |  |  |  |
| --- | --- | --- | --- |
| return\_type<T\_y,T\_inv\_scale>::type stan::prob::exponential\_cdf | ( | const T\_y & | *y*, |
|  |  | const T\_inv\_scale & | *beta* |
|  | ) |  |  |

Calculates the exponential cumulative distribution function for the given y and beta.

Inverse scale parameter must be greater than 0. y must be greater than or equal to 0.

Parameters
:   |  |  |
    | --- | --- |
    | y | A scalar variable. |
    | beta | Inverse scale parameter. |

Template Parameters
:   |  |  |
    | --- | --- |
    | T\_y | Type of scalar. |
    | T\_inv\_scale | Type of inverse scale. |
    | Policy | Error-handling policy. |

Definition at line 122 of file exponential.hpp.

template<typename T\_y , typename T\_inv\_scale >

|  |  |  |  |
| --- | --- | --- | --- |
| return\_type<T\_y,T\_inv\_scale>::type stan::prob::exponential\_cdf\_log | ( | const T\_y & | *y*, |
|  |  | const T\_inv\_scale & | *beta* |
|  | ) |  |  |

Definition at line 175 of file exponential.hpp.

template<bool propto, typename T\_y , typename T\_inv\_scale >

|  |  |  |  |
| --- | --- | --- | --- |
| return\_type<T\_y,T\_inv\_scale>::type stan::prob::exponential\_log | ( | const T\_y & | *y*, |
|  |  | const T\_inv\_scale & | *beta* |
|  | ) |  |  |

The log of an exponential density for y with the specified inverse scale parameter.

Inverse scale parameter must be greater than 0. y must be greater than or equal to 0.

Parameters
:   |  |  |
    | --- | --- |
    | y | A scalar variable. |
    | beta | Inverse scale parameter. |

Exceptions
:   |  |  |
    | --- | --- |
    | std::domain\_error | if beta is not greater than 0. |
    | std::domain\_error | if y is not greater than or equal to 0. |

Template Parameters
:   |  |  |
    | --- | --- |
    | T\_y | Type of scalar. |
    | T\_inv\_scale | Type of inverse scale. |

Definition at line 46 of file exponential.hpp.

template<typename T\_y , typename T\_inv\_scale >

|  |  |  |  |
| --- | --- | --- | --- |
| return\_type<T\_y,T\_inv\_scale>::type stan::prob::exponential\_log | ( | const T\_y & | *y*, |
|  |  | const T\_inv\_scale & | *beta* |
|  | ) |  |  |

inline

Definition at line 101 of file exponential.hpp.

template<class RNG >

|  |  |  |  |
| --- | --- | --- | --- |
| double stan::prob::exponential\_rng | ( | const double | *beta*, |
|  |  | RNG & | *rng* |
|  | ) |  |  |

inline

Definition at line 265 of file exponential.hpp.

template<typename T >

|  |  |  |  |
| --- | --- | --- | --- |
| bool stan::prob::factor\_cov\_matrix | ( | const Eigen::Matrix< T, Eigen::Dynamic, Eigen::Dynamic > & | *Sigma*, |
|  |  | Eigen::Array< T, Eigen::Dynamic, 1 > & | *CPCs*, |
|  |  | Eigen::Array< T, Eigen::Dynamic, 1 > & | *sds* |
|  | ) |  |  |

This function is intended to make starting values, given a covariance matrix Sigma.

The transformations are hard coded as log for standard deviations and Fisher transformations (atanh()) of CPCs

Parameters
:   |  |  |  |
    | --- | --- | --- |
    | [in] | Sigma | covariance matrix |
    | [out] | CPCs | fill this unbounded (does not resize) |
    | [out] | sds | fill this unbounded (does not resize) |

Returns
:   false if any of the diagonals of Sigma are 0

Definition at line 88 of file transform.hpp.

template<typename T >

|  |  |  |  |
| --- | --- | --- | --- |
| void stan::prob::factor\_U | ( | const Eigen::Matrix< T, Eigen::Dynamic, Eigen::Dynamic > & | *U*, |
|  |  | Eigen::Array< T, Eigen::Dynamic, 1 > & | *CPCs* |
|  | ) |  |  |

This function is intended to make starting values, given a unit upper-triangular matrix U such that U'DU is a correlation matrix.

Parameters
:   |  |  |
    | --- | --- |
    | CPCs | fill this unbounded |
    | Sigma | U matrix |

Definition at line 43 of file transform.hpp.

template<typename T\_y , typename T\_shape , typename T\_scale >

|  |  |  |  |
| --- | --- | --- | --- |
| return\_type<T\_y,T\_shape,T\_scale>::type stan::prob::frechet\_ccdf\_log | ( | const T\_y & | *y*, |
|  |  | const T\_shape & | *alpha*, |
|  |  | const T\_scale & | *sigma* |
|  | ) |  |  |

Definition at line 244 of file frechet.hpp.

template<typename T\_y , typename T\_shape , typename T\_scale >

|  |  |  |  |
| --- | --- | --- | --- |
| return\_type<T\_y,T\_shape,T\_scale>::type stan::prob::frechet\_cdf | ( | const T\_y & | *y*, |
|  |  | const T\_shape & | *alpha*, |
|  |  | const T\_scale & | *sigma* |
|  | ) |  |  |

Definition at line 131 of file frechet.hpp.

template<typename T\_y , typename T\_shape , typename T\_scale >

|  |  |  |  |
| --- | --- | --- | --- |
| return\_type<T\_y,T\_shape,T\_scale>::type stan::prob::frechet\_cdf\_log | ( | const T\_y & | *y*, |
|  |  | const T\_shape & | *alpha*, |
|  |  | const T\_scale & | *sigma* |
|  | ) |  |  |

Definition at line 193 of file frechet.hpp.

template<bool propto, typename T\_y , typename T\_shape , typename T\_scale >

|  |  |  |  |
| --- | --- | --- | --- |
| return\_type<T\_y,T\_shape,T\_scale>::type stan::prob::frechet\_log | ( | const T\_y & | *y*, |
|  |  | const T\_shape & | *alpha*, |
|  |  | const T\_scale & | *sigma* |
|  | ) |  |  |

Definition at line 24 of file frechet.hpp.

template<typename T\_y , typename T\_shape , typename T\_scale >

|  |  |  |  |
| --- | --- | --- | --- |
| return\_type<T\_y,T\_shape,T\_scale>::type stan::prob::frechet\_log | ( | const T\_y & | *y*, |
|  |  | const T\_shape & | *alpha*, |
|  |  | const T\_scale & | *sigma* |
|  | ) |  |  |

inline

Definition at line 125 of file frechet.hpp.

template<class RNG >

|  |  |  |  |
| --- | --- | --- | --- |
| double stan::prob::frechet\_rng | ( | const double | *alpha*, |
|  |  | const double | *sigma*, |
|  |  | RNG & | *rng* |
|  | ) |  |  |

inline

Definition at line 297 of file frechet.hpp.

template<typename T\_y , typename T\_shape , typename T\_inv\_scale >

|  |  |  |  |
| --- | --- | --- | --- |
| return\_type<T\_y,T\_shape,T\_inv\_scale>::type stan::prob::gamma\_ccdf\_log | ( | const T\_y & | *y*, |
|  |  | const T\_shape & | *alpha*, |
|  |  | const T\_inv\_scale & | *beta* |
|  | ) |  |  |

Definition at line 388 of file gamma.hpp.

template<typename T\_y , typename T\_shape , typename T\_inv\_scale >

|  |  |  |  |
| --- | --- | --- | --- |
| return\_type<T\_y,T\_shape,T\_inv\_scale>::type stan::prob::gamma\_cdf | ( | const T\_y & | *y*, |
|  |  | const T\_shape & | *alpha*, |
|  |  | const T\_inv\_scale & | *beta* |
|  | ) |  |  |

The cumulative density function for a gamma distribution for y with the specified shape and inverse scale parameters.

Parameters
:   |  |  |
    | --- | --- |
    | y | A scalar variable. |
    | alpha | Shape parameter. |
    | beta | Inverse scale parameter. |

Exceptions
:   |  |  |
    | --- | --- |
    | std::domain\_error | if alpha is not greater than 0. |
    | std::domain\_error | if beta is not greater than 0. |
    | std::domain\_error | if y is not greater than or equal to 0. |

Template Parameters
:   |  |  |
    | --- | --- |
    | T\_y | Type of scalar. |
    | T\_shape | Type of shape. |
    | T\_inv\_scale | Type of inverse scale. |

Definition at line 172 of file gamma.hpp.

template<typename T\_y , typename T\_shape , typename T\_inv\_scale >

|  |  |  |  |
| --- | --- | --- | --- |
| return\_type<T\_y,T\_shape,T\_inv\_scale>::type stan::prob::gamma\_cdf\_log | ( | const T\_y & | *y*, |
|  |  | const T\_shape & | *alpha*, |
|  |  | const T\_inv\_scale & | *beta* |
|  | ) |  |  |

Definition at line 285 of file gamma.hpp.

template<bool propto, typename T\_y , typename T\_shape , typename T\_inv\_scale >

|  |  |  |  |
| --- | --- | --- | --- |
| return\_type<T\_y,T\_shape,T\_inv\_scale>::type stan::prob::gamma\_log | ( | const T\_y & | *y*, |
|  |  | const T\_shape & | *alpha*, |
|  |  | const T\_inv\_scale & | *beta* |
|  | ) |  |  |

The log of a gamma density for y with the specified shape and inverse scale parameters.

Shape and inverse scale parameters must be greater than 0. y must be greater than or equal to 0.

Parameters
:   |  |  |
    | --- | --- |
    | y | A scalar variable. |
    | alpha | Shape parameter. |
    | beta | Inverse scale parameter. |

Exceptions
:   |  |  |
    | --- | --- |
    | std::domain\_error | if alpha is not greater than 0. |
    | std::domain\_error | if beta is not greater than 0. |
    | std::domain\_error | if y is not greater than or equal to 0. |

Template Parameters
:   |  |  |
    | --- | --- |
    | T\_y | Type of scalar. |
    | T\_shape | Type of shape. |
    | T\_inv\_scale | Type of inverse scale. |

Definition at line 46 of file gamma.hpp.

template<typename T\_y , typename T\_shape , typename T\_inv\_scale >

|  |  |  |  |
| --- | --- | --- | --- |
| return\_type<T\_y,T\_shape,T\_inv\_scale>::type stan::prob::gamma\_log | ( | const T\_y & | *y*, |
|  |  | const T\_shape & | *alpha*, |
|  |  | const T\_inv\_scale & | *beta* |
|  | ) |  |  |

inline

Definition at line 151 of file gamma.hpp.

template<class RNG >

|  |  |  |  |
| --- | --- | --- | --- |
| double stan::prob::gamma\_rng | ( | const double | *alpha*, |
|  |  | const double | *beta*, |
|  |  | RNG & | *rng* |
|  | ) |  |  |

inline

Definition at line 491 of file gamma.hpp.

template<bool propto, typename T\_y , typename T\_F , typename T\_G , typename T\_V , typename T\_W , typename T\_m0 , typename T\_C0 >

|  |  |  |  |
| --- | --- | --- | --- |
| return\_type< T\_y, typename return\_type<T\_F,T\_G,T\_V,T\_W,T\_m0,T\_C0>::type >::type stan::prob::gaussian\_dlm\_obs\_log | ( | const Eigen::Matrix< T\_y, Eigen::Dynamic, Eigen::Dynamic > & | *y*, |
|  |  | const Eigen::Matrix< T\_F, Eigen::Dynamic, Eigen::Dynamic > & | *F*, |
|  |  | const Eigen::Matrix< T\_G, Eigen::Dynamic, Eigen::Dynamic > & | *G*, |
|  |  | const Eigen::Matrix< T\_V, Eigen::Dynamic, Eigen::Dynamic > & | *V*, |
|  |  | const Eigen::Matrix< T\_W, Eigen::Dynamic, Eigen::Dynamic > & | *W*, |
|  |  | const Eigen::Matrix< T\_m0, Eigen::Dynamic, 1 > & | *m0*, |
|  |  | const Eigen::Matrix< T\_C0, Eigen::Dynamic, Eigen::Dynamic > & | *C0* |
|  | ) |  |  |

The log of a Gaussian dynamic linear model (GDLM).

This distribution is equivalent to, for ,

If V is a vector, then the Kalman filter is applied sequentially.

Parameters
:   |  |  |
    | --- | --- |
    | y | A r x T matrix of observations. Rows are variables, columns are observations. |
    | F | A n x r matrix. The design matrix. |
    | G | A n x n matrix. The transition matrix. |
    | V | A r x r matrix. The observation covariance matrix. |
    | W | A n x n matrix. The state covariance matrix. |
    | m0 | A n x 1 matrix. The mean vector of the distribution of the initial state. |
    | C0 | A n x n matrix. The covariance matrix of the distribution of the initial state. |

Returns
:   The log of the joint density of the GDLM.

Exceptions
:   |  |  |
    | --- | --- |
    | std::domain\_error | if a matrix in the Kalman filter is not positive semi-definite. |

Template Parameters
:   |  |  |
    | --- | --- |
    | T\_y | Type of scalar. |
    | T\_F | Type of design matrix. |
    | T\_G | Type of transition matrix. |
    | T\_V | Type of observation covariance matrix. |
    | T\_W | Type of state covariance matrix. |
    | T\_m0 | Type of initial state mean vector. |
    | T\_C0 | Type of initial state covariance matrix. |

Definition at line 80 of file gaussian\_dlm\_obs.hpp.

template<typename T\_y , typename T\_F , typename T\_G , typename T\_V , typename T\_W , typename T\_m0 , typename T\_C0 >

|  |  |  |  |
| --- | --- | --- | --- |
| return\_type< T\_y, typename return\_type<T\_F,T\_G,T\_V,T\_W,T\_m0,T\_C0>::type >::type stan::prob::gaussian\_dlm\_obs\_log | ( | const Eigen::Matrix< T\_y, Eigen::Dynamic, Eigen::Dynamic > & | *y*, |
|  |  | const Eigen::Matrix< T\_F, Eigen::Dynamic, Eigen::Dynamic > & | *F*, |
|  |  | const Eigen::Matrix< T\_G, Eigen::Dynamic, Eigen::Dynamic > & | *G*, |
|  |  | const Eigen::Matrix< T\_V, Eigen::Dynamic, Eigen::Dynamic > & | *V*, |
|  |  | const Eigen::Matrix< T\_W, Eigen::Dynamic, Eigen::Dynamic > & | *W*, |
|  |  | const Eigen::Matrix< T\_m0, Eigen::Dynamic, 1 > & | *m0*, |
|  |  | const Eigen::Matrix< T\_C0, Eigen::Dynamic, Eigen::Dynamic > & | *C0* |
|  | ) |  |  |

inline

Definition at line 234 of file gaussian\_dlm\_obs.hpp.

template<bool propto, typename T\_y , typename T\_F , typename T\_G , typename T\_V , typename T\_W , typename T\_m0 , typename T\_C0 >

|  |  |  |  |
| --- | --- | --- | --- |
| return\_type< T\_y, typename return\_type<T\_F,T\_G,T\_V,T\_W,T\_m0,T\_C0>::type >::type stan::prob::gaussian\_dlm\_obs\_log | ( | const Eigen::Matrix< T\_y, Eigen::Dynamic, Eigen::Dynamic > & | *y*, |
|  |  | const Eigen::Matrix< T\_F, Eigen::Dynamic, Eigen::Dynamic > & | *F*, |
|  |  | const Eigen::Matrix< T\_G, Eigen::Dynamic, Eigen::Dynamic > & | *G*, |
|  |  | const Eigen::Matrix< T\_V, Eigen::Dynamic, 1 > & | *V*, |
|  |  | const Eigen::Matrix< T\_W, Eigen::Dynamic, Eigen::Dynamic > & | *W*, |
|  |  | const Eigen::Matrix< T\_m0, Eigen::Dynamic, 1 > & | *m0*, |
|  |  | const Eigen::Matrix< T\_C0, Eigen::Dynamic, Eigen::Dynamic > & | *C0* |
|  | ) |  |  |

The log of a Gaussian dynamic linear model (GDLM) with uncorrelated observation disturbances.

This distribution is equivalent to, for ,

If V is a vector, then the Kalman filter is applied sequentially.

Parameters
:   |  |  |
    | --- | --- |
    | y | A r x T matrix of observations. Rows are variables, columns are observations. |
    | F | A n x r matrix. The design matrix. |
    | G | A n x n matrix. The transition matrix. |
    | V | A size r vector. The diagonal of the observation covariance matrix. |
    | W | A n x n matrix. The state covariance matrix. |
    | m0 | A n x 1 matrix. The mean vector of the distribution of the initial state. |
    | C0 | A n x n matrix. The covariance matrix of the distribution of the initial state. |

Returns
:   The log of the joint density of the GDLM.

Exceptions
:   |  |  |
    | --- | --- |
    | std::domain\_error | if a matrix in the Kalman filter is not semi-positive definite. |

Template Parameters
:   |  |  |
    | --- | --- |
    | T\_y | Type of scalar. |
    | T\_F | Type of design matrix. |
    | T\_G | Type of transition matrix. |
    | T\_V | Type of observation variances |
    | T\_W | Type of state covariance matrix. |
    | T\_m0 | Type of initial state mean vector. |
    | T\_C0 | Type of initial state covariance matrix. |

Definition at line 288 of file gaussian\_dlm\_obs.hpp.

template<typename T\_y , typename T\_F , typename T\_G , typename T\_V , typename T\_W , typename T\_m0 , typename T\_C0 >

|  |  |  |  |
| --- | --- | --- | --- |
| return\_type< T\_y, typename return\_type<T\_F,T\_G,T\_V,T\_W,T\_m0,T\_C0>::type >::type stan::prob::gaussian\_dlm\_obs\_log | ( | const Eigen::Matrix< T\_y, Eigen::Dynamic, Eigen::Dynamic > & | *y*, |
|  |  | const Eigen::Matrix< T\_F, Eigen::Dynamic, Eigen::Dynamic > & | *F*, |
|  |  | const Eigen::Matrix< T\_G, Eigen::Dynamic, Eigen::Dynamic > & | *G*, |
|  |  | const Eigen::Matrix< T\_V, Eigen::Dynamic, 1 > & | *V*, |
|  |  | const Eigen::Matrix< T\_W, Eigen::Dynamic, Eigen::Dynamic > & | *W*, |
|  |  | const Eigen::Matrix< T\_m0, Eigen::Dynamic, 1 > & | *m0*, |
|  |  | const Eigen::Matrix< T\_C0, Eigen::Dynamic, Eigen::Dynamic > & | *C0* |
|  | ) |  |  |

inline

Definition at line 447 of file gaussian\_dlm\_obs.hpp.

template<typename T\_y , typename T\_loc , typename T\_scale >

|  |  |  |  |
| --- | --- | --- | --- |
| return\_type<T\_y,T\_loc,T\_scale>::type stan::prob::gumbel\_ccdf\_log | ( | const T\_y & | *y*, |
|  |  | const T\_loc & | *mu*, |
|  |  | const T\_scale & | *beta* |
|  | ) |  |  |

Definition at line 234 of file gumbel.hpp.

template<typename T\_y , typename T\_loc , typename T\_scale >

|  |  |  |  |
| --- | --- | --- | --- |
| return\_type<T\_y,T\_loc,T\_scale>::type stan::prob::gumbel\_cdf | ( | const T\_y & | *y*, |
|  |  | const T\_loc & | *mu*, |
|  |  | const T\_scale & | *beta* |
|  | ) |  |  |

Definition at line 113 of file gumbel.hpp.

template<typename T\_y , typename T\_loc , typename T\_scale >

|  |  |  |  |
| --- | --- | --- | --- |
| return\_type<T\_y,T\_loc,T\_scale>::type stan::prob::gumbel\_cdf\_log | ( | const T\_y & | *y*, |
|  |  | const T\_loc & | *mu*, |
|  |  | const T\_scale & | *beta* |
|  | ) |  |  |

Definition at line 181 of file gumbel.hpp.

template<bool propto, typename T\_y , typename T\_loc , typename T\_scale >

|  |  |  |  |
| --- | --- | --- | --- |
| return\_type<T\_y,T\_loc,T\_scale>::type stan::prob::gumbel\_log | ( | const T\_y & | *y*, |
|  |  | const T\_loc & | *mu*, |
|  |  | const T\_scale & | *beta* |
|  | ) |  |  |

Definition at line 21 of file gumbel.hpp.

template<typename T\_y , typename T\_loc , typename T\_scale >

|  |  |  |  |
| --- | --- | --- | --- |
| return\_type<T\_y,T\_loc,T\_scale>::type stan::prob::gumbel\_log | ( | const T\_y & | *y*, |
|  |  | const T\_loc & | *mu*, |
|  |  | const T\_scale & | *beta* |
|  | ) |  |  |

inline

Definition at line 107 of file gumbel.hpp.

template<class RNG >

|  |  |  |  |
| --- | --- | --- | --- |
| double stan::prob::gumbel\_rng | ( | const double | *mu*, |
|  |  | const double | *beta*, |
|  |  | RNG & | *rng* |
|  | ) |  |  |

inline

Definition at line 289 of file gumbel.hpp.

template<bool propto, typename T\_n , typename T\_N , typename T\_a , typename T\_b >

|  |  |  |  |
| --- | --- | --- | --- |
| double stan::prob::hypergeometric\_log | ( | const T\_n & | *n*, |
|  |  | const T\_N & | *N*, |
|  |  | const T\_a & | *a*, |
|  |  | const T\_b & | *b* |
|  | ) |  |  |

Definition at line 25 of file hypergeometric.hpp.

template<typename T\_n , typename T\_N , typename T\_a , typename T\_b >

|  |  |  |  |
| --- | --- | --- | --- |
| double stan::prob::hypergeometric\_log | ( | const T\_n & | *n*, |
|  |  | const T\_N & | *N*, |
|  |  | const T\_a & | *a*, |
|  |  | const T\_b & | *b* |
|  | ) |  |  |

inline

Definition at line 83 of file hypergeometric.hpp.

template<class RNG >

|  |  |  |  |
| --- | --- | --- | --- |
| int stan::prob::hypergeometric\_rng | ( | const int | *N*, |
|  |  | const int | *a*, |
|  |  | const int | *b*, |
|  |  | RNG & | *rng* |
|  | ) |  |  |

inline

Definition at line 92 of file hypergeometric.hpp.

template<typename T >

|  |  |  |  |  |  |
| --- | --- | --- | --- | --- | --- |
| T stan::prob::identity\_constrain | ( | T | *x* | ) |  |

inline

Returns the result of applying the identity constraint transform to the input.

This method is effectively a no-op and is mainly useful as a placeholder in auto-generated code.

Parameters
:   |  |  |
    | --- | --- |
    | x | Free scalar. |

Returns
:   Transformed input.

Template Parameters
:   |  |  |
    | --- | --- |
    | T | Type of scalar. |

Definition at line 391 of file transform.hpp.

template<typename T >

|  |  |  |  |
| --- | --- | --- | --- |
| T stan::prob::identity\_constrain | ( | const T | *x*, |
|  |  | T & |  |
|  | ) |  |  |

inline

Returns the result of applying the identity constraint transform to the input and increments the log probability reference with the log absolute Jacobian determinant.

This method is effectively a no-op and mainly useful as a placeholder in auto-generated code.

Parameters
:   |  |  |
    | --- | --- |
    | x | Free scalar. lp Reference to log probability. |

Returns
:   Transformed input.

Template Parameters
:   |  |  |
    | --- | --- |
    | T | Type of scalar. |

Definition at line 410 of file transform.hpp.

template<typename T >

|  |  |  |  |  |  |
| --- | --- | --- | --- | --- | --- |
| T stan::prob::identity\_free | ( | const T | *y* | ) |  |

inline

Returns the result of applying the inverse of the identity constraint transform to the input.

This method is effectively a no-op and mainly useful as a placeholder in auto-generated code.

Parameters
:   |  |  |
    | --- | --- |
    | y | Constrained scalar. |

Returns
:   The input.

Template Parameters
:   |  |  |
    | --- | --- |
    | T | Type of scalar. |

Definition at line 427 of file transform.hpp.

template<typename T\_y , typename T\_dof >

|  |  |  |  |
| --- | --- | --- | --- |
| return\_type<T\_y,T\_dof>::type stan::prob::inv\_chi\_square\_ccdf\_log | ( | const T\_y & | *y*, |
|  |  | const T\_dof & | *nu* |
|  | ) |  |  |

Definition at line 332 of file inv\_chi\_square.hpp.

template<typename T\_y , typename T\_dof >

|  |  |  |  |
| --- | --- | --- | --- |
| return\_type<T\_y,T\_dof>::type stan::prob::inv\_chi\_square\_cdf | ( | const T\_y & | *y*, |
|  |  | const T\_dof & | *nu* |
|  | ) |  |  |

Definition at line 137 of file inv\_chi\_square.hpp.

template<typename T\_y , typename T\_dof >

|  |  |  |  |
| --- | --- | --- | --- |
| return\_type<T\_y,T\_dof>::type stan::prob::inv\_chi\_square\_cdf\_log | ( | const T\_y & | *y*, |
|  |  | const T\_dof & | *nu* |
|  | ) |  |  |

Definition at line 239 of file inv\_chi\_square.hpp.

template<bool propto, typename T\_y , typename T\_dof >

|  |  |  |  |
| --- | --- | --- | --- |
| return\_type<T\_y,T\_dof>::type stan::prob::inv\_chi\_square\_log | ( | const T\_y & | *y*, |
|  |  | const T\_dof & | *nu* |
|  | ) |  |  |

The log of an inverse chi-squared density for y with the specified degrees of freedom parameter.

The degrees of freedom prarameter must be greater than 0. y must be greater than 0.

Parameters
:   |  |  |
    | --- | --- |
    | y | A scalar variable. |
    | nu | Degrees of freedom. |

Exceptions
:   |  |  |
    | --- | --- |
    | std::domain\_error | if nu is not greater than or equal to 0 |
    | std::domain\_error | if y is not greater than or equal to 0. |

Template Parameters
:   |  |  |
    | --- | --- |
    | T\_y | Type of scalar. |
    | T\_dof | Type of degrees of freedom. |

Definition at line 43 of file inv\_chi\_square.hpp.

template<typename T\_y , typename T\_dof >

|  |  |  |  |
| --- | --- | --- | --- |
| return\_type<T\_y,T\_dof>::type stan::prob::inv\_chi\_square\_log | ( | const T\_y & | *y*, |
|  |  | const T\_dof & | *nu* |
|  | ) |  |  |

inline

Definition at line 131 of file inv\_chi\_square.hpp.

template<class RNG >

|  |  |  |  |
| --- | --- | --- | --- |
| double stan::prob::inv\_chi\_square\_rng | ( | const double | *nu*, |
|  |  | RNG & | *rng* |
|  | ) |  |  |

inline

Definition at line 425 of file inv\_chi\_square.hpp.

template<typename T\_y , typename T\_shape , typename T\_scale >

|  |  |  |  |
| --- | --- | --- | --- |
| return\_type<T\_y,T\_shape,T\_scale>::type stan::prob::inv\_gamma\_ccdf\_log | ( | const T\_y & | *y*, |
|  |  | const T\_shape & | *alpha*, |
|  |  | const T\_scale & | *beta* |
|  | ) |  |  |

Definition at line 401 of file inv\_gamma.hpp.

template<typename T\_y , typename T\_shape , typename T\_scale >

|  |  |  |  |
| --- | --- | --- | --- |
| return\_type<T\_y,T\_shape,T\_scale>::type stan::prob::inv\_gamma\_cdf | ( | const T\_y & | *y*, |
|  |  | const T\_shape & | *alpha*, |
|  |  | const T\_scale & | *beta* |
|  | ) |  |  |

The CDF of an inverse gamma density for y with the specified shape and scale parameters.

y, shape, and scale parameters must be greater than 0.

Parameters
:   |  |  |
    | --- | --- |
    | y | A scalar variable. |
    | alpha | Shape parameter. |
    | beta | Scale parameter. |

Exceptions
:   |  |  |
    | --- | --- |
    | std::domain\_error | if alpha is not greater than 0. |
    | std::domain\_error | if beta is not greater than 0. |
    | std::domain\_error | if y is not greater than 0. |

Template Parameters
:   |  |  |
    | --- | --- |
    | T\_y | Type of scalar. |
    | T\_shape | Type of shape. |
    | T\_scale | Type of scale. |

Definition at line 177 of file inv\_gamma.hpp.

template<typename T\_y , typename T\_shape , typename T\_scale >

|  |  |  |  |
| --- | --- | --- | --- |
| return\_type<T\_y,T\_shape,T\_scale>::type stan::prob::inv\_gamma\_cdf\_log | ( | const T\_y & | *y*, |
|  |  | const T\_shape & | *alpha*, |
|  |  | const T\_scale & | *beta* |
|  | ) |  |  |

Definition at line 294 of file inv\_gamma.hpp.

template<bool propto, typename T\_y , typename T\_shape , typename T\_scale >

|  |  |  |  |
| --- | --- | --- | --- |
| return\_type<T\_y,T\_shape,T\_scale>::type stan::prob::inv\_gamma\_log | ( | const T\_y & | *y*, |
|  |  | const T\_shape & | *alpha*, |
|  |  | const T\_scale & | *beta* |
|  | ) |  |  |

The log of an inverse gamma density for y with the specified shape and scale parameters.

Shape and scale parameters must be greater than 0. y must be greater than 0.

Parameters
:   |  |  |
    | --- | --- |
    | y | A scalar variable. |
    | alpha | Shape parameter. |
    | beta | Scale parameter. |

Exceptions
:   |  |  |
    | --- | --- |
    | std::domain\_error | if alpha is not greater than 0. |
    | std::domain\_error | if beta is not greater than 0. |
    | std::domain\_error | if y is not greater than 0. |

Template Parameters
:   |  |  |
    | --- | --- |
    | T\_y | Type of scalar. |
    | T\_shape | Type of shape. |
    | T\_scale | Type of scale. |

Definition at line 40 of file inv\_gamma.hpp.

template<typename T\_y , typename T\_shape , typename T\_scale >

|  |  |  |  |
| --- | --- | --- | --- |
| return\_type<T\_y,T\_shape,T\_scale>::type stan::prob::inv\_gamma\_log | ( | const T\_y & | *y*, |
|  |  | const T\_shape & | *alpha*, |
|  |  | const T\_scale & | *beta* |
|  | ) |  |  |

inline

Definition at line 155 of file inv\_gamma.hpp.

template<class RNG >

|  |  |  |  |
| --- | --- | --- | --- |
| double stan::prob::inv\_gamma\_rng | ( | const double | *alpha*, |
|  |  | const double | *beta*, |
|  |  | RNG & | *rng* |
|  | ) |  |  |

inline

Definition at line 508 of file inv\_gamma.hpp.

template<bool propto, typename T\_y , typename T\_dof , typename T\_scale >

|  |  |  |  |
| --- | --- | --- | --- |
| boost::math::tools::promote\_args<T\_y,T\_dof,T\_scale>::type stan::prob::inv\_wishart\_log | ( | const Eigen::Matrix< T\_y, Eigen::Dynamic, Eigen::Dynamic > & | *W*, |
|  |  | const T\_dof & | *nu*, |
|  |  | const Eigen::Matrix< T\_scale, Eigen::Dynamic, Eigen::Dynamic > & | *S* |
|  | ) |  |  |

The log of the Inverse-Wishart density for the given W, degrees of freedom, and scale matrix.

The scale matrix, S, must be k x k, symmetric, and semi-positive definite.

Parameters
:   |  |  |
    | --- | --- |
    | W | A scalar matrix |
    | nu | Degrees of freedom |
    | S | The scale matrix |

Returns
:   The log of the Inverse-Wishart density at W given nu and S.

Exceptions
:   |  |  |
    | --- | --- |
    | std::domain\_error | if nu is not greater than k-1 |
    | std::domain\_error | if S is not square, not symmetric, or not semi-positive definite. |

Template Parameters
:   |  |  |
    | --- | --- |
    | T\_y | Type of scalar. |
    | T\_dof | Type of degrees of freedom. |
    | T\_scale | Type of scale. |

Definition at line 52 of file inv\_wishart.hpp.

template<typename T\_y , typename T\_dof , typename T\_scale >

|  |  |  |  |
| --- | --- | --- | --- |
| boost::math::tools::promote\_args<T\_y,T\_dof,T\_scale>::type stan::prob::inv\_wishart\_log | ( | const Eigen::Matrix< T\_y, Eigen::Dynamic, Eigen::Dynamic > & | *W*, |
|  |  | const T\_dof & | *nu*, |
|  |  | const Eigen::Matrix< T\_scale, Eigen::Dynamic, Eigen::Dynamic > & | *S* |
|  | ) |  |  |

inline

Definition at line 125 of file inv\_wishart.hpp.

template<class RNG >

|  |  |  |  |
| --- | --- | --- | --- |
| Eigen::Matrix<double, Eigen::Dynamic, Eigen::Dynamic> stan::prob::inv\_wishart\_rng | ( | const double | *nu*, |
|  |  | const Eigen::Matrix< double, Eigen::Dynamic, Eigen::Dynamic > & | *S*, |
|  |  | RNG & | *rng* |
|  | ) |  |  |

inline

Definition at line 133 of file inv\_wishart.hpp.

template<typename T , typename TL >

|  |  |  |  |
| --- | --- | --- | --- |
| T stan::prob::lb\_constrain | ( | const T | *x*, |
|  |  | const TL | *lb* |
|  | ) |  |  |

inline

Return the lower-bounded value for the specified unconstrained input and specified lower bound.

The transform applied is

where  is the constant lower bound.

If the lower bound is negative infinity, this function reduces to `identity_constrain(x)`.

Parameters
:   |  |  |
    | --- | --- |
    | x | Unconstrained scalar input. |
    | lb | Lower-bound on constrained ouptut. |

Returns
:   Lower-bound constrained value correspdonding to inputs.

Template Parameters
:   |  |  |
    | --- | --- |
    | T | Type of scalar. |
    | TL | Type of lower bound. |

Definition at line 520 of file transform.hpp.

template<typename T , typename TL >

|  |  |  |  |
| --- | --- | --- | --- |
| boost::math::tools::promote\_args<T,TL>::type stan::prob::lb\_constrain | ( | const T | *x*, |
|  |  | const TL | *lb*, |
|  |  | T & | *lp* |
|  | ) |  |  |

inline

Return the lower-bounded value for the speicifed unconstrained input and specified lower bound, incrementing the specified reference with the log absolute Jacobian determinant of the transform.

If the lower bound is negative infinity, this function reduces to `identity_constraint(x,lp)`.

Parameters
:   |  |  |
    | --- | --- |
    | x | Unconstrained scalar input. |
    | lb | Lower-bound on output. |
    | lp | Reference to log probability to increment. |

Returns
:   Loer-bound constrained value corresponding to inputs.

Template Parameters
:   |  |  |
    | --- | --- |
    | T | Type of scalar. |
    | TL | Type of lower bound. |

Definition at line 545 of file transform.hpp.

template<typename T , typename TL >

|  |  |  |  |
| --- | --- | --- | --- |
| boost::math::tools::promote\_args<T,TL>::type stan::prob::lb\_free | ( | const T | *y*, |
|  |  | const TL | *lb* |
|  | ) |  |  |

inline

Return the unconstrained value that produces the specified lower-bound constrained value.

If the lower bound is negative infinity, it is ignored and the function reduces to `identity_free(y)`.

Parameters
:   |  |  |
    | --- | --- |
    | y | Input scalar. |
    | lb | Lower bound. |

Returns
:   Unconstrained value that produces the input when constrained.

Template Parameters
:   |  |  |
    | --- | --- |
    | T | Type of scalar. |
    | TL | Type of lower bound. |

Exceptions
:   |  |  |
    | --- | --- |
    | std::domain\_error | if y is lower than the lower bound. |

Definition at line 570 of file transform.hpp.

template<bool propto, typename T\_covar , typename T\_shape >

|  |  |  |  |
| --- | --- | --- | --- |
| boost::math::tools::promote\_args<T\_covar, T\_shape>::type stan::prob::lkj\_corr\_cholesky\_log | ( | const Eigen::Matrix< T\_covar, Eigen::Dynamic, Eigen::Dynamic > & | *L*, |
|  |  | const T\_shape & | *eta* |
|  | ) |  |  |

Definition at line 48 of file lkj\_corr.hpp.

template<typename T\_covar , typename T\_shape >

|  |  |  |  |
| --- | --- | --- | --- |
| boost::math::tools::promote\_args<T\_covar, T\_shape>::type stan::prob::lkj\_corr\_cholesky\_log | ( | const Eigen::Matrix< T\_covar, Eigen::Dynamic, Eigen::Dynamic > & | *L*, |
|  |  | const T\_shape & | *eta* |
|  | ) |  |  |

inline

Definition at line 92 of file lkj\_corr.hpp.

template<class RNG >

|  |  |  |  |
| --- | --- | --- | --- |
| Eigen::MatrixXd stan::prob::lkj\_corr\_cholesky\_rng | ( | const size\_t | *K*, |
|  |  | const double | *eta*, |
|  |  | RNG & | *rng* |
|  | ) |  |  |

inline

Definition at line 153 of file lkj\_corr.hpp.

template<bool propto, typename T\_y , typename T\_shape >

|  |  |  |  |
| --- | --- | --- | --- |
| boost::math::tools::promote\_args<T\_y, T\_shape>::type stan::prob::lkj\_corr\_log | ( | const Eigen::Matrix< T\_y, Eigen::Dynamic, Eigen::Dynamic > & | *y*, |
|  |  | const T\_shape & | *eta* |
|  | ) |  |  |

Definition at line 103 of file lkj\_corr.hpp.

template<typename T\_y , typename T\_shape >

|  |  |  |  |
| --- | --- | --- | --- |
| boost::math::tools::promote\_args<T\_y, T\_shape>::type stan::prob::lkj\_corr\_log | ( | const Eigen::Matrix< T\_y, Eigen::Dynamic, Eigen::Dynamic > & | *y*, |
|  |  | const T\_shape & | *eta* |
|  | ) |  |  |

inline

Definition at line 146 of file lkj\_corr.hpp.

template<class RNG >

|  |  |  |  |
| --- | --- | --- | --- |
| Eigen::MatrixXd stan::prob::lkj\_corr\_rng | ( | const size\_t | *K*, |
|  |  | const double | *eta*, |
|  |  | RNG & | *rng* |
|  | ) |  |  |

inline

Definition at line 178 of file lkj\_corr.hpp.

template<bool propto, typename T\_y , typename T\_loc , typename T\_scale , typename T\_shape >

|  |  |  |  |
| --- | --- | --- | --- |
| boost::math::tools::promote\_args<T\_y,T\_loc,T\_scale,T\_shape>::type stan::prob::lkj\_cov\_log | ( | const Eigen::Matrix< T\_y, Eigen::Dynamic, Eigen::Dynamic > & | *y*, |
|  |  | const Eigen::Matrix< T\_loc, Eigen::Dynamic, 1 > & | *mu*, |
|  |  | const Eigen::Matrix< T\_scale, Eigen::Dynamic, 1 > & | *sigma*, |
|  |  | const T\_shape & | *eta* |
|  | ) |  |  |

Definition at line 23 of file lkj\_cov.hpp.

template<typename T\_y , typename T\_loc , typename T\_scale , typename T\_shape >

|  |  |  |  |
| --- | --- | --- | --- |
| boost::math::tools::promote\_args<T\_y,T\_loc,T\_scale,T\_shape>::type stan::prob::lkj\_cov\_log | ( | const Eigen::Matrix< T\_y, Eigen::Dynamic, Eigen::Dynamic > & | *y*, |
|  |  | const Eigen::Matrix< T\_loc, Eigen::Dynamic, 1 > & | *mu*, |
|  |  | const Eigen::Matrix< T\_scale, Eigen::Dynamic, 1 > & | *sigma*, |
|  |  | const T\_shape & | *eta* |
|  | ) |  |  |

inline

Definition at line 76 of file lkj\_cov.hpp.

template<bool propto, typename T\_y , typename T\_loc , typename T\_scale , typename T\_shape >

|  |  |  |  |
| --- | --- | --- | --- |
| boost::math::tools::promote\_args<T\_y,T\_loc,T\_scale,T\_shape>::type stan::prob::lkj\_cov\_log | ( | const Eigen::Matrix< T\_y, Eigen::Dynamic, Eigen::Dynamic > & | *y*, |
|  |  | const T\_loc & | *mu*, |
|  |  | const T\_scale & | *sigma*, |
|  |  | const T\_shape & | *eta* |
|  | ) |  |  |

Definition at line 88 of file lkj\_cov.hpp.

template<typename T\_y , typename T\_loc , typename T\_scale , typename T\_shape >

|  |  |  |  |
| --- | --- | --- | --- |
| boost::math::tools::promote\_args<T\_y,T\_loc,T\_scale,T\_shape>::type stan::prob::lkj\_cov\_log | ( | const Eigen::Matrix< T\_y, Eigen::Dynamic, Eigen::Dynamic > & | *y*, |
|  |  | const T\_loc & | *mu*, |
|  |  | const T\_scale & | *sigma*, |
|  |  | const T\_shape & | *eta* |
|  | ) |  |  |

inline

Definition at line 124 of file lkj\_cov.hpp.

template<typename T >

|  |  |  |  |
| --- | --- | --- | --- |
| T stan::prob::log\_inv\_logit\_diff | ( | const T & | *alpha*, |
|  |  | const T & | *beta* |
|  | ) |  |  |

inline

Definition at line 24 of file ordered\_logistic.hpp.

template<typename T\_y , typename T\_loc , typename T\_scale >

|  |  |  |  |
| --- | --- | --- | --- |
| return\_type<T\_y, T\_loc, T\_scale>::type stan::prob::logistic\_ccdf\_log | ( | const T\_y & | *y*, |
|  |  | const T\_loc & | *mu*, |
|  |  | const T\_scale & | *sigma* |
|  | ) |  |  |

Definition at line 315 of file logistic.hpp.

template<typename T\_y , typename T\_loc , typename T\_scale >

|  |  |  |  |
| --- | --- | --- | --- |
| return\_type<T\_y, T\_loc, T\_scale>::type stan::prob::logistic\_cdf | ( | const T\_y & | *y*, |
|  |  | const T\_loc & | *mu*, |
|  |  | const T\_scale & | *sigma* |
|  | ) |  |  |

Definition at line 143 of file logistic.hpp.

template<typename T\_y , typename T\_loc , typename T\_scale >

|  |  |  |  |
| --- | --- | --- | --- |
| return\_type<T\_y, T\_loc, T\_scale>::type stan::prob::logistic\_cdf\_log | ( | const T\_y & | *y*, |
|  |  | const T\_loc & | *mu*, |
|  |  | const T\_scale & | *sigma* |
|  | ) |  |  |

Definition at line 237 of file logistic.hpp.

template<bool propto, typename T\_y , typename T\_loc , typename T\_scale >

|  |  |  |  |
| --- | --- | --- | --- |
| return\_type<T\_y,T\_loc,T\_scale>::type stan::prob::logistic\_log | ( | const T\_y & | *y*, |
|  |  | const T\_loc & | *mu*, |
|  |  | const T\_scale & | *sigma* |
|  | ) |  |  |

Definition at line 27 of file logistic.hpp.

template<typename T\_y , typename T\_loc , typename T\_scale >

|  |  |  |  |
| --- | --- | --- | --- |
| return\_type<T\_y,T\_loc,T\_scale>::type stan::prob::logistic\_log | ( | const T\_y & | *y*, |
|  |  | const T\_loc & | *mu*, |
|  |  | const T\_scale & | *sigma* |
|  | ) |  |  |

inline

Definition at line 136 of file logistic.hpp.

template<class RNG >

|  |  |  |  |
| --- | --- | --- | --- |
| double stan::prob::logistic\_rng | ( | const double | *mu*, |
|  |  | const double | *sigma*, |
|  |  | RNG & | *rng* |
|  | ) |  |  |

inline

Definition at line 393 of file logistic.hpp.

template<typename T\_y , typename T\_loc , typename T\_scale >

|  |  |  |  |
| --- | --- | --- | --- |
| return\_type<T\_y,T\_loc,T\_scale>::type stan::prob::lognormal\_ccdf\_log | ( | const T\_y & | *y*, |
|  |  | const T\_loc & | *mu*, |
|  |  | const T\_scale & | *sigma* |
|  | ) |  |  |

Definition at line 292 of file lognormal.hpp.

template<typename T\_y , typename T\_loc , typename T\_scale >

|  |  |  |  |
| --- | --- | --- | --- |
| return\_type<T\_y,T\_loc,T\_scale>::type stan::prob::lognormal\_cdf | ( | const T\_y & | *y*, |
|  |  | const T\_loc & | *mu*, |
|  |  | const T\_scale & | *sigma* |
|  | ) |  |  |

Definition at line 150 of file lognormal.hpp.

template<typename T\_y , typename T\_loc , typename T\_scale >

|  |  |  |  |
| --- | --- | --- | --- |
| return\_type<T\_y,T\_loc,T\_scale>::type stan::prob::lognormal\_cdf\_log | ( | const T\_y & | *y*, |
|  |  | const T\_loc & | *mu*, |
|  |  | const T\_scale & | *sigma* |
|  | ) |  |  |

Definition at line 225 of file lognormal.hpp.

template<bool propto, typename T\_y , typename T\_loc , typename T\_scale >

|  |  |  |  |
| --- | --- | --- | --- |
| return\_type<T\_y,T\_loc,T\_scale>::type stan::prob::lognormal\_log | ( | const T\_y & | *y*, |
|  |  | const T\_loc & | *mu*, |
|  |  | const T\_scale & | *sigma* |
|  | ) |  |  |

Definition at line 24 of file lognormal.hpp.

template<typename T\_y , typename T\_loc , typename T\_scale >

|  |  |  |  |
| --- | --- | --- | --- |
| return\_type<T\_y,T\_loc,T\_scale>::type stan::prob::lognormal\_log | ( | const T\_y & | *y*, |
|  |  | const T\_loc & | *mu*, |
|  |  | const T\_scale & | *sigma* |
|  | ) |  |  |

inline

Definition at line 143 of file lognormal.hpp.

template<class RNG >

|  |  |  |  |
| --- | --- | --- | --- |
| double stan::prob::lognormal\_rng | ( | const double | *mu*, |
|  |  | const double | *sigma*, |
|  |  | RNG & | *rng* |
|  | ) |  |  |

inline

Definition at line 360 of file lognormal.hpp.

template<typename T , typename TL , typename TU >

|  |  |  |  |
| --- | --- | --- | --- |
| boost::math::tools::promote\_args<T,TL,TU>::type stan::prob::lub\_constrain | ( | const T | *x*, |
|  |  | TL | *lb*, |
|  |  | TU | *ub* |
|  | ) |  |  |

inline

Return the lower- and upper-bounded scalar derived by transforming the specified free scalar given the specified lower and upper bounds.

The transform is the transformed and scaled inverse logit,

If the lower bound is negative infinity and upper bound finite, this function reduces to `ub_constrain(x,ub)`. If the upper bound is positive infinity and the lower bound finite, this function reduces to `lb_constrain(x,lb)`. If the upper bound is positive infinity and the lower bound negative infinity, this function reduces to `identity_constrain(x)`.

Parameters
:   |  |  |
    | --- | --- |
    | x | Free scalar to transform. |
    | lb | Lower bound. |
    | ub | Upper bound. |

Returns
:   Lower- and upper-bounded scalar derived from transforming the free scalar.

Template Parameters
:   |  |  |
    | --- | --- |
    | T | Type of scalar. |
    | TL | Type of lower bound. |
    | TU | Type of upper bound. |

Exceptions
:   |  |  |
    | --- | --- |
    | std::domain\_error | if ub <= lb |

Definition at line 710 of file transform.hpp.

template<typename T , typename TL , typename TU >

|  |  |  |  |
| --- | --- | --- | --- |
| boost::math::tools::promote\_args<T,TL,TU>::type stan::prob::lub\_constrain | ( | const T | *x*, |
|  |  | const TL | *lb*, |
|  |  | const TU | *ub*, |
|  |  | T & | *lp* |
|  | ) |  |  |

Return the lower- and upper-bounded scalar derived by transforming the specified free scalar given the specified lower and upper bounds and increment the specified log probability with the log absolute Jacobian determinant.

The transform is as defined in `lub_constrain(T,double,double)`. The log absolute Jacobian determinant is given by

If the lower bound is negative infinity and upper bound finite, this function reduces to `ub_constrain(x,ub,lp)`. If the upper bound is positive infinity and the lower bound finite, this function reduces to `lb_constrain(x,lb,lp)`. If the upper bound is positive infinity and the lower bound negative infinity, this function reduces to `identity_constrain(x,lp)`.

Parameters
:   |  |  |
    | --- | --- |
    | x | Free scalar to transform. |
    | lb | Lower bound. |
    | ub | Upper bound. |
    | lp | Log probability scalar reference. |

Returns
:   Lower- and upper-bounded scalar derived from transforming the free scalar.

Template Parameters
:   |  |  |
    | --- | --- |
    | T | Type of scalar. |
    | TL | Type of lower bound. |
    | TU | Type of upper bound. |

Exceptions
:   |  |  |
    | --- | --- |
    | std::domain\_error | if ub <= lb |

Definition at line 780 of file transform.hpp.

template<typename T , typename TL , typename TU >

|  |  |  |  |
| --- | --- | --- | --- |
| boost::math::tools::promote\_args<T,TL,TU>::type stan::prob::lub\_free | ( | const T | *y*, |
|  |  | TL | *lb*, |
|  |  | TU | *ub* |
|  | ) |  |  |

inline

Return the unconstrained scalar that transforms to the specified lower- and upper-bounded scalar given the specified bounds.

The transfrom in `lub_constrain(T,double,double)`, is reversed by a transformed and scaled logit,

where  and  are the lower and upper bounds.

If the lower bound is negative infinity and upper bound finite, this function reduces to `ub_free(y,ub)`. If the upper bound is positive infinity and the lower bound finite, this function reduces to `lb_free(x,lb)`. If the upper bound is positive infinity and the lower bound negative infinity, this function reduces to `identity_free(y)`.

Template Parameters
:   |  |  |
    | --- | --- |
    | T | Type of scalar. |

Parameters
:   |  |  |
    | --- | --- |
    | y | Scalar input. |
    | lb | Lower bound. |
    | ub | Upper bound. |

Returns
:   The free scalar that transforms to the input scalar given the bounds.

Exceptions
:   |  |  |
    | --- | --- |
    | std::invalid\_argument | if the lower bound is greater than the upper bound, y is less than the lower bound, or y is greater than the upper bound |

Definition at line 845 of file transform.hpp.

template<typename T >

|  |  |  |  |
| --- | --- | --- | --- |
| const Eigen::Array<T,Eigen::Dynamic,1> stan::prob::make\_nu | ( | const T | *eta*, |
|  |  | const size\_t | *K* |
|  | ) |  |  |

This function calculates the degrees of freedom for the t distribution that corresponds to the shape parameter in the Lewandowski et.

al. distribution

Parameters
:   |  |  |
    | --- | --- |
    | eta | hyperparameter on (0,inf), eta = 1 <-> correlation matrix is uniform |
    | K | number of variables in covariance matrix |

Definition at line 343 of file transform.hpp.

template<bool propto, typename T\_y , typename T\_Mu , typename T\_Sigma , typename T\_D >

|  |  |  |  |
| --- | --- | --- | --- |
| boost::math::tools::promote\_args<T\_y,T\_Mu,T\_Sigma,T\_D>::type stan::prob::matrix\_normal\_prec\_log | ( | const Eigen::Matrix< T\_y, Eigen::Dynamic, Eigen::Dynamic > & | *y*, |
|  |  | const Eigen::Matrix< T\_Mu, Eigen::Dynamic, Eigen::Dynamic > & | *Mu*, |
|  |  | const Eigen::Matrix< T\_Sigma, Eigen::Dynamic, Eigen::Dynamic > & | *Sigma*, |
|  |  | const Eigen::Matrix< T\_D, Eigen::Dynamic, Eigen::Dynamic > & | *D* |
|  | ) |  |  |

The log of the matrix normal density for the given y, mu, Sigma and D where Sigma and D are given as precision matrices, not covariance matrices.

Parameters
:   |  |  |
    | --- | --- |
    | y | An mxn matrix. |
    | Mu | The mean matrix. |
    | Sigma | The mxm inverse covariance matrix (i.e., the precision matrix) of the rows of y. |
    | D | The nxn inverse covariance matrix (i.e., the precision matrix) of the columns of y. |

Returns
:   The log of the matrix normal density.

Exceptions
:   |  |  |
    | --- | --- |
    | std::domain\_error | if Sigma or D are not square, not symmetric, or not semi-positive definite. |

Template Parameters
:   |  |  |
    | --- | --- |
    | T\_y | Type of scalar. |
    | T\_Mu | Type of location. |
    | T\_Sigma | Type of Sigma. |
    | T\_D | Type of D. |

Definition at line 41 of file matrix\_normal.hpp.

template<typename T\_y , typename T\_Mu , typename T\_Sigma , typename T\_D >

|  |  |  |  |
| --- | --- | --- | --- |
| boost::math::tools::promote\_args<T\_y,T\_Mu,T\_Sigma,T\_D>::type stan::prob::matrix\_normal\_prec\_log | ( | const Eigen::Matrix< T\_y, Eigen::Dynamic, Eigen::Dynamic > & | *y*, |
|  |  | const Eigen::Matrix< T\_Mu, Eigen::Dynamic, Eigen::Dynamic > & | *Mu*, |
|  |  | const Eigen::Matrix< T\_Sigma, Eigen::Dynamic, Eigen::Dynamic > & | *Sigma*, |
|  |  | const Eigen::Matrix< T\_D, Eigen::Dynamic, Eigen::Dynamic > & | *D* |
|  | ) |  |  |

Definition at line 117 of file matrix\_normal.hpp.

template<bool propto, typename T\_y , typename T\_covar , typename T\_w >

|  |  |  |  |
| --- | --- | --- | --- |
| boost::math::tools::promote\_args<T\_y,T\_covar,T\_w>::type stan::prob::multi\_gp\_cholesky\_log | ( | const Eigen::Matrix< T\_y, Eigen::Dynamic, Eigen::Dynamic > & | *y*, |
|  |  | const Eigen::Matrix< T\_covar, Eigen::Dynamic, Eigen::Dynamic > & | *L*, |
|  |  | const Eigen::Matrix< T\_w, Eigen::Dynamic, 1 > & | *w* |
|  | ) |  |  |

The log of a multivariate Gaussian Process for the given y, w, and a Cholesky factor L of the kernel matrix Sigma.

Sigma = LL', a square, semi-positive definite matrix.. y is a dxN matrix, where each column is a different observation and each row is a different output dimension. The Gaussian Process is assumed to have a scaled kernel matrix with a different scale for each output dimension. This distribution is equivalent to: for (i in 1:d) row(y,i) ~ multi\_normal(0,(1/w[i])\*LL').

Parameters
:   |  |  |
    | --- | --- |
    | y | A dxN matrix |
    | L | The Cholesky decomposition of a kernel matrix |
    | w | A d-dimensional vector of positve inverse scale parameters for each output. |

Returns
:   The log of the multivariate GP density.

Exceptions
:   |  |  |
    | --- | --- |
    | std::domain\_error | if Sigma is not square, not symmetric, or not semi-positive definite. |

Template Parameters
:   |  |  |
    | --- | --- |
    | T\_y | Type of scalar. |
    | T\_covar | Type of kernel. |
    | T\_w | Type of weight. |

Definition at line 45 of file multi\_gp\_cholesky.hpp.

template<typename T\_y , typename T\_covar , typename T\_w >

|  |  |  |  |
| --- | --- | --- | --- |
| boost::math::tools::promote\_args<T\_y,T\_covar,T\_w>::type stan::prob::multi\_gp\_cholesky\_log | ( | const Eigen::Matrix< T\_y, Eigen::Dynamic, Eigen::Dynamic > & | *y*, |
|  |  | const Eigen::Matrix< T\_covar, Eigen::Dynamic, Eigen::Dynamic > & | *L*, |
|  |  | const Eigen::Matrix< T\_w, Eigen::Dynamic, 1 > & | *w* |
|  | ) |  |  |

inline

Definition at line 106 of file multi\_gp\_cholesky.hpp.

template<bool propto, typename T\_y , typename T\_covar , typename T\_w >

|  |  |  |  |
| --- | --- | --- | --- |
| boost::math::tools::promote\_args<T\_y,T\_covar,T\_w>::type stan::prob::multi\_gp\_log | ( | const Eigen::Matrix< T\_y, Eigen::Dynamic, Eigen::Dynamic > & | *y*, |
|  |  | const Eigen::Matrix< T\_covar, Eigen::Dynamic, Eigen::Dynamic > & | *Sigma*, |
|  |  | const Eigen::Matrix< T\_w, Eigen::Dynamic, 1 > & | *w* |
|  | ) |  |  |

The log of a multivariate Gaussian Process for the given y, Sigma, and w.

y is a dxN matrix, where each column is a different observation and each row is a different output dimension. The Gaussian Process is assumed to have a scaled kernel matrix with a different scale for each output dimension. This distribution is equivalent to: for (i in 1:d) row(y,i) ~ multi\_normal(0,(1/w[i])\*Sigma).

Parameters
:   |  |  |
    | --- | --- |
    | y | A dxN matrix |
    | Sigma | The NxN kernel matrix |
    | w | A d-dimensional vector of positve inverse scale parameters for each output. |

Returns
:   The log of the multivariate GP density.

Exceptions
:   |  |  |
    | --- | --- |
    | std::domain\_error | if Sigma is not square, not symmetric, or not semi-positive definite. |

Template Parameters
:   |  |  |
    | --- | --- |
    | T\_y | Type of scalar. |
    | T\_covar | Type of kernel. |
    | T\_w | Type of weight. |

Definition at line 45 of file multi\_gp.hpp.

template<typename T\_y , typename T\_covar , typename T\_w >

|  |  |  |  |
| --- | --- | --- | --- |
| boost::math::tools::promote\_args<T\_y,T\_covar,T\_w>::type stan::prob::multi\_gp\_log | ( | const Eigen::Matrix< T\_y, Eigen::Dynamic, Eigen::Dynamic > & | *y*, |
|  |  | const Eigen::Matrix< T\_covar, Eigen::Dynamic, Eigen::Dynamic > & | *Sigma*, |
|  |  | const Eigen::Matrix< T\_w, Eigen::Dynamic, 1 > & | *w* |
|  | ) |  |  |

inline

Definition at line 115 of file multi\_gp.hpp.

template<bool propto, typename T\_y , typename T\_loc , typename T\_covar >

|  |  |  |  |
| --- | --- | --- | --- |
| boost::math::tools::promote\_args<typename scalar\_type<T\_y>::type, typename scalar\_type<T\_loc>::type, T\_covar>::type stan::prob::multi\_normal\_cholesky\_log | ( | const T\_y & | *y*, |
|  |  | const T\_loc & | *mu*, |
|  |  | const Eigen::Matrix< T\_covar, Eigen::Dynamic, Eigen::Dynamic > & | *L* |
|  | ) |  |  |

The log of the multivariate normal density for the given y, mu, and a Cholesky factor L of the variance matrix.

Sigma = LL', a square, semi-positive definite matrix.

Parameters
:   |  |  |
    | --- | --- |
    | y | A scalar vector |
    | mu | The mean vector of the multivariate normal distribution. |
    | L | The Cholesky decomposition of a variance matrix of the multivariate normal distribution |

Returns
:   The log of the multivariate normal density.

Exceptions
:   |  |  |
    | --- | --- |
    | std::domain\_error | if LL' is not square, not symmetric, or not semi-positive definite. |

Template Parameters
:   |  |  |
    | --- | --- |
    | T\_y | Type of scalar. |
    | T\_loc | Type of location. |
    | T\_covar | Type of scale. |

Definition at line 53 of file multi\_normal\_cholesky.hpp.

template<typename T\_y , typename T\_loc , typename T\_covar >

|  |  |  |  |
| --- | --- | --- | --- |
| boost::math::tools::promote\_args<typename scalar\_type<T\_y>::type, typename scalar\_type<T\_loc>::type, T\_covar>::type stan::prob::multi\_normal\_cholesky\_log | ( | const T\_y & | *y*, |
|  |  | const T\_loc & | *mu*, |
|  |  | const Eigen::Matrix< T\_covar, Eigen::Dynamic, Eigen::Dynamic > & | *L* |
|  | ) |  |  |

inline

Definition at line 159 of file multi\_normal\_cholesky.hpp.

template<class RNG >

|  |  |  |  |
| --- | --- | --- | --- |
| Eigen::VectorXd stan::prob::multi\_normal\_cholesky\_rng | ( | const Eigen::Matrix< double, Eigen::Dynamic, 1 > & | *mu*, |
|  |  | const Eigen::Matrix< double, Eigen::Dynamic, Eigen::Dynamic > & | *S*, |
|  |  | RNG & | *rng* |
|  | ) |  |  |

inline

Definition at line 167 of file multi\_normal\_cholesky.hpp.

template<bool propto, typename T\_y , typename T\_loc , typename T\_covar >

|  |  |  |  |
| --- | --- | --- | --- |
| boost::math::tools::promote\_args<typename scalar\_type<T\_y>::type, typename scalar\_type<T\_loc>::type, T\_covar>::type stan::prob::multi\_normal\_log | ( | const T\_y & | *y*, |
|  |  | const T\_loc & | *mu*, |
|  |  | const Eigen::Matrix< T\_covar, Eigen::Dynamic, Eigen::Dynamic > & | *Sigma* |
|  | ) |  |  |

Definition at line 27 of file multi\_normal.hpp.

template<typename T\_y , typename T\_loc , typename T\_covar >

|  |  |  |  |
| --- | --- | --- | --- |
| boost::math::tools::promote\_args<typename scalar\_type<T\_y>::type, typename scalar\_type<T\_loc>::type, T\_covar>::type stan::prob::multi\_normal\_log | ( | const T\_y & | *y*, |
|  |  | const T\_loc & | *mu*, |
|  |  | const Eigen::Matrix< T\_covar, Eigen::Dynamic, Eigen::Dynamic > & | *Sigma* |
|  | ) |  |  |

inline

Definition at line 131 of file multi\_normal.hpp.

template<bool propto, typename T\_y , typename T\_loc , typename T\_covar >

|  |  |  |  |
| --- | --- | --- | --- |
| boost::math::tools::promote\_args<typename scalar\_type<T\_y>::type, typename scalar\_type<T\_loc>::type, T\_covar>::type stan::prob::multi\_normal\_prec\_log | ( | const T\_y & | *y*, |
|  |  | const T\_loc & | *mu*, |
|  |  | const Eigen::Matrix< T\_covar, Eigen::Dynamic, Eigen::Dynamic > & | *Sigma* |
|  | ) |  |  |

Definition at line 34 of file multi\_normal\_prec.hpp.

template<typename T\_y , typename T\_loc , typename T\_covar >

|  |  |  |  |
| --- | --- | --- | --- |
| boost::math::tools::promote\_args<typename scalar\_type<T\_y>::type, typename scalar\_type<T\_loc>::type, T\_covar>::type stan::prob::multi\_normal\_prec\_log | ( | const T\_y & | *y*, |
|  |  | const T\_loc & | *mu*, |
|  |  | const Eigen::Matrix< T\_covar, Eigen::Dynamic, Eigen::Dynamic > & | *Sigma* |
|  | ) |  |  |

inline

Definition at line 147 of file multi\_normal\_prec.hpp.

template<class RNG >

|  |  |  |  |
| --- | --- | --- | --- |
| Eigen::VectorXd stan::prob::multi\_normal\_rng | ( | const Eigen::Matrix< double, Eigen::Dynamic, 1 > & | *mu*, |
|  |  | const Eigen::Matrix< double, Eigen::Dynamic, Eigen::Dynamic > & | *S*, |
|  |  | RNG & | *rng* |
|  | ) |  |  |

inline

Definition at line 139 of file multi\_normal.hpp.

template<bool propto, typename T\_y , typename T\_dof , typename T\_loc , typename T\_scale >

|  |  |  |  |
| --- | --- | --- | --- |
| boost::math::tools::promote\_args<typename scalar\_type<T\_y>::type,T\_dof,typename scalar\_type<T\_loc>::type,T\_scale>::type stan::prob::multi\_student\_t\_log | ( | const T\_y & | *y*, |
|  |  | const T\_dof & | *nu*, |
|  |  | const T\_loc & | *mu*, |
|  |  | const Eigen::Matrix< T\_scale, Eigen::Dynamic, Eigen::Dynamic > & | *Sigma* |
|  | ) |  |  |

Return the log of the multivariate Student t distribution at the specified arguments.

Template Parameters
:   |  |  |
    | --- | --- |
    | propto | Carry out calculations up to a proportion |

Definition at line 35 of file multi\_student\_t.hpp.

template<typename T\_y , typename T\_dof , typename T\_loc , typename T\_scale >

|  |  |  |  |
| --- | --- | --- | --- |
| boost::math::tools::promote\_args<typename scalar\_type<T\_y>::type,T\_dof,typename scalar\_type<T\_loc>::type,T\_scale>::type stan::prob::multi\_student\_t\_log | ( | const T\_y & | *y*, |
|  |  | const T\_dof & | *nu*, |
|  |  | const T\_loc & | *mu*, |
|  |  | const Eigen::Matrix< T\_scale, Eigen::Dynamic, Eigen::Dynamic > & | *Sigma* |
|  | ) |  |  |

inline

Definition at line 172 of file multi\_student\_t.hpp.

template<class RNG >

|  |  |  |  |
| --- | --- | --- | --- |
| Eigen::VectorXd stan::prob::multi\_student\_t\_rng | ( | const double | *nu*, |
|  |  | const Eigen::Matrix< double, Eigen::Dynamic, 1 > & | *mu*, |
|  |  | const Eigen::Matrix< double, Eigen::Dynamic, Eigen::Dynamic > & | *s*, |
|  |  | RNG & | *rng* |
|  | ) |  |  |

inline

Definition at line 184 of file multi\_student\_t.hpp.

template<bool propto, typename T\_prob >

|  |  |  |  |
| --- | --- | --- | --- |
| boost::math::tools::promote\_args<T\_prob>::type stan::prob::multinomial\_log | ( | const std::vector< int > & | *ns*, |
|  |  | const Eigen::Matrix< T\_prob, Eigen::Dynamic, 1 > & | *theta* |
|  | ) |  |  |

Definition at line 25 of file multinomial.hpp.

template<typename T\_prob >

|  |  |  |  |
| --- | --- | --- | --- |
| boost::math::tools::promote\_args<T\_prob>::type stan::prob::multinomial\_log | ( | const std::vector< int > & | *ns*, |
|  |  | const Eigen::Matrix< T\_prob, Eigen::Dynamic, 1 > & | *theta* |
|  | ) |  |  |

Definition at line 61 of file multinomial.hpp.

template<class RNG >

|  |  |  |  |
| --- | --- | --- | --- |
| std::vector<int> stan::prob::multinomial\_rng | ( | const Eigen::Matrix< double, Eigen::Dynamic, 1 > & | *theta*, |
|  |  | const int | *N*, |
|  |  | RNG & | *rng* |
|  | ) |  |  |

inline

Definition at line 68 of file multinomial.hpp.

template<bool propto, typename T\_n , typename T\_location , typename T\_inv\_scale >

|  |  |  |  |
| --- | --- | --- | --- |
| return\_type<T\_location, T\_inv\_scale>::type stan::prob::neg\_binomial\_2\_log | ( | const T\_n & | *n*, |
|  |  | const T\_location & | *mu*, |
|  |  | const T\_inv\_scale & | *phi* |
|  | ) |  |  |

Definition at line 31 of file neg\_binomial\_2.hpp.

template<typename T\_n , typename T\_location , typename T\_inv\_scale >

|  |  |  |  |
| --- | --- | --- | --- |
| return\_type<T\_location, T\_inv\_scale>::type stan::prob::neg\_binomial\_2\_log | ( | const T\_n & | *n*, |
|  |  | const T\_location & | *mu*, |
|  |  | const T\_inv\_scale & | *phi* |
|  | ) |  |  |

inline

Definition at line 138 of file neg\_binomial\_2.hpp.

template<bool propto, typename T\_n , typename T\_log\_location , typename T\_inv\_scale >

|  |  |  |  |
| --- | --- | --- | --- |
| return\_type<T\_log\_location, T\_inv\_scale>::type stan::prob::neg\_binomial\_2\_log\_log | ( | const T\_n & | *n*, |
|  |  | const T\_log\_location & | *eta*, |
|  |  | const T\_inv\_scale & | *phi* |
|  | ) |  |  |

Definition at line 150 of file neg\_binomial\_2.hpp.

template<typename T\_n , typename T\_log\_location , typename T\_inv\_scale >

|  |  |  |  |
| --- | --- | --- | --- |
| return\_type<T\_log\_location, T\_inv\_scale>::type stan::prob::neg\_binomial\_2\_log\_log | ( | const T\_n & | *n*, |
|  |  | const T\_log\_location & | *eta*, |
|  |  | const T\_inv\_scale & | *phi* |
|  | ) |  |  |

inline

Definition at line 258 of file neg\_binomial\_2.hpp.

template<class RNG >

|  |  |  |  |
| --- | --- | --- | --- |
| int stan::prob::neg\_binomial\_2\_log\_rng | ( | const double | *eta*, |
|  |  | const double | *phi*, |
|  |  | RNG & | *rng* |
|  | ) |  |  |

inline

Definition at line 286 of file neg\_binomial\_2.hpp.

template<class RNG >

|  |  |  |  |
| --- | --- | --- | --- |
| int stan::prob::neg\_binomial\_2\_rng | ( | const double | *mu*, |
|  |  | const double | *phi*, |
|  |  | RNG & | *rng* |
|  | ) |  |  |

inline

Definition at line 266 of file neg\_binomial\_2.hpp.

template<typename T\_n , typename T\_shape , typename T\_inv\_scale >

|  |  |  |  |
| --- | --- | --- | --- |
| return\_type<T\_shape, T\_inv\_scale>::type stan::prob::neg\_binomial\_ccdf\_log | ( | const T\_n & | *n*, |
|  |  | const T\_shape & | *alpha*, |
|  |  | const T\_inv\_scale & | *beta* |
|  | ) |  |  |

Definition at line 428 of file neg\_binomial.hpp.

template<typename T\_n , typename T\_shape , typename T\_inv\_scale >

|  |  |  |  |
| --- | --- | --- | --- |
| return\_type<T\_shape, T\_inv\_scale>::type stan::prob::neg\_binomial\_cdf | ( | const T\_n & | *n*, |
|  |  | const T\_shape & | *alpha*, |
|  |  | const T\_inv\_scale & | *beta* |
|  | ) |  |  |

Definition at line 181 of file neg\_binomial.hpp.

template<typename T\_n , typename T\_shape , typename T\_inv\_scale >

|  |  |  |  |
| --- | --- | --- | --- |
| return\_type<T\_shape, T\_inv\_scale>::type stan::prob::neg\_binomial\_cdf\_log | ( | const T\_n & | *n*, |
|  |  | const T\_shape & | *alpha*, |
|  |  | const T\_inv\_scale & | *beta* |
|  | ) |  |  |

Definition at line 314 of file neg\_binomial.hpp.

template<bool propto, typename T\_n , typename T\_shape , typename T\_inv\_scale >

|  |  |  |  |
| --- | --- | --- | --- |
| return\_type<T\_shape, T\_inv\_scale>::type stan::prob::neg\_binomial\_log | ( | const T\_n & | *n*, |
|  |  | const T\_shape & | *alpha*, |
|  |  | const T\_inv\_scale & | *beta* |
|  | ) |  |  |

Definition at line 30 of file neg\_binomial.hpp.

template<typename T\_n , typename T\_shape , typename T\_inv\_scale >

|  |  |  |  |
| --- | --- | --- | --- |
| return\_type<T\_shape, T\_inv\_scale>::type stan::prob::neg\_binomial\_log | ( | const T\_n & | *n*, |
|  |  | const T\_shape & | *alpha*, |
|  |  | const T\_inv\_scale & | *beta* |
|  | ) |  |  |

inline

Definition at line 171 of file neg\_binomial.hpp.

template<class RNG >

|  |  |  |  |
| --- | --- | --- | --- |
| int stan::prob::neg\_binomial\_rng | ( | const double | *alpha*, |
|  |  | const double | *beta*, |
|  |  | RNG & | *rng* |
|  | ) |  |  |

inline

Definition at line 541 of file neg\_binomial.hpp.

template<typename T\_y , typename T\_loc , typename T\_scale >

|  |  |  |  |
| --- | --- | --- | --- |
| return\_type<T\_y,T\_loc,T\_scale>::type stan::prob::normal\_ccdf\_log | ( | const T\_y & | *y*, |
|  |  | const T\_loc & | *mu*, |
|  |  | const T\_scale & | *sigma* |
|  | ) |  |  |

Definition at line 305 of file normal.hpp.

template<typename T\_y , typename T\_loc , typename T\_scale >

|  |  |  |  |
| --- | --- | --- | --- |
| return\_type<T\_y,T\_loc,T\_scale>::type stan::prob::normal\_cdf | ( | const T\_y & | *y*, |
|  |  | const T\_loc & | *mu*, |
|  |  | const T\_scale & | *sigma* |
|  | ) |  |  |

Calculates the normal cumulative distribution function for the given variate, location, and scale.

.

Errors are configured by policy. All variables must be finite and the scale must be strictly greater than zero.

Parameters
:   |  |  |
    | --- | --- |
    | y | A scalar variate. |
    | mu | The location of the normal distribution. |
    | sigma | The scale of the normal distriubtion |

Returns
:   The unit normal cdf evaluated at the specified arguments.

Template Parameters
:   |  |  |
    | --- | --- |
    | T\_y | Type of y. |
    | T\_loc | Type of mean parameter. |
    | T\_scale | Type of standard deviation paramater. |
    | Policy | Error-handling policy. |

Definition at line 152 of file normal.hpp.

template<typename T\_y , typename T\_loc , typename T\_scale >

|  |  |  |  |
| --- | --- | --- | --- |
| return\_type<T\_y,T\_loc,T\_scale>::type stan::prob::normal\_cdf\_log | ( | const T\_y & | *y*, |
|  |  | const T\_loc & | *mu*, |
|  |  | const T\_scale & | *sigma* |
|  | ) |  |  |

Definition at line 233 of file normal.hpp.

template<bool propto, typename T\_y , typename T\_loc , typename T\_scale >

|  |  |  |  |
| --- | --- | --- | --- |
| boost::enable\_if\_c<contains\_fvar<T\_y,T\_loc,T\_scale>::value, typename return\_type<T\_y,T\_loc,T\_scale>::type>::type stan::prob::normal\_log | ( | const T\_y & | *y*, |
|  |  | const T\_loc & | *mu*, |
|  |  | const T\_scale & | *sigma* |
|  | ) |  |  |

Definition at line 22 of file normal.hpp.

template<bool propto, typename T\_y , typename T\_loc , typename T\_scale >

|  |  |  |  |
| --- | --- | --- | --- |
| boost::enable\_if\_c<is\_var\_or\_arithmetic<T\_y,T\_loc,T\_scale>::value, typename return\_type<T\_y,T\_loc,T\_scale>::type>::type stan::prob::normal\_log | ( | const T\_y & | *y*, |
|  |  | const T\_loc & | *mu*, |
|  |  | const T\_scale & | *sigma* |
|  | ) |  |  |

The log of the normal density for the specified scalar(s) given the specified mean(s) and deviation(s).

y, mu, or sigma can each be either a scalar or a std::vector. Any vector inputs must be the same length.

The result log probability is defined to be the sum of the log probabilities for each observation/mean/deviation triple.

Parameters
:   |  |  |
    | --- | --- |
    | y | (Sequence of) scalar(s). |
    | mu | (Sequence of) location parameter(s) for the normal distribution. |
    | sigma | (Sequence of) scale parameters for the normal distribution. |

Returns
:   The log of the product of the densities.

Exceptions
:   |  |  |
    | --- | --- |
    | std::domain\_error | if the scale is not positive. |

Template Parameters
:   |  |  |
    | --- | --- |
    | T\_y | Underlying type of scalar in sequence. |
    | T\_loc | Type of location parameter. |

Definition at line 41 of file normal.hpp.

template<typename T\_y , typename T\_loc , typename T\_scale >

|  |  |  |  |
| --- | --- | --- | --- |
| return\_type<T\_y,T\_loc,T\_scale>::type stan::prob::normal\_log | ( | const T\_y & | *y*, |
|  |  | const T\_loc & | *mu*, |
|  |  | const T\_scale & | *sigma* |
|  | ) |  |  |

inline

Definition at line 128 of file normal.hpp.

template<class RNG >

|  |  |  |  |
| --- | --- | --- | --- |
| double stan::prob::normal\_rng | ( | const double | *mu*, |
|  |  | const double | *sigma*, |
|  |  | RNG & | *rng* |
|  | ) |  |  |

inline

Definition at line 377 of file normal.hpp.

template<typename T >

|  |  |  |  |  |  |
| --- | --- | --- | --- | --- | --- |
| Eigen::Matrix<T,Eigen::Dynamic,1> stan::prob::ordered\_constrain | ( | const Eigen::Matrix< T, Eigen::Dynamic, 1 > & | *x* | ) |  |

Return an increasing ordered vector derived from the specified free vector.

The returned constrained vector will have the same dimensionality as the specified free vector.

Parameters
:   |  |  |
    | --- | --- |
    | x | Free vector of scalars. |

Returns
:   Positive, increasing ordered vector.

Template Parameters
:   |  |  |
    | --- | --- |
    | T | Type of scalar. |

Definition at line 1224 of file transform.hpp.

template<typename T >

|  |  |  |  |
| --- | --- | --- | --- |
| Eigen::Matrix<T,Eigen::Dynamic,1> stan::prob::ordered\_constrain | ( | const Eigen::Matrix< T, Eigen::Dynamic, 1 > & | *x*, |
|  |  | T & | *lp* |
|  | ) |  |  |

inline

Return a positive valued, increasing ordered vector derived from the specified free vector and increment the specified log probability reference with the log absolute Jacobian determinant of the transform.

The returned constrained vector will have the same dimensionality as the specified free vector.

Parameters
:   |  |  |
    | --- | --- |
    | x | Free vector of scalars. |
    | lp | Log probability reference. |

Returns
:   Positive, increasing ordered vector.

Template Parameters
:   |  |  |
    | --- | --- |
    | T | Type of scalar. |

Definition at line 1256 of file transform.hpp.

template<typename T >

|  |  |  |  |  |  |
| --- | --- | --- | --- | --- | --- |
| Eigen::Matrix<T,Eigen::Dynamic,1> stan::prob::ordered\_free | ( | const Eigen::Matrix< T, Eigen::Dynamic, 1 > & | *y* | ) |  |

Return the vector of unconstrained scalars that transform to the specified positive ordered vector.

This function inverts the constraining operation defined in `ordered_constrain(Matrix)`,

Parameters
:   |  |  |
    | --- | --- |
    | y | Vector of positive, ordered scalars. |

Returns
:   Free vector that transforms into the input vector.

Template Parameters
:   |  |  |
    | --- | --- |
    | T | Type of scalar. |

Exceptions
:   |  |  |
    | --- | --- |
    | std::domain\_error | if y is not a vector of positive, ordered scalars. |

Definition at line 1285 of file transform.hpp.

template<bool propto, typename T\_lambda , typename T\_cut >

|  |  |  |  |
| --- | --- | --- | --- |
| boost::math::tools::promote\_args<T\_lambda,T\_cut>::type stan::prob::ordered\_logistic\_log | ( | int | *y*, |
|  |  | const T\_lambda & | *lambda*, |
|  |  | const Eigen::Matrix< T\_cut, Eigen::Dynamic, 1 > & | *c* |
|  | ) |  |  |

Returns the (natural) log probability of the specified integer outcome given the continuous location and specified cutpoints in an ordered logistic model.

Typically the continous location will be the dot product of a vector of regression coefficients and a vector of predictors for the outcome.

Template Parameters
:   |  |  |
    | --- | --- |
    | propto | True if calculating up to a proportion. |
    | T\_loc | Location type. |
    | T\_cut | Cut-point type. |
    | Policy | Error policy (only its type matters). |

Parameters
:   |  |  |
    | --- | --- |
    | y | Outcome. |
    | lambda | Location. |
    | c | Positive increasing vector of cutpoints. |

Returns
:   Log probability of outcome given location and cutpoints.

Exceptions
:   |  |  |
    | --- | --- |
    | std::domain\_error | If the outcome is not between 1 and the number of cutpoints plus 2; if the cutpoint vector is empty; if the cutpoint vector contains a non-positive, non-finite value; or if the cutpoint vector is not sorted in ascending order. |

Definition at line 60 of file ordered\_logistic.hpp.

template<typename T\_lambda , typename T\_cut >

|  |  |  |  |
| --- | --- | --- | --- |
| boost::math::tools::promote\_args<T\_lambda,T\_cut>::type stan::prob::ordered\_logistic\_log | ( | int | *y*, |
|  |  | const T\_lambda & | *lambda*, |
|  |  | const Eigen::Matrix< T\_cut, Eigen::Dynamic, 1 > & | *c* |
|  | ) |  |  |

Definition at line 108 of file ordered\_logistic.hpp.

template<class RNG >

|  |  |  |  |
| --- | --- | --- | --- |
| int stan::prob::ordered\_logistic\_rng | ( | const double | *eta*, |
|  |  | const Eigen::Matrix< double, Eigen::Dynamic, 1 > & | *c*, |
|  |  | RNG & | *rng* |
|  | ) |  |  |

inline

Definition at line 115 of file ordered\_logistic.hpp.

template<typename T\_y , typename T\_scale , typename T\_shape >

|  |  |  |  |
| --- | --- | --- | --- |
| return\_type<T\_y, T\_scale, T\_shape>::type stan::prob::pareto\_ccdf\_log | ( | const T\_y & | *y*, |
|  |  | const T\_scale & | *y\_min*, |
|  |  | const T\_shape & | *alpha* |
|  | ) |  |  |

Definition at line 303 of file pareto.hpp.

template<typename T\_y , typename T\_scale , typename T\_shape >

|  |  |  |  |
| --- | --- | --- | --- |
| return\_type<T\_y, T\_scale, T\_shape>::type stan::prob::pareto\_cdf | ( | const T\_y & | *y*, |
|  |  | const T\_scale & | *y\_min*, |
|  |  | const T\_shape & | *alpha* |
|  | ) |  |  |

Definition at line 128 of file pareto.hpp.

template<typename T\_y , typename T\_scale , typename T\_shape >

|  |  |  |  |
| --- | --- | --- | --- |
| return\_type<T\_y, T\_scale, T\_shape>::type stan::prob::pareto\_cdf\_log | ( | const T\_y & | *y*, |
|  |  | const T\_scale & | *y\_min*, |
|  |  | const T\_shape & | *alpha* |
|  | ) |  |  |

Definition at line 223 of file pareto.hpp.

template<bool propto, typename T\_y , typename T\_scale , typename T\_shape >

|  |  |  |  |
| --- | --- | --- | --- |
| return\_type<T\_y,T\_scale,T\_shape>::type stan::prob::pareto\_log | ( | const T\_y & | *y*, |
|  |  | const T\_scale & | *y\_min*, |
|  |  | const T\_shape & | *alpha* |
|  | ) |  |  |

Definition at line 24 of file pareto.hpp.

template<typename T\_y , typename T\_scale , typename T\_shape >

|  |  |  |  |
| --- | --- | --- | --- |
| return\_type<T\_y,T\_scale,T\_shape>::type stan::prob::pareto\_log | ( | const T\_y & | *y*, |
|  |  | const T\_scale & | *y\_min*, |
|  |  | const T\_shape & | *alpha* |
|  | ) |  |  |

inline

Definition at line 122 of file pareto.hpp.

template<class RNG >

|  |  |  |  |
| --- | --- | --- | --- |
| double stan::prob::pareto\_rng | ( | const double | *y\_min*, |
|  |  | const double | *alpha*, |
|  |  | RNG & | *rng* |
|  | ) |  |  |

inline

Definition at line 379 of file pareto.hpp.

template<typename T\_y , typename T\_loc , typename T\_scale , typename T\_shape >

|  |  |  |  |
| --- | --- | --- | --- |
| return\_type<T\_y, T\_loc, T\_scale, T\_shape>::type stan::prob::pareto\_type\_2\_ccdf\_log | ( | const T\_y & | *y*, |
|  |  | const T\_loc & | *mu*, |
|  |  | const T\_scale & | *lambda*, |
|  |  | const T\_shape & | *alpha* |
|  | ) |  |  |

Definition at line 381 of file pareto\_type\_2.hpp.

template<typename T\_y , typename T\_loc , typename T\_scale , typename T\_shape >

|  |  |  |  |
| --- | --- | --- | --- |
| return\_type<T\_y, T\_loc, T\_scale, T\_shape>::type stan::prob::pareto\_type\_2\_cdf | ( | const T\_y & | *y*, |
|  |  | const T\_loc & | *mu*, |
|  |  | const T\_scale & | *lambda*, |
|  |  | const T\_shape & | *alpha* |
|  | ) |  |  |

Definition at line 140 of file pareto\_type\_2.hpp.

template<typename T\_y , typename T\_loc , typename T\_scale , typename T\_shape >

|  |  |  |  |
| --- | --- | --- | --- |
| return\_type<T\_y, T\_loc, T\_scale, T\_shape>::type stan::prob::pareto\_type\_2\_cdf\_log | ( | const T\_y & | *y*, |
|  |  | const T\_loc & | *mu*, |
|  |  | const T\_scale & | *lambda*, |
|  |  | const T\_shape & | *alpha* |
|  | ) |  |  |

Definition at line 270 of file pareto\_type\_2.hpp.

template<bool propto, typename T\_y , typename T\_loc , typename T\_scale , typename T\_shape >

|  |  |  |  |
| --- | --- | --- | --- |
| return\_type<T\_y,T\_loc,T\_scale,T\_shape>::type stan::prob::pareto\_type\_2\_log | ( | const T\_y & | *y*, |
|  |  | const T\_loc & | *mu*, |
|  |  | const T\_scale & | *lambda*, |
|  |  | const T\_shape & | *alpha* |
|  | ) |  |  |

Definition at line 24 of file pareto\_type\_2.hpp.

template<typename T\_y , typename T\_loc , typename T\_scale , typename T\_shape >

|  |  |  |  |
| --- | --- | --- | --- |
| return\_type<T\_y,T\_loc,T\_scale,T\_shape>::type stan::prob::pareto\_type\_2\_log | ( | const T\_y & | *y*, |
|  |  | const T\_loc & | *mu*, |
|  |  | const T\_scale & | *lambda*, |
|  |  | const T\_shape & | *alpha* |
|  | ) |  |  |

inline

Definition at line 133 of file pareto\_type\_2.hpp.

template<class RNG >

|  |  |  |  |
| --- | --- | --- | --- |
| double stan::prob::pareto\_type\_2\_rng | ( | const double | *mu*, |
|  |  | const double | *lambda*, |
|  |  | const double | *alpha*, |
|  |  | RNG & | *rng* |
|  | ) |  |  |

inline

Definition at line 495 of file pareto\_type\_2.hpp.

template<typename T\_n , typename T\_rate >

|  |  |  |  |
| --- | --- | --- | --- |
| return\_type<T\_rate>::type stan::prob::poisson\_ccdf\_log | ( | const T\_n & | *n*, |
|  |  | const T\_rate & | *lambda* |
|  | ) |  |  |

Definition at line 322 of file poisson.hpp.

template<typename T\_n , typename T\_rate >

|  |  |  |  |
| --- | --- | --- | --- |
| return\_type<T\_rate>::type stan::prob::poisson\_cdf | ( | const T\_n & | *n*, |
|  |  | const T\_rate & | *lambda* |
|  | ) |  |  |

Definition at line 194 of file poisson.hpp.

template<typename T\_n , typename T\_rate >

|  |  |  |  |
| --- | --- | --- | --- |
| return\_type<T\_rate>::type stan::prob::poisson\_cdf\_log | ( | const T\_n & | *n*, |
|  |  | const T\_rate & | *lambda* |
|  | ) |  |  |

Definition at line 260 of file poisson.hpp.

template<bool propto, typename T\_n , typename T\_rate >

|  |  |  |  |
| --- | --- | --- | --- |
| return\_type<T\_rate>::type stan::prob::poisson\_log | ( | const T\_n & | *n*, |
|  |  | const T\_rate & | *lambda* |
|  | ) |  |  |

Definition at line 27 of file poisson.hpp.

template<typename T\_n , typename T\_rate >

|  |  |  |  |
| --- | --- | --- | --- |
| return\_type<T\_rate>::type stan::prob::poisson\_log | ( | const T\_n & | *n*, |
|  |  | const T\_rate & | *lambda* |
|  | ) |  |  |

inline

Definition at line 101 of file poisson.hpp.

template<bool propto, typename T\_n , typename T\_log\_rate >

|  |  |  |  |
| --- | --- | --- | --- |
| return\_type<T\_log\_rate>::type stan::prob::poisson\_log\_log | ( | const T\_n & | *n*, |
|  |  | const T\_log\_rate & | *alpha* |
|  | ) |  |  |

Definition at line 109 of file poisson.hpp.

template<typename T\_n , typename T\_log\_rate >

|  |  |  |  |
| --- | --- | --- | --- |
| return\_type<T\_log\_rate>::type stan::prob::poisson\_log\_log | ( | const T\_n & | *n*, |
|  |  | const T\_log\_rate & | *alpha* |
|  | ) |  |  |

inline

Definition at line 187 of file poisson.hpp.

template<class RNG >

|  |  |  |  |
| --- | --- | --- | --- |
| int stan::prob::poisson\_rng | ( | const double | *lambda*, |
|  |  | RNG & | *rng* |
|  | ) |  |  |

inline

Definition at line 384 of file poisson.hpp.

template<typename T >

|  |  |  |  |  |  |
| --- | --- | --- | --- | --- | --- |
| T stan::prob::positive\_constrain | ( | const T | *x* | ) |  |

inline

Return the positive value for the specified unconstrained input.

The transform applied is

.

Parameters
:   |  |  |
    | --- | --- |
    | x | Arbitrary input scalar. |

Returns
:   Input transformed to be positive.

Definition at line 446 of file transform.hpp.

template<typename T >

|  |  |  |  |
| --- | --- | --- | --- |
| T stan::prob::positive\_constrain | ( | const T | *x*, |
|  |  | T & | *lp* |
|  | ) |  |  |

inline

Return the positive value for the specified unconstrained input, incrementing the scalar reference with the log absolute Jacobian determinant.

See `positive_constrain(T)` for details of the transform. The log absolute Jacobian determinant is

.

Parameters
:   |  |  |
    | --- | --- |
    | x | Arbitrary input scalar. |
    | lp | Log probability reference. |

Returns
:   Input transformed to be positive.

Template Parameters
:   |  |  |
    | --- | --- |
    | T | Type of scalar. |

Definition at line 468 of file transform.hpp.

template<typename T >

|  |  |  |  |  |  |
| --- | --- | --- | --- | --- | --- |
| T stan::prob::positive\_free | ( | const T | *y* | ) |  |

inline

Return the unconstrained value corresponding to the specified positive-constrained value.

The transform is the inverse of the transform  applied by `positive_constrain(T)`, namely

.

The input is validated using `stan::math::check_positive()`.

Parameters
:   |  |  |
    | --- | --- |
    | y | Input scalar. |

Returns
:   Unconstrained value that produces the input when constrained.

Template Parameters
:   |  |  |
    | --- | --- |
    | T | Type of scalar. |

Exceptions
:   |  |  |
    | --- | --- |
    | std::domain\_error | if the variable is negative. |

Definition at line 491 of file transform.hpp.

template<typename T >

|  |  |  |  |  |  |
| --- | --- | --- | --- | --- | --- |
| Eigen::Matrix<T,Eigen::Dynamic,1> stan::prob::positive\_ordered\_constrain | ( | const Eigen::Matrix< T, Eigen::Dynamic, 1 > & | *x* | ) |  |

Return an increasing positive ordered vector derived from the specified free vector.

The returned constrained vector will have the same dimensionality as the specified free vector.

Parameters
:   |  |  |
    | --- | --- |
    | x | Free vector of scalars. |

Returns
:   Positive, increasing ordered vector.

Template Parameters
:   |  |  |
    | --- | --- |
    | T | Type of scalar. |

Definition at line 1317 of file transform.hpp.

template<typename T >

|  |  |  |  |
| --- | --- | --- | --- |
| Eigen::Matrix<T,Eigen::Dynamic,1> stan::prob::positive\_ordered\_constrain | ( | const Eigen::Matrix< T, Eigen::Dynamic, 1 > & | *x*, |
|  |  | T & | *lp* |
|  | ) |  |  |

inline

Return a positive valued, increasing positive ordered vector derived from the specified free vector and increment the specified log probability reference with the log absolute Jacobian determinant of the transform.

The returned constrained vector will have the same dimensionality as the specified free vector.

Parameters
:   |  |  |
    | --- | --- |
    | x | Free vector of scalars. |
    | lp | Log probability reference. |

Returns
:   Positive, increasing ordered vector.

Template Parameters
:   |  |  |
    | --- | --- |
    | T | Type of scalar. |

Definition at line 1348 of file transform.hpp.

template<typename T >

|  |  |  |  |  |  |
| --- | --- | --- | --- | --- | --- |
| Eigen::Matrix<T,Eigen::Dynamic,1> stan::prob::positive\_ordered\_free | ( | const Eigen::Matrix< T, Eigen::Dynamic, 1 > & | *y* | ) |  |

Return the vector of unconstrained scalars that transform to the specified positive ordered vector.

This function inverts the constraining operation defined in `positive_ordered_constrain(Matrix)`,

Parameters
:   |  |  |
    | --- | --- |
    | y | Vector of positive, ordered scalars. |

Returns
:   Free vector that transforms into the input vector.

Template Parameters
:   |  |  |
    | --- | --- |
    | T | Type of scalar. |

Exceptions
:   |  |  |
    | --- | --- |
    | std::domain\_error | if y is not a vector of positive, ordered scalars. |

Definition at line 1376 of file transform.hpp.

template<typename T >

|  |  |  |  |  |  |
| --- | --- | --- | --- | --- | --- |
| T stan::prob::prob\_constrain | ( | const T | *x* | ) |  |

inline

Return a probability value constrained to fall between 0 and 1 (inclusive) for the specified free scalar.

The transform is the inverse logit,

.

Parameters
:   |  |  |
    | --- | --- |
    | x | Free scalar. |

Returns
:   Probability-constrained result of transforming the free scalar.

Template Parameters
:   |  |  |
    | --- | --- |
    | T | Type of scalar. |

Definition at line 875 of file transform.hpp.

template<typename T >

|  |  |  |  |
| --- | --- | --- | --- |
| T stan::prob::prob\_constrain | ( | const T | *x*, |
|  |  | T & | *lp* |
|  | ) |  |  |

inline

Return a probability value constrained to fall between 0 and 1 (inclusive) for the specified free scalar and increment the specified log probability reference with the log absolute Jacobian determinant of the transform.

The transform is as defined for `prob_constrain(T)`. The log absolute Jacobian determinant is

The log absolute Jacobian determinant is

.

Parameters
:   |  |  |
    | --- | --- |
    | x | Free scalar. |
    | lp | Log probability reference. |

Returns
:   Probability-constrained result of transforming the free scalar.

Template Parameters
:   |  |  |
    | --- | --- |
    | T | Type of scalar. |

Definition at line 903 of file transform.hpp.

template<typename T >

|  |  |  |  |  |  |
| --- | --- | --- | --- | --- | --- |
| T stan::prob::prob\_free | ( | const T | *y* | ) |  |

inline

Return the free scalar that when transformed to a probability produces the specified scalar.

The function that reverses the constraining transform specified in `prob_constrain(T)` is the logit function,

.

Parameters
:   |  |  |
    | --- | --- |
    | y | Scalar input. |

Template Parameters
:   |  |  |
    | --- | --- |
    | T | Type of scalar. |

Exceptions
:   |  |  |
    | --- | --- |
    | std::domain\_error | if y is less than 0 or greater than 1. |

Definition at line 927 of file transform.hpp.

template<typename T\_y , typename T\_scale >

|  |  |  |  |
| --- | --- | --- | --- |
| return\_type<T\_y,T\_scale>::type stan::prob::rayleigh\_ccdf\_log | ( | const T\_y & | *y*, |
|  |  | const T\_scale & | *sigma* |
|  | ) |  |  |

Definition at line 239 of file rayleigh.hpp.

template<typename T\_y , typename T\_scale >

|  |  |  |  |
| --- | --- | --- | --- |
| return\_type<T\_y,T\_scale>::type stan::prob::rayleigh\_cdf | ( | const T\_y & | *y*, |
|  |  | const T\_scale & | *sigma* |
|  | ) |  |  |

Definition at line 105 of file rayleigh.hpp.

template<typename T\_y , typename T\_scale >

|  |  |  |  |
| --- | --- | --- | --- |
| return\_type<T\_y,T\_scale>::type stan::prob::rayleigh\_cdf\_log | ( | const T\_y & | *y*, |
|  |  | const T\_scale & | *sigma* |
|  | ) |  |  |

Definition at line 176 of file rayleigh.hpp.

template<bool propto, typename T\_y , typename T\_scale >

|  |  |  |  |
| --- | --- | --- | --- |
| return\_type<T\_y,T\_scale>::type stan::prob::rayleigh\_log | ( | const T\_y & | *y*, |
|  |  | const T\_scale & | *sigma* |
|  | ) |  |  |

Definition at line 21 of file rayleigh.hpp.

template<typename T\_y , typename T\_scale >

|  |  |  |  |
| --- | --- | --- | --- |
| return\_type<T\_y,T\_scale>::type stan::prob::rayleigh\_log | ( | const T\_y & | *y*, |
|  |  | const T\_scale & | *sigma* |
|  | ) |  |  |

inline

Definition at line 99 of file rayleigh.hpp.

template<class RNG >

|  |  |  |  |
| --- | --- | --- | --- |
| double stan::prob::rayleigh\_rng | ( | const double | *sigma*, |
|  |  | RNG & | *rng* |
|  | ) |  |  |

inline

Definition at line 299 of file rayleigh.hpp.

template<typename T >

|  |  |  |  |
| --- | --- | --- | --- |
| Eigen::Matrix<T,Eigen::Dynamic,Eigen::Dynamic> stan::prob::read\_corr\_L | ( | const Eigen::Array< T, Eigen::Dynamic, 1 > & | *CPCs*, |
|  |  | const size\_t | *K* |
|  | ) |  |  |

Return the Cholesky factor of the correlation matrix of the specified dimensionality corresponding to the specified canonical partial correlations.

It is generally better to work with the Cholesky factor rather than the correlation matrix itself when the determinant, inverse, etc. of the correlation matrix is needed for some statistical calculation.

See `read_corr_matrix(Array,size_t,T)` for more information.

Parameters
:   |  |  |
    | --- | --- |
    | CPCs | The (K choose 2) canonical partial correlations in (-1,1). |
    | K | Dimensionality of correlation matrix. |

Returns
:   Cholesky factor of correlation matrix for specified canonical partial correlations.

Template Parameters
:   |  |  |
    | --- | --- |
    | T | Type of underlying scalar. |

Definition at line 139 of file transform.hpp.

template<typename T >

|  |  |  |  |
| --- | --- | --- | --- |
| Eigen::Matrix<T,Eigen::Dynamic,Eigen::Dynamic> stan::prob::read\_corr\_L | ( | const Eigen::Array< T, Eigen::Dynamic, 1 > & | *CPCs*, |
|  |  | const size\_t | *K*, |
|  |  | T & | *log\_prob* |
|  | ) |  |  |

Return the Cholesky factor of the correlation matrix of the specified dimensionality corresponding to the specified canonical partial correlations, incrementing the specified scalar reference with the log absolute determinant of the Jacobian of the transformation.

The implementation is Ben Goodrich's Cholesky factor-based approach to the C-vine method of:

- Daniel Lewandowski, Dorota Kurowicka, and Harry Joe, Generating random correlation matrices based on vines and extended onion method Journal of Multivariate Analysis 100 (2009) 1989–2001

Parameters
:   |  |  |
    | --- | --- |
    | CPCs | The (K choose 2) canonical partial correlations in (-1,1). |
    | K | Dimensionality of correlation matrix. |
    | log\_prob | Reference to variable to increment with the log Jacobian determinant. |

Returns
:   Cholesky factor of correlation matrix for specified partial correlations.

Template Parameters
:   |  |  |
    | --- | --- |
    | T | Type of underlying scalar. |

Definition at line 215 of file transform.hpp.

template<typename T >

|  |  |  |  |
| --- | --- | --- | --- |
| Eigen::Matrix<T,Eigen::Dynamic,Eigen::Dynamic> stan::prob::read\_corr\_matrix | ( | const Eigen::Array< T, Eigen::Dynamic, 1 > & | *CPCs*, |
|  |  | const size\_t | *K* |
|  | ) |  |  |

Return the correlation matrix of the specified dimensionality corresponding to the specified canonical partial correlations.

See `read_corr_matrix(Array,size_t,T)` for more information.

Parameters
:   |  |  |
    | --- | --- |
    | CPCs | The (K choose 2) canonical partial correlations in (-1,1). |
    | K | Dimensionality of correlation matrix. |

Returns
:   Cholesky factor of correlation matrix for specified canonical partial correlations.

Template Parameters
:   |  |  |
    | --- | --- |
    | T | Type of underlying scalar. |

Definition at line 181 of file transform.hpp.

template<typename T >

|  |  |  |  |
| --- | --- | --- | --- |
| Eigen::Matrix<T,Eigen::Dynamic,Eigen::Dynamic> stan::prob::read\_corr\_matrix | ( | const Eigen::Array< T, Eigen::Dynamic, 1 > & | *CPCs*, |
|  |  | const size\_t | *K*, |
|  |  | T & | *log\_prob* |
|  | ) |  |  |

Return the correlation matrix of the specified dimensionality corresponding to the specified canonical partial correlations, incrementing the specified scalar reference with the log absolute determinant of the Jacobian of the transformation.

It is usually preferable to utilize the version that returns the Cholesky factor of the correlation matrix rather than the correlation matrix itself in statistical calculations.

Parameters
:   |  |  |
    | --- | --- |
    | CPCs | The (K choose 2) canonical partial correlations in (-1,1). |
    | K | Dimensionality of correlation matrix. |
    | log\_prob | Reference to variable to increment with the log Jacobian determinant. |

Returns
:   Correlation matrix for specified partial correlations.

Template Parameters
:   |  |  |
    | --- | --- |
    | T | Type of underlying scalar. |

Definition at line 258 of file transform.hpp.

template<typename T >

|  |  |  |  |
| --- | --- | --- | --- |
| Eigen::Matrix<T,Eigen::Dynamic,Eigen::Dynamic> stan::prob::read\_cov\_L | ( | const Eigen::Array< T, Eigen::Dynamic, 1 > & | *CPCs*, |
|  |  | const Eigen::Array< T, Eigen::Dynamic, 1 > & | *sds*, |
|  |  | T & | *log\_prob* |
|  | ) |  |  |

This is the function that should be called prior to evaluating the density of any elliptical distribution.

Parameters
:   |  |  |
    | --- | --- |
    | CPCs | on (-1,1) |
    | sds | on (0,inf) |
    | log\_prob | the log probability value to increment with the Jacobian |

Returns
:   Cholesky factor of covariance matrix for specified partial correlations.

Definition at line 280 of file transform.hpp.

template<typename T >

|  |  |  |  |
| --- | --- | --- | --- |
| Eigen::Matrix<T,Eigen::Dynamic,Eigen::Dynamic> stan::prob::read\_cov\_matrix | ( | const Eigen::Array< T, Eigen::Dynamic, 1 > & | *CPCs*, |
|  |  | const Eigen::Array< T, Eigen::Dynamic, 1 > & | *sds*, |
|  |  | T & | *log\_prob* |
|  | ) |  |  |

A generally worse alternative to call prior to evaluating the density of an elliptical distribution.

Parameters
:   |  |  |
    | --- | --- |
    | CPCs | on (-1,1) |
    | sds | on (0,inf) |
    | log\_prob | the log probability value to increment with the Jacobian |

Returns
:   Covariance matrix for specified partial correlations.

Definition at line 300 of file transform.hpp.

template<typename T >

|  |  |  |  |
| --- | --- | --- | --- |
| Eigen::Matrix<T,Eigen::Dynamic,Eigen::Dynamic> stan::prob::read\_cov\_matrix | ( | const Eigen::Array< T, Eigen::Dynamic, 1 > & | *CPCs*, |
|  |  | const Eigen::Array< T, Eigen::Dynamic, 1 > & | *sds* |
|  | ) |  |  |

Builds a covariance matrix from CPCs and standard deviations.

Parameters
:   |  |  |
    | --- | --- |
    | CPCs | in (-1,1) |
    | sds | in (0,inf) |

Definition at line 319 of file transform.hpp.

template<typename T\_y , typename T\_dof , typename T\_scale >

|  |  |  |  |
| --- | --- | --- | --- |
| return\_type<T\_y, T\_dof, T\_scale>::type stan::prob::scaled\_inv\_chi\_square\_ccdf\_log | ( | const T\_y & | *y*, |
|  |  | const T\_dof & | *nu*, |
|  |  | const T\_scale & | *s* |
|  | ) |  |  |

Definition at line 419 of file scaled\_inv\_chi\_square.hpp.

template<typename T\_y , typename T\_dof , typename T\_scale >

|  |  |  |  |
| --- | --- | --- | --- |
| return\_type<T\_y, T\_dof, T\_scale>::type stan::prob::scaled\_inv\_chi\_square\_cdf | ( | const T\_y & | *y*, |
|  |  | const T\_dof & | *nu*, |
|  |  | const T\_scale & | *s* |
|  | ) |  |  |

The CDF of a scaled inverse chi-squared density for y with the specified degrees of freedom parameter and scale parameter.

Parameters
:   |  |  |
    | --- | --- |
    | y | A scalar variable. |
    | nu | Degrees of freedom. |
    | s | Scale parameter. |

Exceptions
:   |  |  |
    | --- | --- |
    | std::domain\_error | if nu is not greater than 0 |
    | std::domain\_error | if s is not greater than 0. |
    | std::domain\_error | if y is not greater than 0. |

Template Parameters
:   |  |  |
    | --- | --- |
    | T\_y | Type of scalar. |
    | T\_dof | Type of degrees of freedom. |

Definition at line 187 of file scaled\_inv\_chi\_square.hpp.

template<typename T\_y , typename T\_dof , typename T\_scale >

|  |  |  |  |
| --- | --- | --- | --- |
| return\_type<T\_y, T\_dof, T\_scale>::type stan::prob::scaled\_inv\_chi\_square\_cdf\_log | ( | const T\_y & | *y*, |
|  |  | const T\_dof & | *nu*, |
|  |  | const T\_scale & | *s* |
|  | ) |  |  |

Definition at line 310 of file scaled\_inv\_chi\_square.hpp.

template<bool propto, typename T\_y , typename T\_dof , typename T\_scale >

|  |  |  |  |
| --- | --- | --- | --- |
| return\_type<T\_y,T\_dof,T\_scale>::type stan::prob::scaled\_inv\_chi\_square\_log | ( | const T\_y & | *y*, |
|  |  | const T\_dof & | *nu*, |
|  |  | const T\_scale & | *s* |
|  | ) |  |  |

The log of a scaled inverse chi-squared density for y with the specified degrees of freedom parameter and scale parameter.

Parameters
:   |  |  |
    | --- | --- |
    | y | A scalar variable. |
    | nu | Degrees of freedom. |
    | s | Scale parameter. |

Exceptions
:   |  |  |
    | --- | --- |
    | std::domain\_error | if nu is not greater than 0 |
    | std::domain\_error | if s is not greater than 0. |
    | std::domain\_error | if y is not greater than 0. |

Template Parameters
:   |  |  |
    | --- | --- |
    | T\_y | Type of scalar. |
    | T\_dof | Type of degrees of freedom. |

Definition at line 44 of file scaled\_inv\_chi\_square.hpp.

template<typename T\_y , typename T\_dof , typename T\_scale >

|  |  |  |  |
| --- | --- | --- | --- |
| return\_type<T\_y,T\_dof,T\_scale>::type stan::prob::scaled\_inv\_chi\_square\_log | ( | const T\_y & | *y*, |
|  |  | const T\_dof & | *nu*, |
|  |  | const T\_scale & | *s* |
|  | ) |  |  |

inline

Definition at line 167 of file scaled\_inv\_chi\_square.hpp.

template<class RNG >

|  |  |  |  |
| --- | --- | --- | --- |
| double stan::prob::scaled\_inv\_chi\_square\_rng | ( | const double | *nu*, |
|  |  | const double | *s*, |
|  |  | RNG & | *rng* |
|  | ) |  |  |

inline

Definition at line 528 of file scaled\_inv\_chi\_square.hpp.

template<typename T >

|  |  |  |  |  |  |
| --- | --- | --- | --- | --- | --- |
| Eigen::Matrix<T,Eigen::Dynamic,1> stan::prob::simplex\_constrain | ( | const Eigen::Matrix< T, Eigen::Dynamic, 1 > & | *y* | ) |  |

Return the simplex corresponding to the specified free vector.

A simplex is a vector containing values greater than or equal to 0 that sum to 1. A vector with (K-1) unconstrained values will produce a simplex of size K.

The transform is based on a centered stick-breaking process.

Parameters
:   |  |  |
    | --- | --- |
    | y | Free vector input of dimensionality K - 1. |

Returns
:   Simplex of dimensionality K.

Template Parameters
:   |  |  |
    | --- | --- |
    | T | Type of scalar. |

Definition at line 1103 of file transform.hpp.

template<typename T >

|  |  |  |  |
| --- | --- | --- | --- |
| Eigen::Matrix<T,Eigen::Dynamic,1> stan::prob::simplex\_constrain | ( | const Eigen::Matrix< T, Eigen::Dynamic, 1 > & | *y*, |
|  |  | T & | *lp* |
|  | ) |  |  |

Return the simplex corresponding to the specified free vector and increment the specified log probability reference with the log absolute Jacobian determinant of the transform.

The simplex transform is defined through a centered stick-breaking process.

Parameters
:   |  |  |
    | --- | --- |
    | y | Free vector input of dimensionality K - 1. |
    | lp | Log probability reference to increment. |

Returns
:   Simplex of dimensionality K.

Template Parameters
:   |  |  |
    | --- | --- |
    | T | Type of scalar. |

Definition at line 1142 of file transform.hpp.

template<typename T >

|  |  |  |  |  |  |
| --- | --- | --- | --- | --- | --- |
| Eigen::Matrix<T,Eigen::Dynamic,1> stan::prob::simplex\_free | ( | const Eigen::Matrix< T, Eigen::Dynamic, 1 > & | *x* | ) |  |

Return an unconstrained vector that when transformed produces the specified simplex.

It applies to a simplex of dimensionality K and produces an unconstrained vector of dimensionality (K-1).

The simplex transform is defined through a centered stick-breaking process.

Parameters
:   |  |  |
    | --- | --- |
    | x | Simplex of dimensionality K. |

Returns
:   Free vector of dimensionality (K-1) that transfroms to the simplex.

Template Parameters
:   |  |  |
    | --- | --- |
    | T | Type of scalar. |

Exceptions
:   |  |  |
    | --- | --- |
    | std::domain\_error | if x is not a valid simplex |

Definition at line 1188 of file transform.hpp.

template<typename T\_y , typename T\_loc , typename T\_scale , typename T\_shape >

|  |  |  |  |
| --- | --- | --- | --- |
| return\_type<T\_y,T\_loc,T\_scale,T\_shape>::type stan::prob::skew\_normal\_ccdf\_log | ( | const T\_y & | *y*, |
|  |  | const T\_loc & | *mu*, |
|  |  | const T\_scale & | *sigma*, |
|  |  | const T\_shape & | *alpha* |
|  | ) |  |  |

Definition at line 320 of file skew\_normal.hpp.

template<typename T\_y , typename T\_loc , typename T\_scale , typename T\_shape >

|  |  |  |  |
| --- | --- | --- | --- |
| return\_type<T\_y,T\_loc,T\_scale,T\_shape>::type stan::prob::skew\_normal\_cdf | ( | const T\_y & | *y*, |
|  |  | const T\_loc & | *mu*, |
|  |  | const T\_scale & | *sigma*, |
|  |  | const T\_shape & | *alpha* |
|  | ) |  |  |

Definition at line 141 of file skew\_normal.hpp.

template<typename T\_y , typename T\_loc , typename T\_scale , typename T\_shape >

|  |  |  |  |
| --- | --- | --- | --- |
| return\_type<T\_y,T\_loc,T\_scale,T\_shape>::type stan::prob::skew\_normal\_cdf\_log | ( | const T\_y & | *y*, |
|  |  | const T\_loc & | *mu*, |
|  |  | const T\_scale & | *sigma*, |
|  |  | const T\_shape & | *alpha* |
|  | ) |  |  |

Definition at line 237 of file skew\_normal.hpp.

template<bool propto, typename T\_y , typename T\_loc , typename T\_scale , typename T\_shape >

|  |  |  |  |
| --- | --- | --- | --- |
| return\_type<T\_y,T\_loc,T\_scale,T\_shape>::type stan::prob::skew\_normal\_log | ( | const T\_y & | *y*, |
|  |  | const T\_loc & | *mu*, |
|  |  | const T\_scale & | *sigma*, |
|  |  | const T\_shape & | *alpha* |
|  | ) |  |  |

Definition at line 23 of file skew\_normal.hpp.

template<typename T\_y , typename T\_loc , typename T\_scale , typename T\_shape >

|  |  |  |  |
| --- | --- | --- | --- |
| return\_type<T\_y,T\_loc,T\_scale, T\_shape>::type stan::prob::skew\_normal\_log | ( | const T\_y & | *y*, |
|  |  | const T\_loc & | *mu*, |
|  |  | const T\_scale & | *sigma*, |
|  |  | const T\_shape & | *alpha* |
|  | ) |  |  |

inline

Definition at line 134 of file skew\_normal.hpp.

template<class RNG >

|  |  |  |  |
| --- | --- | --- | --- |
| double stan::prob::skew\_normal\_rng | ( | const double | *mu*, |
|  |  | const double | *sigma*, |
|  |  | const double | *alpha*, |
|  |  | RNG & | *rng* |
|  | ) |  |  |

inline

Definition at line 403 of file skew\_normal.hpp.

template<typename T\_y , typename T\_dof , typename T\_loc , typename T\_scale >

|  |  |  |  |
| --- | --- | --- | --- |
| return\_type<T\_y, T\_dof, T\_loc, T\_scale>::type stan::prob::student\_t\_ccdf\_log | ( | const T\_y & | *y*, |
|  |  | const T\_dof & | *nu*, |
|  |  | const T\_loc & | *mu*, |
|  |  | const T\_scale & | *sigma* |
|  | ) |  |  |

Definition at line 555 of file student\_t.hpp.

template<typename T\_y , typename T\_dof , typename T\_loc , typename T\_scale >

|  |  |  |  |
| --- | --- | --- | --- |
| return\_type<T\_y, T\_dof, T\_loc, T\_scale>::type stan::prob::student\_t\_cdf | ( | const T\_y & | *y*, |
|  |  | const T\_dof & | *nu*, |
|  |  | const T\_loc & | *mu*, |
|  |  | const T\_scale & | *sigma* |
|  | ) |  |  |

Definition at line 219 of file student\_t.hpp.

template<typename T\_y , typename T\_dof , typename T\_loc , typename T\_scale >

|  |  |  |  |
| --- | --- | --- | --- |
| return\_type<T\_y, T\_dof, T\_loc, T\_scale>::type stan::prob::student\_t\_cdf\_log | ( | const T\_y & | *y*, |
|  |  | const T\_dof & | *nu*, |
|  |  | const T\_loc & | *mu*, |
|  |  | const T\_scale & | *sigma* |
|  | ) |  |  |

Definition at line 393 of file student\_t.hpp.

template<bool propto, typename T\_y , typename T\_dof , typename T\_loc , typename T\_scale >

|  |  |  |  |
| --- | --- | --- | --- |
| return\_type<T\_y,T\_dof,T\_loc,T\_scale>::type stan::prob::student\_t\_log | ( | const T\_y & | *y*, |
|  |  | const T\_dof & | *nu*, |
|  |  | const T\_loc & | *mu*, |
|  |  | const T\_scale & | *sigma* |
|  | ) |  |  |

The log of the Student-t density for the given y, nu, mean, and scale parameter.

The scale parameter must be greater than 0.

Parameters
:   |  |  |
    | --- | --- |
    | y | A scalar variable. |
    | nu | Degrees of freedom. |
    | mu | The mean of the Student-t distribution. |
    | sigma | The scale parameter of the Student-t distribution. |

Returns
:   The log of the Student-t density at y.

Exceptions
:   |  |  |
    | --- | --- |
    | std::domain\_error | if sigma is not greater than 0. |
    | std::domain\_error | if nu is not greater than 0. |

Template Parameters
:   |  |  |
    | --- | --- |
    | T\_y | Type of scalar. |
    | T\_dof | Type of degrees of freedom. |
    | T\_loc | Type of location. |
    | T\_scale | Type of scale. |

Definition at line 49 of file student\_t.hpp.

template<typename T\_y , typename T\_dof , typename T\_loc , typename T\_scale >

|  |  |  |  |
| --- | --- | --- | --- |
| return\_type<T\_y,T\_dof,T\_loc,T\_scale>::type stan::prob::student\_t\_log | ( | const T\_y & | *y*, |
|  |  | const T\_dof & | *nu*, |
|  |  | const T\_loc & | *mu*, |
|  |  | const T\_scale & | *sigma* |
|  | ) |  |  |

inline

Definition at line 212 of file student\_t.hpp.

template<class RNG >

|  |  |  |  |
| --- | --- | --- | --- |
| double stan::prob::student\_t\_rng | ( | const double | *nu*, |
|  |  | const double | *mu*, |
|  |  | const double | *sigma*, |
|  |  | RNG & | *rng* |
|  | ) |  |  |

inline

Definition at line 717 of file student\_t.hpp.

template<typename T , typename TU >

|  |  |  |  |
| --- | --- | --- | --- |
| boost::math::tools::promote\_args<T,TU>::type stan::prob::ub\_constrain | ( | const T | *x*, |
|  |  | const TU | *ub* |
|  | ) |  |  |

inline

Return the upper-bounded value for the specified unconstrained scalar and upper bound.

The transform is

where  is the upper bound.

If the upper bound is positive infinity, this function reduces to `identity_constrain(x)`.

Parameters
:   |  |  |
    | --- | --- |
    | x | Free scalar. |
    | ub | Upper bound. |

Returns
:   Transformed scalar with specified upper bound.

Template Parameters
:   |  |  |
    | --- | --- |
    | T | Type of scalar. |
    | TU | Type of upper bound. |

Definition at line 604 of file transform.hpp.

template<typename T , typename TU >

|  |  |  |  |
| --- | --- | --- | --- |
| boost::math::tools::promote\_args<T,TU>::type stan::prob::ub\_constrain | ( | const T | *x*, |
|  |  | const TU | *ub*, |
|  |  | T & | *lp* |
|  | ) |  |  |

inline

Return the upper-bounded value for the specified unconstrained scalar and upper bound and increment the specified log probability reference with the log absolute Jacobian determinant of the transform.

The transform is as specified for `ub_constrain(T,double)`. The log absolute Jacobian determinant is

.

If the upper bound is positive infinity, this function reduces to `identity_constrain(x,lp)`.

Parameters
:   |  |  |
    | --- | --- |
    | x | Free scalar. |
    | ub | Upper bound. |
    | lp | Log probability reference. |

Returns
:   Transformed scalar with specified upper bound.

Template Parameters
:   |  |  |
    | --- | --- |
    | T | Type of scalar. |
    | TU | Type of upper bound. |

Definition at line 636 of file transform.hpp.

template<typename T , typename TU >

|  |  |  |  |
| --- | --- | --- | --- |
| boost::math::tools::promote\_args<T,TU>::type stan::prob::ub\_free | ( | const T | *y*, |
|  |  | const TU | *ub* |
|  | ) |  |  |

inline

Return the free scalar that corresponds to the specified upper-bounded value with respect to the specified upper bound.

The transform is the reverse of the `ub_constrain(T,double)` transform,

where  is the upper bound.

If the upper bound is positive infinity, this function reduces to `identity_free(y)`.

Parameters
:   |  |  |
    | --- | --- |
    | y | Upper-bounded scalar. |
    | ub | Upper bound. |

Returns
:   Free scalar corresponding to upper-bounded scalar.

Template Parameters
:   |  |  |
    | --- | --- |
    | T | Type of scalar. |
    | TU | Type of upper bound. |

Exceptions
:   |  |  |
    | --- | --- |
    | std::invalid\_argument | if y is greater than the upper bound. |

Definition at line 668 of file transform.hpp.

template<typename T\_y , typename T\_low , typename T\_high >

|  |  |  |  |
| --- | --- | --- | --- |
| return\_type<T\_y,T\_low,T\_high>::type stan::prob::uniform\_ccdf\_log | ( | const T\_y & | *y*, |
|  |  | const T\_low & | *alpha*, |
|  |  | const T\_high & | *beta* |
|  | ) |  |  |

Definition at line 270 of file uniform.hpp.

template<typename T\_y , typename T\_low , typename T\_high >

|  |  |  |  |
| --- | --- | --- | --- |
| return\_type<T\_y,T\_low,T\_high>::type stan::prob::uniform\_cdf | ( | const T\_y & | *y*, |
|  |  | const T\_low & | *alpha*, |
|  |  | const T\_high & | *beta* |
|  | ) |  |  |

Definition at line 125 of file uniform.hpp.

template<typename T\_y , typename T\_low , typename T\_high >

|  |  |  |  |
| --- | --- | --- | --- |
| return\_type<T\_y,T\_low,T\_high>::type stan::prob::uniform\_cdf\_log | ( | const T\_y & | *y*, |
|  |  | const T\_low & | *alpha*, |
|  |  | const T\_high & | *beta* |
|  | ) |  |  |

Definition at line 201 of file uniform.hpp.

template<bool propto, typename T\_y , typename T\_low , typename T\_high >

|  |  |  |  |
| --- | --- | --- | --- |
| return\_type<T\_y,T\_low,T\_high>::type stan::prob::uniform\_log | ( | const T\_y & | *y*, |
|  |  | const T\_low & | *alpha*, |
|  |  | const T\_high & | *beta* |
|  | ) |  |  |

The log of a uniform density for the given y, lower, and upper bound.

Parameters
:   |  |  |
    | --- | --- |
    | y | A scalar variable. |
    | alpha | Lower bound. |
    | beta | Upper bound. |

Exceptions
:   |  |  |
    | --- | --- |
    | std::invalid\_argument | if the lower bound is greater than or equal to the lower bound |

Template Parameters
:   |  |  |
    | --- | --- |
    | T\_y | Type of scalar. |
    | T\_low | Type of lower bound. |
    | T\_high | Type of upper bound. |

Definition at line 44 of file uniform.hpp.

template<typename T\_y , typename T\_low , typename T\_high >

|  |  |  |  |
| --- | --- | --- | --- |
| return\_type<T\_y,T\_low,T\_high>::type stan::prob::uniform\_log | ( | const T\_y & | *y*, |
|  |  | const T\_low & | *alpha*, |
|  |  | const T\_high & | *beta* |
|  | ) |  |  |

inline

Definition at line 119 of file uniform.hpp.

template<class RNG >

|  |  |  |  |
| --- | --- | --- | --- |
| double stan::prob::uniform\_rng | ( | const double | *alpha*, |
|  |  | const double | *beta*, |
|  |  | RNG & | *rng* |
|  | ) |  |  |

inline

Definition at line 339 of file uniform.hpp.

template<typename T >

|  |  |  |  |  |  |
| --- | --- | --- | --- | --- | --- |
| Eigen::Matrix<T,Eigen::Dynamic,1> stan::prob::unit\_vector\_constrain | ( | const Eigen::Matrix< T, Eigen::Dynamic, 1 > & | *y* | ) |  |

Return the unit length vector corresponding to the free vector y.

The free vector contains K-1 spherical coordinates.

Parameters
:   |  |  |
    | --- | --- |
    | y | of K - 1 spherical coordinates |

Returns
:   Unit length vector of dimension K

Template Parameters
:   |  |  |
    | --- | --- |
    | T | Scalar type. |

Definition at line 1015 of file transform.hpp.

template<typename T >

|  |  |  |  |
| --- | --- | --- | --- |
| Eigen::Matrix<T,Eigen::Dynamic,1> stan::prob::unit\_vector\_constrain | ( | const Eigen::Matrix< T, Eigen::Dynamic, 1 > & | *y*, |
|  |  | T & | *lp* |
|  | ) |  |  |

Return the unit length vector corresponding to the free vector y.

The free vector contains K-1 spherical coordinates.

Parameters
:   |  |  |
    | --- | --- |
    | y | of K - 1 spherical coordinates |

Returns
:   Unit length vector of dimension K

Parameters
:   |  |  |
    | --- | --- |
    | lp | Log probability reference to increment. |

Template Parameters
:   |  |  |
    | --- | --- |
    | T | Scalar type. |

Definition at line 1044 of file transform.hpp.

template<typename T >

|  |  |  |  |  |  |
| --- | --- | --- | --- | --- | --- |
| Eigen::Matrix<T,Eigen::Dynamic,1> stan::prob::unit\_vector\_free | ( | const Eigen::Matrix< T, Eigen::Dynamic, 1 > & | *x* | ) |  |

Definition at line 1067 of file transform.hpp.

template<bool propto, typename T\_y , typename T\_loc , typename T\_scale >

|  |  |  |  |
| --- | --- | --- | --- |
| return\_type<T\_y,T\_loc,T\_scale>::type stan::prob::von\_mises\_log | ( | T\_y const & | *y*, |
|  |  | T\_loc const & | *mu*, |
|  |  | T\_scale const & | *kappa* |
|  | ) |  |  |

Definition at line 19 of file von\_mises.hpp.

template<typename T\_y , typename T\_loc , typename T\_scale >

|  |  |  |  |
| --- | --- | --- | --- |
| return\_type<T\_y,T\_loc,T\_scale>::type stan::prob::von\_mises\_log | ( | T\_y const & | *y*, |
|  |  | T\_loc const & | *mu*, |
|  |  | T\_scale const & | *kappa* |
|  | ) |  |  |

inline

Definition at line 115 of file von\_mises.hpp.

template<class RNG >

|  |  |  |  |
| --- | --- | --- | --- |
| double stan::prob::von\_mises\_rng | ( | const double | *mu*, |
|  |  | const double | *kappa*, |
|  |  | RNG & | *rng* |
|  | ) |  |  |

inline

Definition at line 130 of file von\_mises.hpp.

template<typename T\_y , typename T\_shape , typename T\_scale >

|  |  |  |  |
| --- | --- | --- | --- |
| return\_type<T\_y,T\_shape,T\_scale>::type stan::prob::weibull\_ccdf\_log | ( | const T\_y & | *y*, |
|  |  | const T\_shape & | *alpha*, |
|  |  | const T\_scale & | *sigma* |
|  | ) |  |  |

Definition at line 254 of file weibull.hpp.

template<typename T\_y , typename T\_shape , typename T\_scale >

|  |  |  |  |
| --- | --- | --- | --- |
| return\_type<T\_y,T\_shape,T\_scale>::type stan::prob::weibull\_cdf | ( | const T\_y & | *y*, |
|  |  | const T\_shape & | *alpha*, |
|  |  | const T\_scale & | *sigma* |
|  | ) |  |  |

Definition at line 138 of file weibull.hpp.

template<typename T\_y , typename T\_shape , typename T\_scale >

|  |  |  |  |
| --- | --- | --- | --- |
| return\_type<T\_y,T\_shape,T\_scale>::type stan::prob::weibull\_cdf\_log | ( | const T\_y & | *y*, |
|  |  | const T\_shape & | *alpha*, |
|  |  | const T\_scale & | *sigma* |
|  | ) |  |  |

Definition at line 201 of file weibull.hpp.

template<bool propto, typename T\_y , typename T\_shape , typename T\_scale >

|  |  |  |  |
| --- | --- | --- | --- |
| return\_type<T\_y,T\_shape,T\_scale>::type stan::prob::weibull\_log | ( | const T\_y & | *y*, |
|  |  | const T\_shape & | *alpha*, |
|  |  | const T\_scale & | *sigma* |
|  | ) |  |  |

Definition at line 24 of file weibull.hpp.

template<typename T\_y , typename T\_shape , typename T\_scale >

|  |  |  |  |
| --- | --- | --- | --- |
| return\_type<T\_y,T\_shape,T\_scale>::type stan::prob::weibull\_log | ( | const T\_y & | *y*, |
|  |  | const T\_shape & | *alpha*, |
|  |  | const T\_scale & | *sigma* |
|  | ) |  |  |

inline

Definition at line 132 of file weibull.hpp.

template<class RNG >

|  |  |  |  |
| --- | --- | --- | --- |
| double stan::prob::weibull\_rng | ( | const double | *alpha*, |
|  |  | const double | *sigma*, |
|  |  | RNG & | *rng* |
|  | ) |  |  |

inline

Definition at line 304 of file weibull.hpp.

template<bool propto, typename T\_y , typename T\_dof , typename T\_scale >

|  |  |  |  |
| --- | --- | --- | --- |
| boost::math::tools::promote\_args<T\_y,T\_dof,T\_scale>::type stan::prob::wishart\_log | ( | const Eigen::Matrix< T\_y, Eigen::Dynamic, Eigen::Dynamic > & | *W*, |
|  |  | const T\_dof & | *nu*, |
|  |  | const Eigen::Matrix< T\_scale, Eigen::Dynamic, Eigen::Dynamic > & | *S* |
|  | ) |  |  |

The log of the Wishart density for the given W, degrees of freedom, and scale matrix.

The scale matrix, S, must be k x k, symmetric, and semi-positive definite. Dimension, k, is implicit. nu must be greater than k-1

Parameters
:   |  |  |
    | --- | --- |
    | W | A scalar matrix |
    | nu | Degrees of freedom |
    | S | The scale matrix |

Returns
:   The log of the Wishart density at W given nu and S.

Exceptions
:   |  |  |
    | --- | --- |
    | std::domain\_error | if nu is not greater than k-1 |
    | std::domain\_error | if S is not square, not symmetric, or not semi-positive definite. |

Template Parameters
:   |  |  |
    | --- | --- |
    | T\_y | Type of scalar. |
    | T\_dof | Type of degrees of freedom. |
    | T\_scale | Type of scale. |

Definition at line 62 of file wishart.hpp.

template<typename T\_y , typename T\_dof , typename T\_scale >

|  |  |  |  |
| --- | --- | --- | --- |
| boost::math::tools::promote\_args<T\_y,T\_dof,T\_scale>::type stan::prob::wishart\_log | ( | const Eigen::Matrix< T\_y, Eigen::Dynamic, Eigen::Dynamic > & | *W*, |
|  |  | const T\_dof & | *nu*, |
|  |  | const Eigen::Matrix< T\_scale, Eigen::Dynamic, Eigen::Dynamic > & | *S* |
|  | ) |  |  |

inline

Definition at line 135 of file wishart.hpp.

template<class RNG >

|  |  |  |  |
| --- | --- | --- | --- |
| Eigen::Matrix<double, Eigen::Dynamic, Eigen::Dynamic> stan::prob::wishart\_rng | ( | const double | *nu*, |
|  |  | const Eigen::Matrix< double, Eigen::Dynamic, Eigen::Dynamic > & | *S*, |
|  |  | RNG & | *rng* |
|  | ) |  |  |

inline

Definition at line 143 of file wishart.hpp.

## Variable Documentation

const double stan::prob::CONSTRAINT\_TOLERANCE = 1E-8

Definition at line 32 of file transform.hpp.


---

[ Stan Home Page ]

*© 2011–2014,
Stan Development Team.*
